# Simulation of Multispecies Desmoplastic Cancer Growth via a Fully Adaptive Non-linear Full Multigrid Algorithm

**DOI:** 10.3389/fphys.2018.00821

**Published:** 2018-07-12

**Authors:** Chin F. Ng, Hermann B. Frieboes

**Affiliations:** ^1^Department of Bioengineering, University of Louisville, Louisville, KY, United States; ^2^James Graham Brown Cancer Center, University of Louisville, Louisville, KY, United States

**Keywords:** cancer, computational simulation, mathematical model, non-linear 3D tumor growth, diffuse interface model, adaptive mesh refinement, full multigrid, full approximation scheme

## Abstract

A fully adaptive non-linear full multigrid (FMG) algorithm is implemented to computationally simulate a model of multispecies desmoplastic tumor growth in three spatial dimensions. The algorithm solves a thermodynamic mixture model employing a diffuse interface approach with Cahn-Hilliard-type fourth-order equations that are coupled, non-linear, and numerically stiff. The tumor model includes extracellular matrix (ECM) as a major component with elastic energy contribution in its chemical potential term. Blood and lymphatic vasculatures are simulated via continuum representations. The model employs advection-reaction-diffusion partial differential equations (PDEs) for the cell, ECM, and vascular components, and reaction-diffusion PDEs for the elements diffusing from the vessels. This study provides the details of the numerical solution obtained by applying the fully adaptive non-linear FMG algorithm with finite difference method to solve this complex system of PDEs. The results indicate that this type of computational model can simulate the extracellular matrix-rich desmoplastic tumor microenvironment typical of fibrotic tumors, such as pancreatic adenocarcinoma.

## Introduction

The process of cancer progression is driven by the communication between tumor cells and their surroundings. A dynamic tumor microenvironment typically consists of highly proliferating neoplastic cells of different phenotypes, necrotic tumor cells, infiltrating innate and adaptive immune inflammatory cells, cancer-associated fibroblasts, cancer stem cells, extracellular matrix (ECM), blood and lymphatic vessels, pericytes, healthy host cells, and a variety of soluble molecules (Hanahan and Weinberg, 2000; de Visser and Coussens, 2006; Tlsty and Coussens, 2006; Whiteside, 2008; Perez-Moreno, 2009). These cellular and molecular elements dictate the tumor progress from unregulated neoplastic growth to potential metastasis. In its heterogeneous milieu with complex tumor-induced interactions and mechanical stress, an evolving tumor mass also undergoes transient morphological changes arising from cell motility and cell-cell/cell-ECM interactions. Mathematical modeling of cancer progression including its associated microenvironment may be a useful tool for predicting tumor dynamics and cancer response to therapy.

To study the desmoplastic tumor microenvironment, we recently presented a tumor model (Ng and Frieboes, 2017) as a continuum scale multicomponent-multispecies system consisting of heterogeneous cell types and ECM. This thermodynamic mixture model, inspired by the one derived in Wise et al. (2008) and Frieboes et al. (2010), includes metabolic reactions, tumorigenic factors, desmoplastic response, as well as tumor-induced angiogenesis and lymphangiogenesis. The diffuse interface approach is implemented as derived in Wise et al. (2008), where thermodynamically consistent Darcy velocities and Fickian diffusive terms are determined from the energy variation. In the Helmholtz free energy equation, the square gradient model (Cahn and Hilliard, 1958; Cahn, 1959; Yang et al., 1976; Rowlinson, 1979) is used to describe interfaces arising from the adhesive properties of cells and ECM components, and an elastic energy term by Leo et al. (1998) is added to represent the elastic properties of the ECM component. Continuous blood and lymphatic vessel densities are modified from cell fluxes employed in Anderson and Chaplain (1998), Chaplain (1996), and Mantzaris et al. (2004). Sprout initiation conditions of vessels, as well as interactions between angiogenic factors, proteolytic enzymes, and the ECM component are inspired by Levine et al. (2000), Levine et al. (2001a,b). Lymphangiogenesis is assumed to behave similarly to angiogenesis. Nutrients and waste products from cell metabolism are governed by fluxes and reaction rates modified from Casciari et al. (1992).

Continuum models represent cell populations and molecular species that influence the cell cycle events as continuous variables (see recent reviews Roose et al., 2007; Preziosi and Tosin, 2009a; Tracqui, 2009; Byrne, 2010; Cristini and Lowengrub, 2010; Edelman et al., 2010; Kreeger and Lauffenburger, 2010; Lowengrub et al., 2010; Osborne et al., 2010; Rejniak and McCawley, 2010; Vineis et al., 2010; Andasari et al., 2011; Chaplain, 2011; Deisboeck et al., 2011; Frieboes et al., 2011; Michor et al., 2011; Rejniak and Anderson, 2011; Bachmann et al., 2012; Oden et al., 2015 and references therein), These models typically implement ODE or PDE approaches to describe an advection-diffusion-reaction system. Continuum multiphase/mixture mechanochemical models include chemical and mechanical interactions between phases (cell types or species) (see Araujo and McElwain, 2004; Hatzikirou et al., 2005; Quaranta et al., 2005; Byrne et al., 2006; Graziano and Preziosi, 2007; Roose et al., 2007; Astanin and Preziosi, 2008; Preziosi and Tosin, 2009a; Tracqui, 2009; Lowengrub et al., 2010) and associated references). These models typically introduce a stress tensor, velocity, and pressure for each phase by enforcing mass, momentum, and energy balances (Ambrosi and Preziosi, 2002; Breward et al., 2002, 2003; Byrne and Preziosi, 2003; Byrne et al., 2003; Araujo and McElwain, 2005a,b; Graziano and Preziosi, 2007; Astanin and Preziosi, 2008; Galle et al., 2009; Preziosi and Tosin, 2009b; Bresch et al., 2010; Preziosi et al., 2010; Preziosi and Vitale, 2011; Sciumé et al., 2013; Klika, 2014). Related to these mixture models is the diffuse interface approach (Oden et al., 2010; Hawkins-Daarud et al., 2012; Chen et al., 2014), for which the square gradient theory can be used to describe smooth transitions within thin interfacial regions. The gradient contributes to the Helmholtz free energy, from which the component velocities, pressures, and diffusive terms can be derived (Wise et al., 2008; Chen and Lowengrub, 2014). Continuum single- or multi-phase models that consider the effects of cell-cell and/or cell-ECM adhesion have included (Frieboes et al., 2007, 2013; Ambrosi and Preziosi, 2009; Bearer et al., 2009; Kuusela and Alt, 2009; Chatelain Clément et al., 2011; Escher and Matioc, 2013). In Gerisch and Chaplain (2008), Preziosi and Tosin (2009b), Psiuk-Maksymowicz (2013), Wu et al. (2013), Sciumé et al. (2014a,b), and Arduino and Preziosi (2015), the ECM is represented as a key component of the tumoral tissue.

This paper presents the fully adaptive non-linear FMG algorithm and numerical solution of the model presented in Ng and Frieboes (2017). This diffuse interface model is characterized by non-linear fourth-order Cahn-Hilliard type PDEs and narrow transition layers. A fully adaptive block-structure Cartesian mesh is used on adaptively refined grid levels to address the need of transient locally-refined mesh regions for the narrow transition layers. To avoid severe time-step restrictions of explicit methods and fine resolution of transition layers, a fully adaptive non-linear finite difference multigrid method inspired by the pioneering work of Wise et al. (2007) and Wise et al. (2011) is implemented to solve the system of equations. The equations are discretized in time by the semi-implicit Crank-Nicolson method. The error smoothing steps employ the non-linear Full Approximation Scheme, and iterations are carried out in V-cycles. Modified from the numerical solution and method given in Wise et al. (2007, 2011) we implement a fully adaptive non-linear full multigrid (FMG) algorithm with finite difference method to solve this complex set of PDEs.

## Materials and methods

### Desmoplastic tumor model

Consider a tumor growing in a tissue domain Ω ⊂ ℝ^3^ where tumor and healthy cells, as well as the ECM, are tracked with continuous volume fractions. In the diffuse interface model, adhesive forces hold the tumor cells together, creating a boundary layer of finite thickness between the tumoral and healthy regions. An appropriate distribution of ECM across the domain can be achieved by a carefully chosen free energy term. Dimensionless governing equations for multispecies tumor growth systems are presented here.

We assume that the liquid (extracellular fluid) volume fraction stays constant, as well as the total solid (ECM, tumor and healthy cells) volume fraction, and set the densities of all components to unity. Dimensionless variables ${\tilde{\phi }}_{V}$, ${\tilde{\phi }}_{D}$, ${\tilde{\phi }}_{E}$, and ${\tilde{\phi }}_{H}$ are the normalized volume fraction (normalized by the total volume fraction) of viable tumor cells, dead tumor cells, ECM, and healthy host cells, respectively. Their transient diffusion-convection governing equations are given as Ng and Frieboes (2017):

#### Cells and ECM components

(2.1) $$\begin{array}{lll} \frac{\partial {\tilde{\phi }}_{V}}{\partial \tilde{t}}+\nabla \cdot \left({\tilde{\phi }}_{V}{\tilde{\text{u}}}_{\alpha }\right) & = & \nabla \cdot \left({\tilde{M}}_{V}\nabla {\tilde{\mu }}_{T}\right)+{\tilde{S}}_{V} \end{array}$$

(2.2) $$\begin{array}{lll} \frac{\partial {\tilde{\phi }}_{D}}{\partial \tilde{t}}+\nabla \cdot \left({\tilde{\phi }}_{D}{\tilde{\text{u}}}_{\alpha }\right) & = & \nabla \cdot \left({\tilde{M}}_{D}\nabla {\tilde{\mu }}_{T}\right)+{\tilde{S}}_{D} \end{array}$$

(2.3) $$\begin{array}{lll} \frac{\partial {\tilde{\phi }}_{E}}{\partial \tilde{t}}+\nabla \cdot \left({\tilde{\phi }}_{E}{\tilde{\text{u}}}_{\alpha }\right) & = & \nabla \cdot \left({\tilde{M}}_{E}\nabla {\tilde{\mu }}_{E}\right)+{\tilde{S}}_{E} \end{array}$$

(2.4) $$\begin{array}{lll} {\tilde{\phi }}_{H} & = & 1-{\tilde{\phi }}_{V}-{\tilde{\phi }}_{D}-{\tilde{\phi }}_{E} \end{array}$$

The total tumor volume fraction is ${\tilde{\phi }}_{T}={\tilde{\phi }}_{V}+{\tilde{\phi }}_{D}$ and ${\tilde{M}}_{i}=\tilde{M}{\tilde{\phi }}_{i}$ is the positive non-constant mobility of component *i*. Chemical potential terms are given below:

#### Chemical potentials

(2.5) $$\begin{array}{lll} {\tilde{\mu }}_{T} & = & \frac{\partial {\tilde{F}}_{b}}{\partial {\tilde{\phi }}_{T}}-{{\tilde{\epsilon }}_{T}}^{2}\;\nabla^{2}{\tilde{\phi }}_{T}-{{\tilde{\epsilon }}_{TE}}^{2}\nabla^{2}{\tilde{\phi }}_{E} \end{array}$$

(2.6) $$\begin{array}{lll} {\tilde{\mu }}_{E} & = & \frac{\partial {\tilde{F}}_{b}}{\partial {\tilde{\phi }}_{E}}+\frac{\partial \tilde{W}}{\partial {\tilde{\phi }}_{E}}-{{\tilde{\epsilon }}_{E}}^{2}\;\nabla^{2}{\tilde{\phi }}_{E}-{{\tilde{\epsilon }}_{TE}}^{2}\nabla^{2}{\tilde{\phi }}_{T} \end{array}$$

(2.7) $$\begin{array}{l} \frac{\partial {\tilde{F}}_{b}}{\partial {\tilde{\phi }}_{T}}=2A_{1}{\tilde{\phi }}_{T}\left(1-{\tilde{\phi }}_{T}-{\tilde{\phi }}_{E}\right)\left(1-2{\tilde{\phi }}_{T}-{\tilde{\phi }}_{E}\right)\\ \;+\left(A_{5}-A_{3}\right)\left(2{\tilde{\phi }}_{E}-A_{5}-A_{3}\right) \end{array}$$

(2.8) $$\begin{array}{l} \frac{\partial {\tilde{F}}_{b}}{\partial {\tilde{\phi }}_{E}}=2\left({\tilde{\phi }}_{T}+A_{2}\right)\left({\tilde{\phi }}_{E}-A_{3}\right)-2A_{1}{\left({\tilde{\phi }}_{T}\right)}^{2}\left(1-{\tilde{\phi }}_{T}-{\tilde{\phi }}_{E}\right)\\ \;+\;\left({\tilde{\phi }}_{E}-A_{5}\right)\left[2\left(1-{\tilde{\phi }}_{T}+A_{4}\right)-3{\tilde{\phi }}_{E}+A_{5}\right] \end{array}$$

(2.9) $$\begin{array}{lll} \frac{\partial \tilde{W}}{\partial {\tilde{\phi }}_{E}} & = & {\tilde{\epsilon }}_{e}\left[6{\tilde{\phi }}_{E}\left(1-{\tilde{\phi }}_{E}\right)\right]\overset{3}{\underset{i,j=1}{\sum }}\left[\frac{1}{2}{\left({\tilde{E}}_{T}\right)}_{ij}{\tilde{T}}_{ij}^{{\;}^{*}}-{\left({\tilde{E}}_{T}^{{\;}^{*}}\right)}_{ij}{\tilde{𝕋}}_{ij}\right] \end{array}$$

The bulk free energy term ${\tilde{F}}_{b}$ is adapted from a tertiary semi-immiscible system described by Kim and Lowengrub (2005), where *A*_1_ – *A*_5_ are constants. The dimensionless elastic strain energy term $\tilde{W}$ used in Equation (2.9), which follows the form given by Leo et al. (1998) and Garcke (2005), is computed from the elements given below.

#### Elastic energy

(2.10) $$\begin{array}{lll} {\tilde{𝕋}}_{mn} & = & 2\;{\tilde{L}}_{2}\;{\left({\tilde{E}}_{T}\right)}_{mn}+{\tilde{L}}_{1}\delta_{mn}\overset{3}{\underset{s=1}{\sum }}{\left({\tilde{E}}_{T}\right)}_{ss} \end{array}$$

(2.11) $$\begin{array}{lll} {\left({\tilde{E}}_{T}\right)}_{mn} & = & {\tilde{E}}_{mn}-{\tilde{E}}_{mn}^{{\;}^{*}} \end{array}$$

(2.12) $$\begin{array}{lll} {\left({\tilde{E}}_{T}^{{\;}^{*}}\right)}_{mn} & = & {\left({\tilde{E}}_{E}^{{\;}^{*}}\right)}_{mn}-{\left({\tilde{E}}_{C}^{{\;}^{*}}\right)}_{mn} \end{array}$$

(2.13) $$\begin{array}{lll} {\tilde{E}}_{mn}^{{\;}^{*}} & = & Q_{3}\left({\tilde{\phi }}_{E}\right){\left({\tilde{E}}_{T}^{{\;}^{*}}\right)}_{mn}+{\left({\tilde{E}}_{C}^{{\;}^{*}}\right)}_{mn} \end{array}$$

(2.14) $$\begin{array}{l} {\tilde{𝕋}}_{mn}^{{\;}^{*}}=2\;\left(1-{\tilde{L}}_{2}^{C}\right)\;{\left({\tilde{E}}_{T}\right)}_{mn}\\ \;+\left({\tilde{L}}_{1}^{E}-{\tilde{L}}_{1}^{C}\right)\;\delta_{mn}\overset{3}{\underset{k=1}{\sum }}{\left({\tilde{E}}_{T}\right)}_{kk} \end{array}$$

(2.15) $$\begin{array}{lll} {\tilde{E}}_{mn} & = & \frac{1}{2}\left(\frac{\partial {ũ}_{m}}{\partial {\tilde{x}}_{n}}+\frac{\partial {ũ}_{n}}{\partial {\tilde{x}}_{m}}\right) \end{array}$$

(2.16) $$\begin{array}{lll} {\tilde{L}}_{i} & = & Q_{3}\left({\tilde{\phi }}_{E}\right)\left({\tilde{L}}_{i}^{E}-{\tilde{L}}_{i}^{C}\right)+{\tilde{L}}_{i}^{C}\;where\;i=1,\;2 \end{array}$$

where $\tilde{E}$ is the infinitesimal strain, ${\tilde{\text{u}}}_{\text{d}}$ is the displacement vector, ${\tilde{E}}_{\text{E}}^{*}$ and ${\tilde{E}}_{\text{C}}^{*}$ are the Eigenstrain tensors for ECM and cells, respectively. In Equations (2.10) and (2.14), δ_*mn*_ = 1 for *m* = *n* and δ_*mn*_ = 0 for *m*≠*n*. ${\tilde{L}}_{1}^{E}$, ${\tilde{L}}_{2}^{E}$, ${\tilde{L}}_{1}^{C}$, and ${\tilde{L}}_{2}^{C}$ are Lamé constants for the ECM and cell components. The displacement vector ${\tilde{\text{u}}}_{\text{d}}$ is solved by setting $\nabla \cdot {\tilde{𝕋}}_{i}=0$, where ${\tilde{𝕋}}_{i}=\begin{array}{c} \left[{\tilde{𝕋}}_{1i}\;{\tilde{𝕋}}_{2i}\;{\tilde{𝕋}}_{3i}\right] \end{array}$ and *i* = 1, 2, 3.

The dimensionless cell-ECM phase pressure $\tilde{p}$ and interstitial fluid phase pressure $\tilde{q}$, with their corresponding velocities ${\tilde{\text{u}}}_{\alpha }$ and ${\tilde{\text{u}}}_{\beta }$ are computed using the following equations:

#### Pressures and velocities

(2.17) $$\begin{array}{l} \nabla \cdot \left[{\tilde{k}}_{\alpha }\left(\nabla \tilde{p}-\frac{{\tilde{\gamma }}_{T}}{{\tilde{\epsilon }}_{T}}{\tilde{\mu }}_{T}\nabla {\tilde{\phi }}_{T}-\frac{{\tilde{\gamma }}_{E}}{{\tilde{\epsilon }}_{E}}{\tilde{\mu }}_{E}\nabla {\tilde{\phi }}_{E}\right)\right]\\ \;=-\left({\tilde{S}}_{V}+{\tilde{S}}_{D}+{\tilde{S}}_{E}\right) \end{array}$$

(2.18) $$\begin{array}{l} \nabla^{2}\tilde{q}=\frac{R_{\alpha ,\beta }}{{\tilde{k}}_{\beta }}\left({\tilde{S}}_{V}+{\tilde{S}}_{D}+{\tilde{S}}_{E}\right) \end{array}$$

(2.19) $$\begin{array}{l} {\tilde{\text{u}}}_{\alpha }=-{\tilde{k}}_{\alpha }\left[\nabla \tilde{p}-\frac{{\tilde{\gamma }}_{T}}{{\tilde{\epsilon }}_{T}}{\tilde{\mu }}_{T}\nabla {\tilde{\phi }}_{T}-\frac{{\tilde{\gamma }}_{E}}{{\tilde{\epsilon }}_{E}}{\tilde{\mu }}_{E}\nabla {\tilde{\phi }}_{E}\right] \end{array}$$

(2.20) $$\begin{array}{l} {\tilde{\text{u}}}_{\beta }=-{\tilde{k}}_{\beta }\nabla \tilde{q} \end{array}$$

(2.21) $$\begin{array}{l} {\tilde{\text{u}}}_{E}={\tilde{\text{u}}}_{\alpha }-\tilde{M}\nabla \left({\tilde{\mu }}_{E}\right) \end{array}$$

where ${\tilde{k}}_{\alpha }=f\left({\tilde{\phi }}_{T},{\tilde{\phi }}_{E}\right)$ and ${\tilde{k}}_{\beta }$ are motilities of the solid and liquid phase respectively, and ${\tilde{\text{u}}}_{E}$ is the ECM component velocity.

Dimensionless nutrients and waste products concentrations ñ, $\tilde{g}$, $\tilde{w}$, $\tilde{↕}$, $\tilde{b}$, ã, $\tilde{s}$, and $\tilde{r}$ represent O_2_, glucose, CO_2_, lactate, bicarbonate, H^+^, Na^+^, and Cl^−^ respectively. We use the following quasi-steady state governing equations for tracking nutrients and waste products within the tissue domain:

#### Nutrients and waste products

(2.22) $$\begin{array}{l} \nabla \cdot \left({\tilde{D}}_{n}\;\nabla \;ñ\right)+{\tilde{k}}_{n1}{ñ}_{C}\;-\left({\tilde{k}}_{n1}+{\tilde{k}}_{n2}\right)ñ=0 \end{array}$$

(2.23) $$\begin{array}{l} \nabla \cdot \left({\tilde{D}}_{g}\;\nabla \;\tilde{g}\right)+{\tilde{k}}_{g1}{\tilde{g}}_{C}\;-\left({\tilde{k}}_{g1}+{\tilde{k}}_{g2}\right)\tilde{g}=0 \end{array}$$

(2.24) $$\begin{array}{l} \nabla \cdot \left({\tilde{D}}_{w}\;\nabla \;\tilde{w}\right)+{\tilde{k}}_{n2}ñ\;+{\tilde{k}}_{r}\tilde{b}ã+{\tilde{k}}_{w}{\tilde{w}}_{C}-\left({\tilde{k}}_{f}+{\tilde{k}}_{w}\right)\tilde{w}=0 \end{array}$$

(2.25) $$\begin{array}{l} \nabla \cdot \{{\tilde{D}}_{\ell }[\nabla \tilde{\ell }\\ \;-{\tilde{z}}_{\ell }\;\tilde{\ell }\left(\frac{{\tilde{z}}_{\ell }{\tilde{D}}_{\ell }\nabla \tilde{\ell }+{\tilde{z}}_{b}{\tilde{D}}_{b}\nabla \tilde{b}+{\tilde{D}}_{a}\nabla \tilde{a}+{\tilde{z}}_{s}{\tilde{D}}_{s}\nabla \tilde{s}+{\tilde{z}}_{r}{\tilde{D}}_{r}\nabla \tilde{r}}{{\tilde{z}}_{\ell }^{2}\;{\tilde{D}}_{\ell }\;\tilde{\ell }+{\tilde{z}}_{b}^{2}\;{\tilde{D}}_{b}\;\tilde{b}+{\tilde{D}}_{a}\;\tilde{a}+{\tilde{z}}_{s}^{2}\;{\tilde{D}}_{s}\;\tilde{s}+{\tilde{z}}_{r}^{2}\;{\tilde{D}}_{r}\;\tilde{r}}\right)]\}\\ \;+\;2\;R_{g,n}\left({\tilde{k}}_{g2}\tilde{g}\right)-\frac{1}{3}\left({\tilde{k}}_{n2}\tilde{n}\right)+\;{\tilde{k}}_{\ell }{\tilde{\ell }}_{C}-\;{\tilde{k}}_{\ell }\tilde{\ell }=0 \end{array}$$

(2.26) $$\begin{array}{l} \nabla \cdot \{{\tilde{D}}_{b}[\nabla \tilde{b}\\ \;-{\tilde{z}}_{b}\;\tilde{b}\left(\frac{{\tilde{z}}_{\ell }{\tilde{D}}_{\ell }\nabla \tilde{\ell }+{\tilde{z}}_{b}{\tilde{D}}_{b}\nabla \tilde{b}+{\tilde{D}}_{a}\nabla \tilde{a}+{\tilde{z}}_{s}{\tilde{D}}_{s}\nabla \tilde{s}+{\tilde{z}}_{r}{\tilde{D}}_{r}\nabla \tilde{r}}{{\tilde{z}}_{\ell }^{2}\;{\tilde{D}}_{\ell }\;\tilde{\ell }+{\tilde{z}}_{b}^{2}\;{\tilde{D}}_{b}\;\tilde{b}+{\tilde{D}}_{a}\;\tilde{a}+{\tilde{z}}_{s}^{2}\;{\tilde{D}}_{s}\;\tilde{s}+{\tilde{z}}_{r}^{2}\;{\tilde{D}}_{r}\;\tilde{r}}\right)]\}\\ \;+\;{\tilde{k}}_{f}\tilde{w}-{\tilde{k}}_{r}\tilde{b}\tilde{a}=0 \end{array}$$

(2.27) $$\begin{array}{l} \nabla \cdot \{{\tilde{D}}_{a}[\nabla \tilde{a}\\ \;-{\tilde{z}}_{a}\;\tilde{a}\left(\frac{{\tilde{z}}_{\ell }{\tilde{D}}_{\ell }\nabla \tilde{\ell }+{\tilde{z}}_{b}{\tilde{D}}_{b}\nabla \tilde{b}+{\tilde{D}}_{a}\nabla \tilde{a}+{\tilde{z}}_{s}{\tilde{D}}_{s}\nabla \tilde{s}+{\tilde{z}}_{r}{\tilde{D}}_{r}\nabla \tilde{r}}{{\tilde{z}}_{\ell }^{2}\;{\tilde{D}}_{\ell }\;\tilde{\ell }+{\tilde{z}}_{b}^{2}\;{\tilde{D}}_{b}\;\tilde{b}+{\tilde{D}}_{a}\;\tilde{a}+{\tilde{z}}_{s}^{2}\;{\tilde{D}}_{s}\;\tilde{s}+{\tilde{z}}_{r}^{2}\;{\tilde{D}}_{r}\;\tilde{r}}\right)]\}\\ \;+2\;R_{g,n}\left({\tilde{k}}_{g2}\tilde{g}\right)\text{​}-\text{​}\frac{1}{3}\left({\tilde{k}}_{n2}\tilde{n}\right)\text{​}+\text{​}{\tilde{k}}_{f}\tilde{w}\text{​}-\text{​}{\tilde{k}}_{r}\tilde{b}\tilde{a}\text{​}+\text{​ }{\tilde{k}}_{\ell }{\tilde{\ell }}_{C}\text{​}-\text{​ }{\tilde{k}}_{\ell }\tilde{\ell }\text{​}=\text{​}0 \end{array}$$

(2.28) $$\begin{array}{l} \nabla \cdot \{{\tilde{D}}_{s}[\nabla \tilde{s}\\ \;-{\tilde{z}}_{s}\;\tilde{s}\left(\frac{{\tilde{z}}_{\ell }{\tilde{D}}_{\ell }\nabla \tilde{\ell }+{\tilde{z}}_{b}{\tilde{D}}_{b}\nabla \tilde{b}+{\tilde{D}}_{a}\nabla \tilde{a}+{\tilde{z}}_{s}{\tilde{D}}_{s}\nabla \tilde{s}+{\tilde{z}}_{r}{\tilde{D}}_{r}\nabla \tilde{r}}{{\tilde{z}}_{\ell }^{2}\;{\tilde{D}}_{\ell }\;\tilde{\ell }+{\tilde{z}}_{b}^{2}\;{\tilde{D}}_{b}\;\tilde{b}+{\tilde{D}}_{a}\;\tilde{a}+{\tilde{z}}_{s}^{2}\;{\tilde{D}}_{s}\;\tilde{s}+{\tilde{z}}_{r}^{2}\;{\tilde{D}}_{r}\;\tilde{r}}\right)]\}\\ \;=0 \end{array}$$

(2.29) $$\begin{array}{l} \tilde{r}=-\frac{1}{{\tilde{z}}_{r}}\left({\tilde{z}}_{\ell }\tilde{\ell }+{\tilde{z}}_{b}\tilde{b}+{\tilde{z}}_{a}ã+{\tilde{z}}_{s}\tilde{s}\right) \end{array}$$

The flux terms of charged species follow those given by Casciari et al. (1992). The terms ${\tilde{k}}_{n1}$, ${\tilde{k}}_{n2}$, ${\tilde{k}}_{g1}$, ${\tilde{k}}_{g2}$, ${\tilde{k}}_{w}$, and ${\tilde{k}}_{↕}$ are combined rate constants used in source terms of nutrients and waste products, whereas ${\tilde{k}}_{f}$ and ${\tilde{k}}_{r}$ are the forward and reverse rate constants of the reaction $CO_{2}+H_{2}O↔HCO_{3}^{-}+H^{+}$.

Dimensionless concentrations of tumor growth factors, tumor angiogenic factors, matrix degrading enzymes, and myofibroblastic cells are represented by $t\tilde{g}f$, $t\tilde{a}f$, $\tilde{m}$, and ${\tilde{F}}_{E}$ respectively. Quasi-steady state equations for $t\tilde{g}f$ and $t\tilde{a}f$, as well as transient governing equations for $\tilde{m}$ and ${\tilde{F}}_{E}$ are given below:

#### Tumorigenic species

(2.30) $$\begin{array}{l} \nabla \cdot \left({\tilde{D}}_{tgf}\nabla t\tilde{g}f\right)+{\tilde{\lambda }}_{tgf}\\ \;-\left({\tilde{\lambda }}_{tgf}+{\tilde{\lambda }}_{de,tgf}+{\tilde{\lambda }}_{U,tgf}\right)t\tilde{g}f=0 \end{array}$$

(2.31) $$\begin{array}{l} \nabla \cdot \left({\tilde{D}}_{taf}\nabla t\tilde{a}f\right)+{\tilde{\lambda }}_{taf}\\ \;-\left({\tilde{\lambda }}_{taf}+{\tilde{\lambda }}_{de,taf}+{\tilde{\lambda }}_{U,taf}\right)t\tilde{a}f=0 \end{array}$$

(2.32) $$\begin{array}{l} \frac{\partial \tilde{m}}{\partial \tilde{t}}=\;\nabla \cdot \left({\tilde{D}}_{m}\nabla \tilde{m}\right)+{\tilde{S}}_{m} \end{array}$$

(2.33) $$\begin{array}{l} \frac{\partial {\tilde{F}}_{E}}{\partial t}+\nabla \cdot \left({\tilde{F}}_{E}{\tilde{\text{u}}}_{E}\right)=-\;\nabla \cdot \left({\tilde{D}}_{F}{\tilde{F}}_{E}\nabla t\tilde{g}f\right)+{\tilde{S}}_{FE} \end{array}$$

Here, ${\tilde{F}}_{E}$ reflects the concentration within the ECM phase, assuming the volume of myofibroblastic cells is negligible and the ECM phase is continuous throughout the domain. Similarly, dimensionless ECM based concentrations of blood and lymphatic vessels, ${\tilde{B}}_{n}^{E}$ and ${\tilde{L}}_{n}^{E}$ respectively, with their corresponding diffusive flux terms are given by.

#### Blood and lymphatic vessels

(2.34) $$\begin{array}{l} \frac{\partial {\tilde{B}}_{n}^{E}}{\partial t}+\nabla \cdot \left({\tilde{B}}_{n}^{E}{\tilde{\text{u}}}_{E}\right)=-\;\nabla \cdot {\tilde{\text{J}}}_{BnE}+{\tilde{S}}_{BnE} \end{array}$$

(2.35) $$\begin{array}{l} \frac{\partial {\tilde{L}}_{n}^{E}}{\partial t}+\nabla \cdot \left({\tilde{L}}_{n}^{E}{\tilde{\text{u}}}_{E}\right)=-\;\nabla \cdot {\tilde{\text{J}}}_{LnE}+{\tilde{S}}_{LnE} \end{array}$$

(2.36) $$\begin{array}{l} {\tilde{J}}_{BnE}={\tilde{\chi }}_{che,BnE}\;A_{che,BnE}\;{\tilde{B}}_{n}^{E}\;\nabla t\tilde{a}f\\ \;+{\tilde{\chi }}_{hap,BnE}\;A_{hap,BnE}\;{\tilde{B}}_{n}^{E}\;\nabla {\tilde{\phi }}_{E}-{\tilde{D}}_{BnE}\nabla {\tilde{B}}_{n}^{E} \end{array}$$

(2.37) $$\begin{array}{l} {\tilde{\text{J}}}_{LnE}={\tilde{\chi }}_{che,LnE}\;A_{che,LnE}\;{\tilde{L}}_{n}^{E}\;\nabla t\tilde{a}f\\ \;+{\tilde{\chi }}_{hap,LnE}\;A_{hap,LnE}\;{\tilde{L}}_{n}^{E}\;\nabla {\tilde{\phi }}_{E}-{\tilde{D}}_{LnE}\nabla {\tilde{L}}_{n}^{E} \end{array}$$

Tissue effective diffusivities ${\tilde{D}}_{n}$, ${\tilde{D}}_{g}$, ${\tilde{D}}_{w}$, ${\tilde{D}}_{↕}$, ${\tilde{D}}_{b}$, ${\tilde{D}}_{a}$, ${\tilde{D}}_{s}$, ${\tilde{D}}_{tgf}$, ${\tilde{D}}_{taf}$, ${\tilde{D}}_{m}$, ${\tilde{D}}_{FE}$, ${\tilde{D}}_{BnE}$, ${\tilde{D}}_{LnE}$, and tissue effective mass transfer coefficients ${\tilde{\lambda }}_{B,n}$, ${\tilde{\lambda }}_{B,g}$, ${\tilde{\lambda }}_{B,w}$, ${\tilde{\lambda }}_{B,l}$, are represented by $\tilde{\psi }$ and determined as follow:

(2.38) $$\begin{array}{l} \tilde{\psi }=\;{\tilde{\psi }}_{E}\;Q_{3}\left({\tilde{\phi }}_{E}\right)\\ \;+\left[1-Q_{3}\left({\tilde{\phi }}_{E}\right)\right]\left\{{\tilde{\psi }}_{T}\;Q_{3}\left(\frac{{\tilde{\phi }}_{T}}{{\tilde{\phi }}_{C}}\right)+{\tilde{\psi }}_{H}\left[1-Q_{3}\left(\frac{{\tilde{\phi }}_{T}}{{\tilde{\phi }}_{C}}\right)\right]\right\} \end{array}$$

where ${\tilde{\psi }}_{E}$, ${\tilde{\psi }}_{T}$, and ${\tilde{\psi }}_{H}$ are dimensionless diffusivity or transfer coefficient in the ECM, tumor, and healthy-host cell domain respectively. The total cell volume fraction is ${\tilde{\phi }}_{C}={\tilde{\phi }}_{T}+{\tilde{\phi }}_{H}$. The full list of parameters, constants, factors used in non-dimensionalization, source terms, rate terms, and adjustment factors can be found in Supplementary Tables 1–6.

We define the following Neumann boundary conditions for the cell and ECM volume fractions, and Dirichlet boundary conditions for solid cell pressure and chemical potentials at all external boundaries:

(2.39) $$\begin{array}{lll} n\cdot \nabla {\tilde{\phi }}_{\text{V}} & = & n\cdot \nabla {\tilde{\phi }}_{\text{D}}=n\cdot \nabla {\tilde{\phi }}_{\text{E}}=0,\\ \tilde{p} & = & {\tilde{\mu }}_{T}={\tilde{\mu }}_{E}={\tilde{\mu }}_{H}=0 \end{array}$$

where ***n*** is the outward normal of a boundary. For nutrients and waste products, as well as tumorigenic species, Dirichlet boundary conditions are imposed, with the exception of myofibroblastic cell species, where Neumann boundary conditions are applied:

(2.40) $$\begin{array}{lll} \tilde{n} & = & \tilde{g}\;=1,\\ \tilde{w} & = & \tilde{\ell }\;=\;\tilde{b}\;=\;\tilde{a}\;=\;\tilde{s}\;=\;t\tilde{g}f\;=\;t\tilde{a}f\;=\;\tilde{m}\;=\;0,\\ n\cdot \nabla {\tilde{F}}_{E} & = & 0. \end{array}$$

Blood and lymphatic vessels are assumed to be at their corresponding far-field values at external boundaries:

(2.41) $$\begin{array}{l} {\tilde{B}}_{n}^{E}={\tilde{B}}_{\infty }\;,\;{\tilde{L}}_{n}^{E}={\tilde{L}}_{\infty }\;, \end{array}$$

where ${\tilde{B}}_{\infty }={\tilde{L}}_{\infty }=0.2$ is used here.

### Numerical methods

The mathematical model is first discretized in time and space. After reorganizing the discretized equations, a non-linear relaxation procedure is employed.

#### Computational domain

We consider a rectangular 3D domain Ω = (*L*_*x*_, *R*_*x*_) × (*L*_*y*_, *R*_*y*_) × (*L*_*z*_, *R*_*z*_). Let the domain be discretized into *N*_*x*_ × *N*_*y*_ × *N*_*z*_ cells. Bounded by boundary cells, the domain is covered by the following sets of cell centers, cell edge points, and cell grid points:

(2.2.1) $$\begin{array}{lll} C & = & \left\{\left(x_{i},y_{j},z_{k}\right)|\;0\leq i\leq N_{x}+1,\;0\leq j\leq N_{y}+1,\;0\leq k\leq N_{z}+1\right\} \end{array}$$

(2.2.2) $$\begin{array}{lll} E_{ew} & = & \left\{\left(x_{i+\frac{1}{2}},y_{j},z_{k}\right)|\;0\leq i\leq N_{x},\;1\leq j\leq N_{y},\;1\leq k\leq N_{z}\right\}, \end{array}$$

(2.2.3) $$\begin{array}{lll} E_{ns} & = & \left\{\left(x_{i},y_{j+\frac{1}{2}},z_{k}\right)|1\leq i\leq N_{x},\;0\leq j\leq N_{y},\;1\leq k\leq N_{z},\right\}, \end{array}$$

(2.2.4) $$\begin{array}{lll} E_{tb} & = & \left\{\left(x_{i},y_{j},z_{k+\frac{1}{2}}\right)|1\leq i\leq N_{x},\;1\leq j\leq N_{y},\;0\leq k\leq N_{z},\right\}, \end{array}$$

(2.2.5) $$\begin{array}{lll} G & = & \left\{\left(x_{i+\frac{1}{2}},y_{j+\frac{1}{2}},z_{k+\frac{1}{2}}\right)|\;0\leq i\leq N_{x},\;0\leq j\leq N_{y},\;0\leq k\leq N_{z},\right\} \end{array}$$

where ***E***_***ew***_, ***E***_***ns***_, and ***E***_***tb***_ represent east-west, north-south, and top-bottom sets of cell edges respectively, and ***G*** represents cell corners. Let grid spaces be

(2.2.6) $$\begin{array}{lll} \Delta x_{i} & = & x_{i+\frac{1}{2}}-x_{i-\frac{1}{2}},\\ \Delta y_{j} & = & \Delta y_{j+\frac{1}{2}}-y_{j-\frac{1}{2}},\\ \Delta z_{k} & = & z_{k+\frac{1}{2}}-z_{k-\frac{1}{2}}, \end{array}$$

and if partitions in the three directions are uniform and equal:

(2.2.7) $$\begin{array}{lll} \Delta x_{i} & = & \Delta x_{j}=\Delta x_{k}=\eta \;, \end{array}$$

(2.2.8) $$\begin{array}{lll} \eta & = & \frac{R_{x}-L_{x}}{N_{x}}=\frac{R_{y}-L_{y}}{N_{y}}=\frac{R_{z}-L_{z}}{N_{z}}\;, \end{array}$$

for 1 ≤ *i* ≤ *N*_*x*_, 1 ≤ *j* ≤ *N*_*y*_, and 1 ≤ *k* ≤ *N*_*z*_, we get the following coordinates for cell centers and cell edges or corners defined in Equations (2.2.1)–(2.2.5):

(2.2.9) $$\begin{array}{lll} x_{i} & = & L_{x}+\left(i-\frac{1}{2}\right)\eta \;,y_{j}=L_{y}+\left(j-\frac{1}{2}\right)\eta \;,\\ z_{k} & = & L_{z}+\left(k-\frac{1}{2}\right)\eta \;, \end{array}$$

(2.2.10) $$\begin{array}{lll} x_{i+\frac{1}{2}} & = & L_{x}+i\;\eta \;,y_{j+\frac{1}{2}}=L_{y}+j\;\eta \;,\\ z_{k+\frac{1}{2}} & = & L_{z}+k\;\eta \;, \end{array}$$

following their corresponding ranges indicated in Equations (2.2.1)–(2.2.5).

The differential, Laplacian and flux terms associated with the model are in Supplementary Materials.

#### Model discretization

The model consists of a set of stiff differential equations that are fourth-order in space. At time step *a* with time step size θ, they are discretized in time using the Crank-Nicolson Method as in Wise et al. (2011). Details of the discretization as well as the multigrid V-cycle iterations and non-linear Gauss–Seidel relaxations are given in Supplementary Materials.

The tumor model solved using the adaptive full multigrid V-cycle was coded in C and simulations were performed on a node equipped with 768 GB of RAM and 32 Intel Xeon 3.3 GHz cores running CentOS 6.7 x86_64. The algorithms were partially parallelized using OpenMP to achieve higher performance.

## Results

### Self-adaptive full multigrid full approximation scheme V-cycle algorithm

The self-adaptive full multigrid process combines FMG with the self-adaptive approach. The process involves constructing a multilevel block-structured mesh and seeking the solution on the new mesh structure. Mesh construction and refinement, as well as FAS multigrid in an adaptive FMG setting were developed as follows.

#### Multilevel mesh refinement

**Figure 1:**
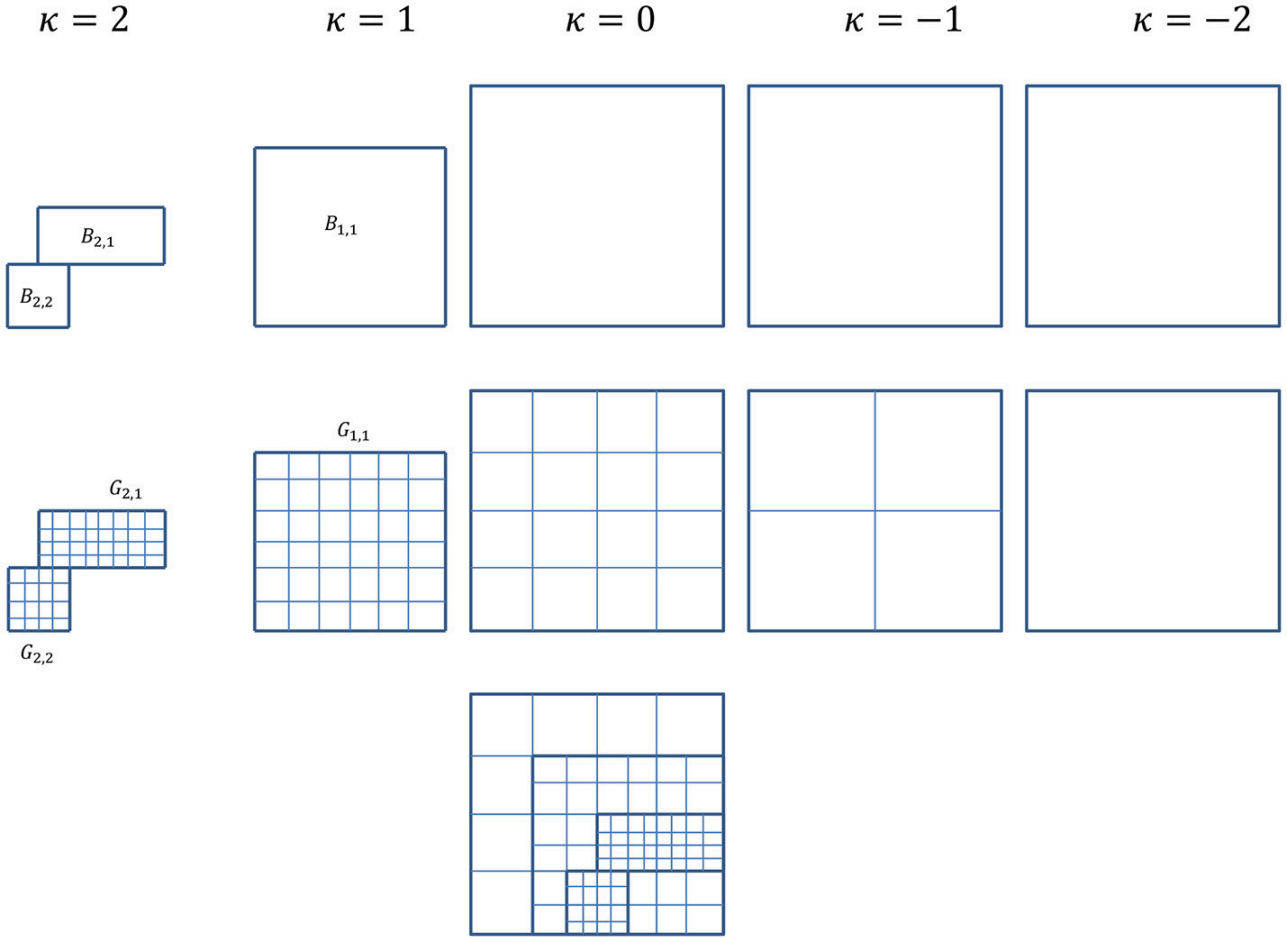
For simplification and ease of visualization, a two dimensional example is given here. The multigrid algorithm consists of a hierarchy of grid levels κ = −2, …, 2, shown here on the top rowwith their corresponding subdomains/blocks. Level κ = 0 is the root level and levels κ = 1 and 2 are refinement levels. Global levels κ = 0, −1, and −2 cover the entire computational domain, whereas refinement levels κ = 1 and 2 cover subdomains where refinement is needed. Shown in the example here, the locally refined block-structured κ = 1 consists of one block, *B*_1, 1_, and κ = 2 consists of two blocks, *B*_2, 1_ and *B*_2, 2_. The middle row shows the corresponding meshes for each level. For refinement levels in the example here, mesh *G*_1, 1_ for block *B*_1, 1_, *G*_2, 1_ and *G*_2, 2_ for blocks *B*_2, 1_ and *B*_2, 2_. The composite grids of all levels is shown in the last row.

We start with a rectangular computational domain as described in **Methods**. Each level κ covers a domain of Ω_κ_ with mesh size η_κ_. Grid levels are numbered as κ = κ_min_, …, 0, …, κ_max_, where κ_min_ represents the coarsest mesh level and κ_max_ the finest. Global grid levels, κ = 0 − κ_min_, cover the entire computational domain Ω_κ_ = Ω. The finest global grid level, κ = 0, is referred as the root level. Grid levels κ = 1 to κ = κ_max_ are levels with refined mesh covering a domain of Ω_κ+1_ ⊆ Ω_κ_ ⊆ Ω, each consists of *n*_κ_ rectangular blocks, *B*_κ, 1_, …, *B*_κ,_*n*__κ__, of uniform grids, *G*_κ, 1_, …, *G*_κ,_*n*__κ__. Note that *n*_κ_ = 1 for all global grid levels. Cell-centered discretization is used with grid spacing $\eta_{\kappa +1}=\frac{\eta_{\kappa }}{2}$. The hierarchy of levels and meshes are shown in Figure 1.

After solution is obtained for the current finest level, we use the undivided gradient test (Wise et al., 2007) to tag cells for refinement. The undivided gradient test is used to capture the diffuse interface region (tumor-host cell domain). Let the critical value of some indicative variable ψ_κ_(*x, y, z*) be ***C***_κ_, cell-centered coordinates on level κ be ***x***_*i, j, k*_, the following test is performed on cells on level κ:

(3.1.1) $$\begin{array}{l} F_{\kappa }=\begin{array}{c} \left\{x_{i,j,k}\in \Omega_{\kappa }|\sqrt{\begin{array}{c} {\left[\psi_{i+1,j,k}-\psi_{i-1,j,k}\right]}^{2}\\ +{\left[\psi_{i,j+1,k}-\psi_{i,j-1,k}\right]}^{2}\\ +{\left[\psi_{i,j,k+1}-\psi_{i,j,k-1}\right]}^{2} \end{array}}>C_{\kappa }\right\} \end{array} \end{array}$$

where ***F***_κ_ are coordinates of flagged cells. Another criteria that can be used to tag cells for refinement is the relative truncation error test (Trottenberg et al., 2001). In the relative truncation error test, the relative truncation error with respect to Ω_κ_ and Ω_κ−1_,

(3.1.2) $$\begin{array}{l} \tau_{h}^{2h}=L_{\kappa -1}\left(R_{\kappa }^{\kappa -1}\psi_{\kappa }^{a,r,\nu_{1}}\right)-R_{\kappa }^{\kappa -1}L_{\kappa }\left(\psi_{\kappa }^{a,r,\nu_{1}}\right) \end{array}$$

as used in Equation (3.1.20) in the FAS cycle, is used to flag cells for refinement. A subroutine is created and called to perform this task:

(3.1.3) $$\begin{array}{l} F_{\kappa }^{a,n}=\text{FLAG}\left(\;\psi_{\kappa }^{a,r}\right) \end{array}$$

where the current finest-grid data is used and $F_{\kappa }^{a,n}$ is a list of flagged coordinates.

The flagged cells are then rearranged into patches of rectangular refined mesh in the next finer level using a clustering algorithm from Bell et al. (1994) and Berger and Rigoutsos (1991) with minor modification. Create a list of blocks and start with just one block containing all flagged cells given by $F_{\kappa }^{a,n}$. The following procedure is used to divide flagged cells into blocks:

1. Compute efficiency of the block, which is the ratio of flagged cells to total cells. If the efficiency is below the threshold efficiency, and, if the size of the block is more than twice the threshold size, then continue. Else, accept and add the current block to the list, return to Step (1) for the next block on the list.
2. Compute signatures, which is the number of flagged cells in each slice along each direction. Checking for gaps along all dimensions starting from the longest edge, find a gap closest to the center of that direction. If gaps exist and an optimum gap location is found, slice the block into two along the gap, and go to Step (5). If no gaps are found, continue.
3. Compute second derivatives of the signatures along all directions. Checking for inflection points starting from the longest edge, find an inflection point closest to the center of that direction. If inflection points are found and an optimum inflection location is found, slice the block into two along the inflection point, and go to Step (5). If no inflection points are found, continue.
4. If the efficiency of the block is above a minimum allowed, accept and add the current block to the list, returning to Step (1) for the next block on the list. Else, divide the block into two along the mid-point of the longest dimension and continue.
5. Delete all empty slices along the edges of each block, and add the two trimmed blocks to the list. Repeat from Step (1) until all blocks created are checked.

The above task is assigned to the following subroutine of block generation:

(3.1.4) $$\begin{array}{l} B_{\kappa +1}=\text{BLOCKGEN}\left(threshold\text{\_}eff,\;threshold\text{\_}size,\;min\text{\_}eff,\;F_{\kappa }^{a,n}\right) \end{array}$$

where ***B***_κ+1_ is an array of blocks on the refined level κ + 1, with rows of coordinates corresponding to corners of each block.

**Figure 2:**
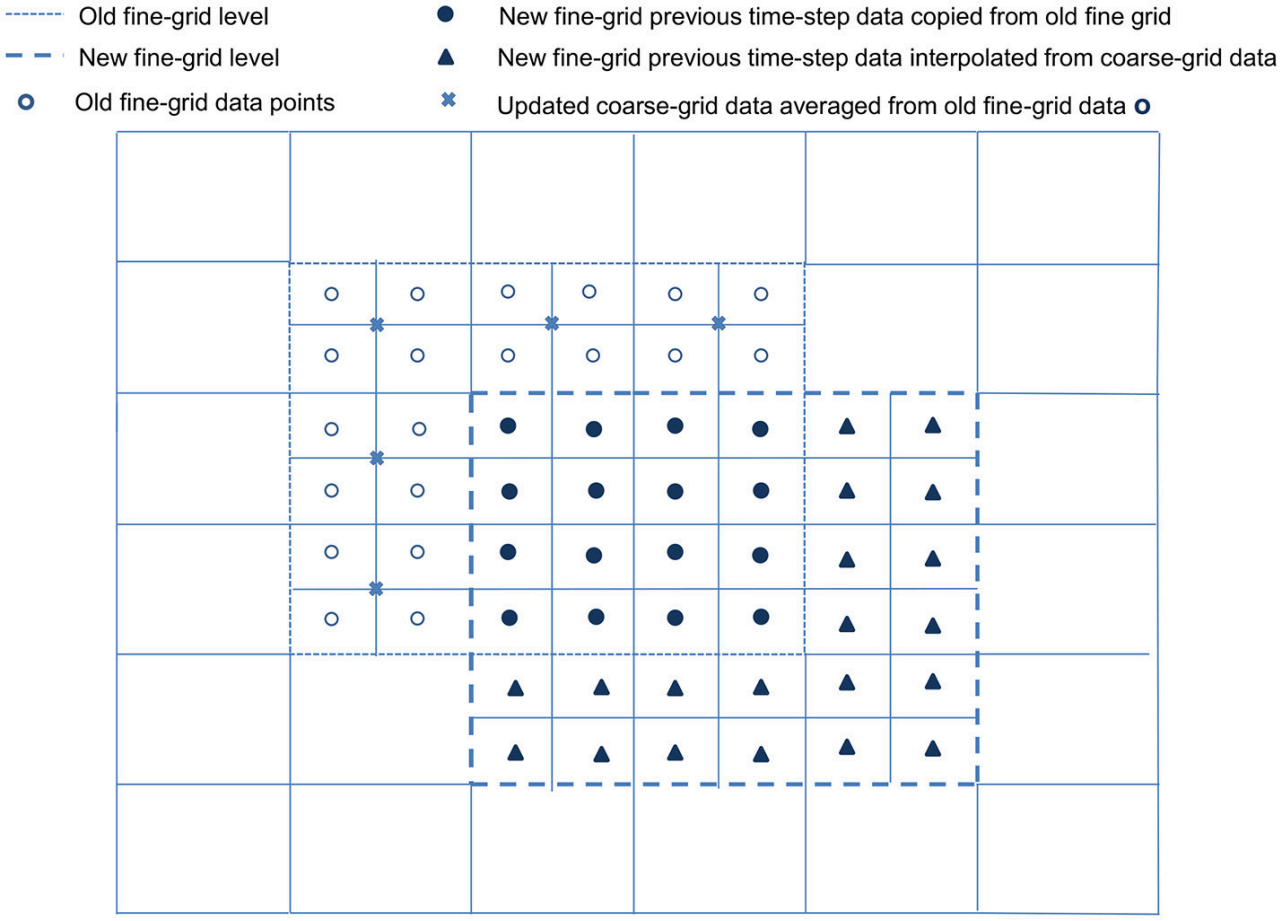
Populating a newly refined grid. For simplification and ease of visualization, a two dimensional example is illustrated here. An existing block, with its boundary marked by - -, is overlapped by a newly refined block with boundary marked by 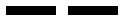. All cell-centered variable data on the newly refined grid are obtained via cubic interpolation from the coarse grid data below. The previous time-step data for the new grid points (•), which overlap old grid points, are copied directly from the old grid points they overlap; for new grid points (▴) that do not overlap with any old grid, their previous time-step data are obtained from the coarse grid below. The remaining data on the old grid (°) that do not overlap any of the new grid points are averaged and copied to the coarse grid data points (×).

Following the generation of new refined grids on level κ + 1, the new grids are populated with data from level κ and the old level κ + 1. All cell-centered data for variables on the newly refined grids must be generated by higher order interpolation, such as cubic interpolation given by Equations (3.1.28) and (3.1.29), from the coarse grids below. If a new level κ + 1 grid cell overlaps any old level κ + 1 grid cell, the previous time-step data for the new grid cell is copied from the old. However, for any new level κ + 1 grid cell that does not overlap with an old κ + 1 grid cell, its previous time-step data has to be obtained from the coarse grid below. For all old level κ + 1 grid cells that do not overlap with any new κ + 1 grid cells, data are averaged and stored in the coarse level κ grid. Refer to Figure 2 for illustration.

**Figure 3:**
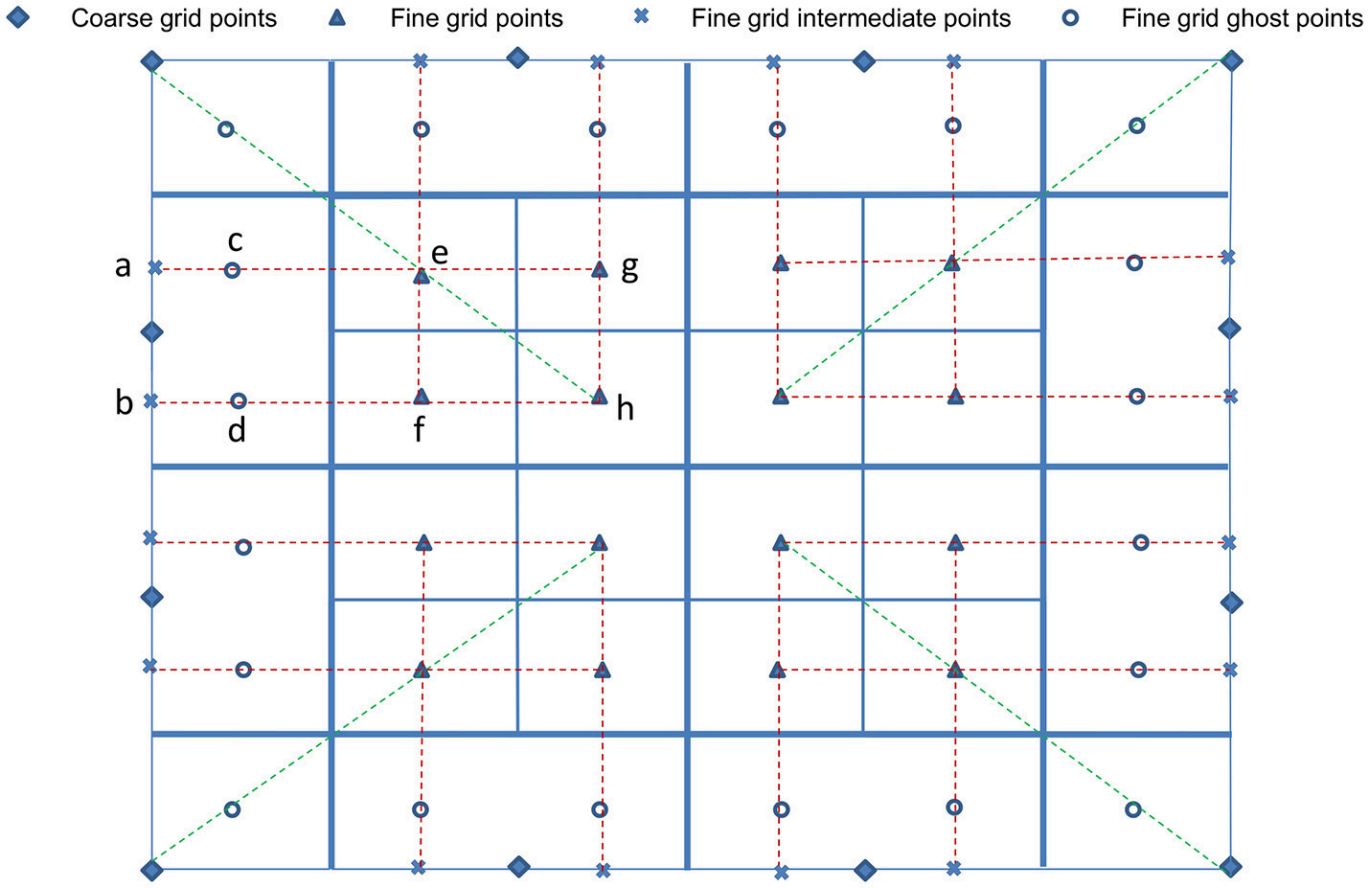
Interpolation at fine-grid ghost layers. For simplification and ease of visualization, a two dimensional illustration is presented. The coarse grid mesh is shown using thick solid lines, and the finer grid mesh is shown using thin solid lines. The internal cell-centered grid points of the finer grid are depicted by (▴) and its external/ghost grid points are represented by (°). Only relevant cell-centered grid points (♦) on the coarse grids are shown. First, data on intermediate points (×) are obtained using quadratic interpolation from their nearest three coarse grid points (♦). Corner ghost cells (°) are obtained by quadratic interpolation using the nearest coarse-grid data (♦) and two fine-grid data (▴) on the diagonal, shown by green dashed lines. Remaining ghost cells (°) are obtained from the intermediate points (×) and two fine-grid interior points (▴), shown by red dashed lines.

After populating the newly refined grid, ghost cells surrounding each grid patch must be constructed. We first compute data for the ghost cells using the Π quadratic interpolation given by Colella et al. (2009). Illustrated in Figure 3, quadratic interpolation is performed twice. First, to get data from the coarse-grid for the intermediate points *a* and *b*:

(3.1.5) $$\begin{array}{ll} I_{\kappa -1}^{\kappa }\psi_{\kappa -1}\left(x,y\right)=\; & \frac{1}{32}[30\psi_{\kappa -1}\left(x,y-\frac{\eta_{\kappa }}{2}\right)\\ \; & +5\psi_{\kappa -1}\left(x,y+\frac{3\eta_{\kappa }}{2}\right)]\;\\ \; & -3\psi_{\kappa -1}\left(x,y-\frac{5\eta_{\kappa }}{2}\right)], \end{array}$$

(3.1.6) $$\begin{array}{ll} I_{\kappa -1}^{\kappa }\;\psi_{\kappa -1}\left(x,y\right)=\; & \frac{1}{32}[30\;\psi_{\kappa -1}\left(x,y+\frac{\eta_{\kappa }}{2}\right)\;\\ +5\;\psi_{\kappa -1}\left(x,y-\frac{3\eta_{\kappa }}{2}\right)]\\ -\;3\;\psi_{\kappa -1}\left(x,y+\frac{5\eta_{\kappa }}{2}\right)]\;, \end{array}$$

respectively. Then, to compute fine-grid boundary data on ghost cell *c*, perform quadratic interpolation using points *a*, *e*, and *g*. Similarly, interpolate using points *b*, *f*, and *h* to obtain data at ghost cell *d*. Following Wise et al. (2007), all ghost cells of each fine-grid patch are first filled using the quadratic interpolation above. If a ghost cell falls on a cell on any neighboring fine-grid patches of the same level, then the ghost cell data is replaced by the more accurate cell-centered data from the cell of the neighboring patch.

#### Adaptive FAS V-cycle

Consider a case with κ grid levels with κ = 0 being the root level. The levels κ = κ_max_ and κ = κ_min_ represent the level with the finest and coarsest grid, respectively. Each level of κ > 0 contains one or more blocks of refined rectangular grids covering a subdomain Ω_κ_, whereas levels κ ≤ 0 cover the entire computational domain Ω_κ_ = Ω.

**Figure 4:**
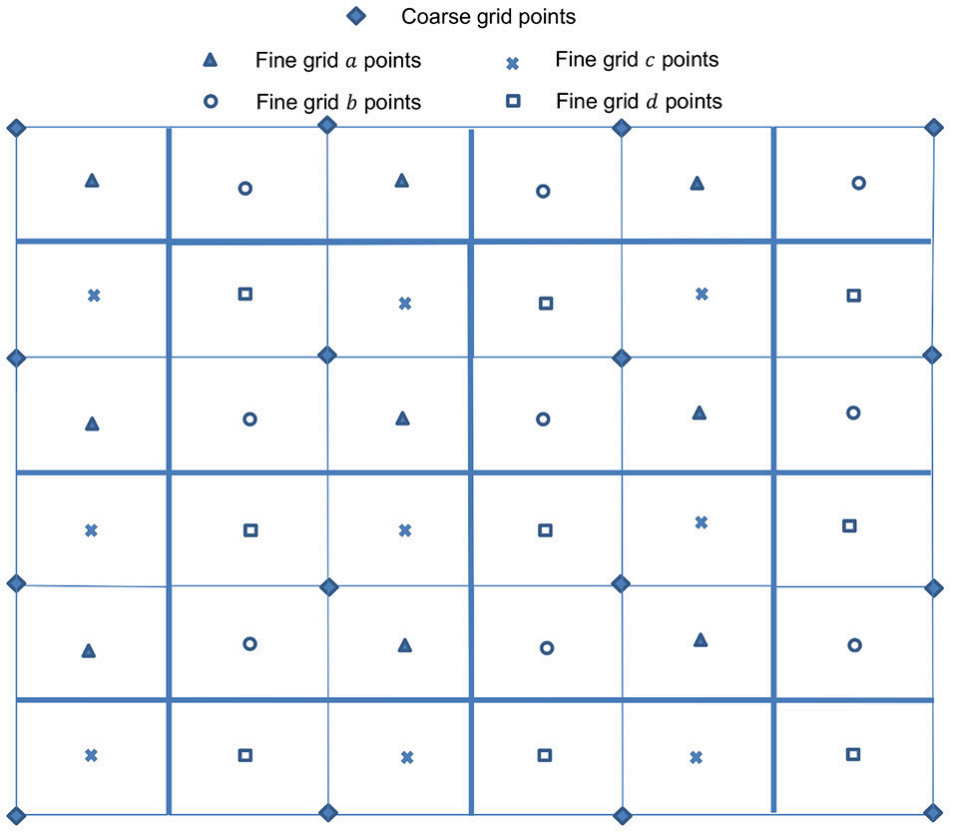
Arrangement of unknowns in 2D cell-centered discretization.

**Figure 5:**
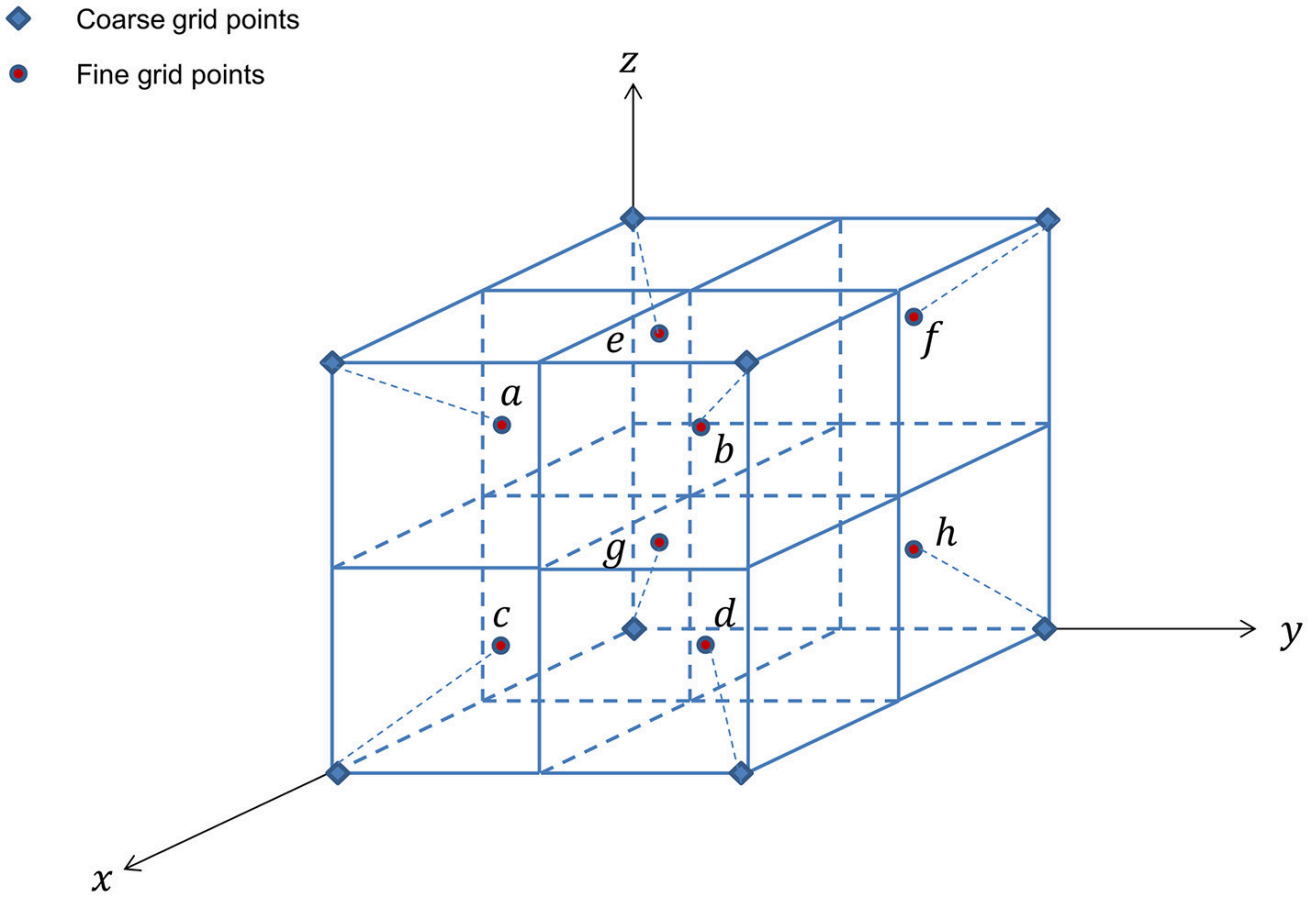
Arrangement of unknowns in 3D cell-centered discretization.

Averaging is used for the restriction operator $R_{\kappa }^{\kappa -1}$:

(3.1.7) $$\begin{array}{ll} R_{\kappa }^{\kappa -1}\psi_{\kappa }\left(x,y,z\right)= & \frac{1}{8}[\psi_{\kappa }\left(x-\frac{\eta_{\kappa }}{2},y-\frac{\eta_{\kappa }}{2},z-\frac{\eta_{\kappa }}{2}\right)\\ +\psi_{\kappa }\left(x-\frac{\eta_{\kappa }}{2},y+\frac{\eta_{\kappa }}{2},z-\frac{\eta_{\kappa }}{2}\right)\;\\ +\;\psi_{\kappa }\left(x+\frac{\eta_{\kappa }}{2},y-\frac{\eta_{\kappa }}{2},z-\frac{\eta_{\kappa }}{2}\right)\\ +\psi_{\kappa }\left(x+\frac{\eta_{\kappa }}{2},y+\frac{\eta_{\kappa }}{2},z-\frac{\eta_{\kappa }}{2}\right)\\ +\;\psi_{\kappa }\left(x-\frac{\eta_{\kappa }}{2},y-\frac{\eta_{\kappa }}{2},z+\frac{\eta_{\kappa }}{2}\right)\\ +\psi_{\kappa }\left(x-\frac{\eta_{\kappa }}{2},y+\frac{\eta_{\kappa }}{2},z+\frac{\eta_{\kappa }}{2}\right)\\ +\;\psi_{\kappa }\left(x+\frac{\eta_{\kappa }}{2},y-\frac{\eta_{\kappa }}{2},z+\frac{\eta_{\kappa }}{2}\right)\\ +\psi_{\kappa }\left(x\text{​}+\text{​}\frac{\eta_{\kappa }}{2},y\text{​}+\text{​}\frac{\eta_{\kappa }}{2},z\text{​}+\text{​}\frac{\eta_{\kappa }}{2}\right)] \end{array}$$

which reduces to a four-point average in a 2D case. Linear interpolation is used for the prolongation operators $P_{\kappa -1}^{\kappa }$ in error correction steps within a V-cycle. In the 2D cell-centered discretization cases as depicted in Figure 4, the bilinear interpolation operators are given by Trottenberg et al. (2001):

(3.1.8) $$\begin{array}{ll} P_{\kappa -1}^{\kappa }\psi_{\kappa -1}\left(x,y\right)= & \frac{1}{16}[9\psi_{\kappa -1}\left(x-\frac{\eta_{\kappa }}{2},y+\frac{\eta_{\kappa }}{2}\right)\\ +3\;\psi_{\kappa -1}\left(x-\frac{\eta_{\kappa }}{2},y-\frac{3\eta_{\kappa }}{2}\right)\\ +\;3\psi_{\kappa -1}\left(x+\frac{3\eta_{\kappa }}{2},y+\frac{\eta_{\kappa }}{2}\right)\\ +\psi_{\kappa -1}\left(x+\frac{3\eta_{\kappa }}{2},y-\frac{3\eta_{\kappa }}{2}\right)] \end{array}$$

(3.1.9) $$\begin{array}{ll} P_{\kappa -1}^{\kappa }\psi_{\kappa -1}\left(x,y\right)= & \frac{1}{16}[9\psi_{\kappa -1}\left(x+\frac{\eta_{\kappa }}{2},y+\frac{\eta_{\kappa }}{2}\right)\\ +3\;\psi_{\kappa -1}\left(x+\frac{\eta_{\kappa }}{2},y-\frac{3\eta_{\kappa }}{2}\right)\\ +\;3\psi_{\kappa -1}\left(x-\frac{3\eta_{\kappa }}{2},y+\frac{\eta_{\kappa }}{2}\right)\\ +\psi_{\kappa -1}\left(x-\frac{3\eta_{\kappa }}{2},y-\frac{3\eta_{\kappa }}{2}\right)] \end{array}$$

(3.1.10) $$\begin{array}{ll} P_{\kappa -1}^{\kappa }\psi_{\kappa -1}\left(x,y\right)= & \frac{1}{16}[9\psi_{\kappa -1}\left(x-\frac{\eta_{\kappa }}{2},y-\frac{\eta_{\kappa }}{2}\right)\\ +3\;\psi_{\kappa -1}\left(x-\frac{\eta_{\kappa }}{2},y+\frac{3\eta_{\kappa }}{2}\right)\\ +\;3\psi_{\kappa -1}\left(x+\frac{3\eta_{\kappa }}{2},y-\frac{\eta_{\kappa }}{2}\right)\\ +\psi_{\kappa -1}\left(x+\frac{3\eta_{\kappa }}{2},y+\frac{3\eta_{\kappa }}{2}\right)] \end{array}$$

(3.1.11) $$\begin{array}{ll} P_{\kappa -1}^{\kappa }\psi_{\kappa -1}\left(x,y\right)= & \frac{1}{16}[9\psi_{\kappa -1}\left(x+\frac{\eta_{\kappa }}{2},y-\frac{\eta_{\kappa }}{2}\right)\\ +3\;\psi_{\kappa -1}\left(x+\frac{\eta_{\kappa }}{2},y+\frac{3\eta_{\kappa }}{2}\right)\\ +\;3\psi_{\kappa -1}\left(x-\frac{3\eta_{\kappa }}{2},y-\frac{\eta_{\kappa }}{2}\right)\\ +\psi_{\kappa -1}\left(x-\frac{3\eta_{\kappa }}{2},y+\frac{3\eta_{\kappa }}{2}\right)] \end{array}$$

for points marked *a*, *b*, *c*, and *d*, respectively. For 3D cases as shown in Figure 5, trilinear interpolation produces the following

(3.1.12) $$\begin{array}{ll} P_{\kappa -1}^{\kappa }\psi_{\kappa -1}\left(x,y,z\right)= & \frac{1}{64}[27\psi_{\kappa -1}\left(x+\frac{\eta_{\kappa }}{2},y-\frac{\eta_{\kappa }}{2},z\pm \frac{\eta_{\kappa }}{2}\right)\\ +9\;\psi_{\kappa -1}\left(x+\frac{\eta_{\kappa }}{2},y+\frac{3\eta_{\kappa }}{2},z\pm \frac{\eta_{\kappa }}{2}\right)\\ +\;9\;\psi_{\kappa -1}\left(x+\frac{\eta_{\kappa }}{2},y-\frac{\eta_{\kappa }}{2},z\mp \frac{3\eta_{\kappa }}{2}\right)\\ +9\;\psi_{\kappa -1}\left(x-\frac{3\eta_{\kappa }}{2},y-\frac{\eta_{\kappa }}{2},z\pm \frac{\eta_{\kappa }}{2}\right)\\ +\;3\;\psi_{\kappa -1}\left(x+\frac{\eta_{\kappa }}{2},y+\frac{3\eta_{\kappa }}{2},z\mp \frac{3\eta_{\kappa }}{2}\right)\\ +3\;\psi_{\kappa -1}\left(x-\frac{3\eta_{\kappa }}{2},y+\frac{3\eta_{\kappa }}{2},z\pm \frac{\eta_{\kappa }}{2}\right)\\ +\;3\;\psi_{\kappa -1}\left(x-\frac{3\eta_{\kappa }}{2},y-\frac{\eta_{\kappa }}{2},z\mp \frac{3\eta_{\kappa }}{2}\right)\\ +\psi_{\kappa -1}\left(x-\frac{3\eta_{\kappa }}{2},y+\frac{3\eta_{\kappa }}{2},z\mp \frac{3\eta_{\kappa }}{2}\right)] \end{array}$$

(3.1.13) $$\begin{array}{ll} P_{\kappa -1}^{\kappa }\psi_{\kappa -1}(x,y,z)= & \frac{1}{64}[27\psi_{\kappa -1}(x+\frac{\eta_{\kappa }}{2},y+\frac{\eta_{\kappa }}{2},z\pm \frac{\eta_{\kappa }}{2})\\ \; & +9\;\psi_{\kappa -1}(x+\frac{\eta_{\kappa }}{2},y-\frac{3\eta_{\kappa }}{2},z\pm \frac{\eta_{\kappa }}{2})\\ \; & +\;9\;\psi_{\kappa -1}(x+\frac{\eta_{\kappa }}{2},y+\frac{\eta_{\kappa }}{2},z\mp \frac{3\eta_{\kappa }}{2})\\ \; & +9\;\psi_{\kappa -1}(x-\frac{3\eta_{\kappa }}{2},y+\frac{\eta_{\kappa }}{2},z\pm \frac{\eta_{\kappa }}{2})\\ \; & +\;3\;\psi_{\kappa -1}(x+\frac{\eta_{\kappa }}{2},y-\frac{3\eta_{\kappa }}{2},z\mp \frac{3\eta_{\kappa }}{2})\\ \; & +3\;\psi_{\kappa -1}(x-\frac{3\eta_{\kappa }}{2},y-\frac{3\eta_{\kappa }}{2},z\pm \frac{\eta_{\kappa }}{2})\\ \; & +\;3\;\psi_{\kappa -1}(x-\frac{3\eta_{\kappa }}{2},y+\frac{\eta_{\kappa }}{2},z\mp \frac{3\eta_{\kappa }}{2})\\ \; & +\psi_{\kappa -1}(x-\frac{3\eta_{\kappa }}{2},y-\frac{3\eta_{\kappa }}{2},z\mp \frac{3\eta_{\kappa }}{2})] \end{array}$$

(3.1.14) $$\begin{array}{ll} P_{\kappa -1}^{\kappa }\psi_{\kappa -1}(x,y,z)= & \frac{1}{64}[27\psi_{\kappa -1}(x-\frac{\eta_{\kappa }}{2},y-\frac{\eta_{\kappa }}{2},z\pm \frac{\eta_{\kappa }}{2})\\ \; & +9\;\psi_{\kappa -1}(x-\frac{\eta_{\kappa }}{2},y+\frac{3\eta_{\kappa }}{2},z\pm \frac{\eta_{\kappa }}{2})\\ \; & +\;9\;\psi_{\kappa -1}(x-\frac{\eta_{\kappa }}{2},y-\frac{\eta_{\kappa }}{2},z\mp \frac{3\eta_{\kappa }}{2})\\ \; & +9\;\psi_{\kappa -1}(x+\frac{3\eta_{\kappa }}{2},y-\frac{\eta_{\kappa }}{2},z\pm \frac{\eta_{\kappa }}{2})\\ \; & +\;3\;\psi_{\kappa -1}(x-\frac{\eta_{\kappa }}{2},y+\frac{3\eta_{\kappa }}{2},z\mp \frac{3\eta_{\kappa }}{2})\\ \; & +3\;\psi_{\kappa -1}(x+\frac{3\eta_{\kappa }}{2},y+\frac{3\eta_{\kappa }}{2},z\pm \frac{\eta_{\kappa }}{2})\\ \; & +\;3\;\psi_{\kappa -1}(x+\frac{3\eta_{\kappa }}{2},y-\frac{\eta_{\kappa }}{2},z\mp \frac{3\eta_{\kappa }}{2})\\ \; & +\psi_{\kappa -1}(x+\frac{3\eta_{\kappa }}{2},y+\frac{3\eta_{\kappa }}{2},z\mp \frac{3\eta_{\kappa }}{2})] \end{array}$$

(3.1.15) $$\begin{array}{ll} P_{\kappa -1}^{\kappa }\psi_{\kappa -1}(x,y,z)= & \frac{1}{64}[27\psi_{\kappa -1}(x-\frac{\eta_{\kappa }}{2},y+\frac{\eta_{\kappa }}{2},z\pm \frac{\eta_{\kappa }}{2})\\ \; & +9\;\psi_{\kappa -1}(x-\frac{\eta_{\kappa }}{2},y-\frac{3\eta_{\kappa }}{2},z\pm \frac{\eta_{\kappa }}{2})\\ \; & +\;9\;\psi_{\kappa -1}(x-\frac{\eta_{\kappa }}{2},y+\frac{\eta_{\kappa }}{2},z\mp \frac{3\eta_{\kappa }}{2})\\ \; & +9\;\psi_{\kappa -1}(x+\frac{3\eta_{\kappa }}{2},y+\frac{\eta_{\kappa }}{2},z\pm \frac{\eta_{\kappa }}{2})\\ \; & +\;3\;\psi_{\kappa -1}(x-\frac{\eta_{\kappa }}{2},y-\frac{3\eta_{\kappa }}{2},z\mp \frac{3\eta_{\kappa }}{2})\\ \; & +3\;\psi_{\kappa -1}(x+\frac{3\eta_{\kappa }}{2},y-\frac{3\eta_{\kappa }}{2},z\pm \frac{\eta_{\kappa }}{2})\\ \; & +\;3\;\psi_{\kappa -1}(x+\frac{3\eta_{\kappa }}{2},y+\frac{\eta_{\kappa }}{2},z\mp \frac{3\eta_{\kappa }}{2})\\ \; & +\;\psi_{\kappa -1}(x+\frac{3\eta_{\kappa }}{2},y-\frac{3\eta_{\kappa }}{2},z\mp \frac{3\eta_{\kappa }}{2})] \end{array}$$

for points *a* and *c*, *b* and *d*, *e* and *g*, *f* and *h*, respectively. The coarse grid operator ***L***_κ−1_ is generated in a way that is analogous to the computation of ***L***_κ_ on the fine grid. Alternatively, the Galerkin coarse grid operator can be used:

(3.1.16) $$\begin{array}{l} L_{\kappa -1}=R_{\kappa }^{\kappa -1}L_{\kappa }P_{\kappa -1}^{\kappa } \end{array}$$

where $R_{\kappa }^{\kappa -1}$ and $P_{\kappa -1}^{\kappa }$ are appropriately chosen transfer operators (Trottenberg et al., 2001).

A FAS V-cycle consists of two iterating components. The outer time iteration travels through one V–loop, starting from the finest mesh level, looking for the fixed point solution of $\text{L}\left(\psi_{i,j,k}^{a,r},\psi_{i,j,k}^{a,r-1}\;\right)=\text{R}\left(\psi_{i,j,k}^{a-1},\psi_{i,j,k}^{a,r-1}\;\right)$ as derived in Supplement (Multigrid V–Cycle Iterations). Starting from **ψ**^*a*, 0^, each successive V–cycle produces an approximation that is converging toward the fixed point solution, **ψ**^*a*^, at the current time step *a*. After r^*^ outer time iterations (after r^*^ V–loops), if the approximation **ψ**^a, r*^ results in an error within a tolerable bound, an approximated solution for the current time step is established by **ψ**^*a*^ = **ψ**^a, r*^. The inner iteration is the non-linear Gauss-Seidel relaxation as described in Supplement (Non-linear Gauss–Seidel Relaxations) and given in Supplementary Equation (1.4.39). This procedure occurs twice on each level. Relaxation first takes place during the error smoothing step while traveling down from the finest to the coarsest grid level, and again after coarse grid correction while going up from the coarsest to the finest grid level. Hence, the starting approximation for the error smoothing step is obtained from the previous iteration $\psi_{\kappa }^{a,r,0}=\psi_{\kappa }^{a,r-1}$ or restricted from the relaxed fine-grid approximation $\psi_{\kappa }^{a,r,0}=R_{\kappa }^{\kappa -1}\psi_{\kappa +1}^{a,r,\nu_{1}}$, whereas the coarse-grid-corrected approximation $\psi_{\kappa }^{a,r,0}=\psi_{\kappa }^{a,r,CGC}$ is used during the correction step.

The computation of a new time iterate $\psi_{\kappa }^{a,r}$ using an adaptive FAS multigrid-cycle, starting with any of the refined or global grid levels described in the Multilevel Mesh Refinement subsection, can be done recursively using a two–level model as summarized in the operator below (Brandt, 1977; Trottenberg et al., 2001; Wise et al., 2007).

Recursive adaptive cycle operator:

(3.1.17) $$\begin{array}{l} \psi_{\kappa }^{a,r}=\text{ADAPFAS}\left(\kappa ,\gamma ,\nu_{0},\nu_{1},\nu_{2},\;\psi_{\kappa }^{a,r-1},\psi_{\kappa -1}^{a,r-1},L_{\kappa },\;R_{\kappa }\right) \end{array}$$

- Pre-smoothing – Compute a smoothed approximation $\psi_{\kappa }^{a,r,\nu_{1}}$ by applying ν_1_ smoothing steps to $\psi_{\kappa }^{a,r-1}$ on Ω_κ_:
  (3.1.18) $$\begin{array}{l} \psi_{\kappa }^{a,r,\nu_{1}}=\text{SMOOTH}\left(\nu_{1},\;\psi_{\kappa }^{a,r-1},\;L_{\kappa },\;R_{\kappa }\right) \end{array}$$
- Coarse-grid correction: – Initialize the coarse-grid iterate:
  (3.1.19) $$\psi_{\kappa -1}^{a,r,0}=\{\begin{array}{cc} R_{\kappa }^{\kappa -1}\psi_{\kappa }^{a,r,\nu_{1}} & on\;\Omega_{\kappa -1}\cap \Omega_{\kappa }\\ \psi_{\kappa -1}^{a,r-1} & on\;\Omega_{\kappa -1}-\Omega_{\kappa } \end{array}$$
  – Update the ghost cells on κ − 1 level using interpolation and exchange for $\psi_{\kappa -1}^{a,r,0}$ on neighboring patches.
  – Compute the coarse-grid RHS:
  (3.1.20) $$R_{\kappa -1}=\{\begin{array}{ll} R_{\kappa }^{\kappa -1}\left[R_{\kappa }-L_{\kappa }\left(\psi_{\kappa }^{a,r,\nu_{1}}\right)\right] & \;\\ \;+L_{\kappa -1}\left(R_{\kappa }^{\kappa -1}\psi_{\kappa }^{a,r,\nu_{1}}\right) & on\;\Omega_{\kappa -1}\cap \Omega_{\kappa }\\ R_{\kappa -1} & on\;\Omega_{\kappa -1}-\Omega_{\kappa } \end{array}$$
  – Compute an approximate solution ${\bar{\psi }}_{\kappa -1}^{a,r}$ of the following coarse-grid equation on Ω_κ−1_:
  (3.1.21) $$\begin{array}{l} L_{\kappa -1}\left({\bar{\psi }}_{\kappa -1}^{a,r}\right)=R_{\kappa -1} \end{array}$$
  If κ = κ_min_+1, employ a direct solver or perform ν_0_ smoothing steps:
  (3.1.22) $$\begin{array}{l} {\bar{\psi }}_{\kappa_{\min}}^{a,r}=\text{SMOOTH}\left(\nu_{0},\;\psi_{\kappa_{\min}}^{a,r,0},\;L_{\kappa_{\min}},\;R_{\kappa_{\min}}\right) \end{array}$$
  If κ > κ_min_ + 1, solve Equation (3.1.21) by employing γ adaptive FAS cycle using $\psi_{\kappa -1}^{a,r,0}$ as initial approximation:
  (3.1.23) $$\begin{array}{l} {\bar{\psi }}_{\kappa -1}^{a,r}=\text{ADAPFAS}(\kappa -1,\gamma ,\nu_{0},\nu_{1},\nu_{2},\psi_{\kappa -1}^{a,r,0}\\ \;\psi_{\kappa -2}^{a,r-1}L_{\kappa -1},R_{\kappa -1}) \end{array}$$
  – Arrive at approximation for time iteration *r* on Ω_κ−1_−Ω_κ_:
  (3.1.24) $$\begin{array}{l} \psi_{\kappa -1}^{a,r}={\bar{\psi }}_{\kappa -1}^{a,r} \end{array}$$
  – Compute coarse-grid correction on Ω_κ−1_∩Ω_κ_:
  (3.1.25) $$\begin{array}{l} {\text{e}}_{\kappa -1}^{a,r}={\bar{\psi }}_{\kappa -1}^{a,r}-\psi_{\kappa -1}^{a,r,0} \end{array}$$
  – Interpolate the correction and compute the coarse-grid corrected approximation on Ω_κ_:
  (3.1.26) $$\begin{array}{l} \psi_{\kappa }^{a,r,CGC}=\psi_{\kappa }^{a,r,\nu_{1}}+P_{\kappa -1}^{\kappa }{\text{e}}_{\kappa -1}^{a,r} \end{array}$$
  – Update the ghost cells on κ level using interpolation and exchange for $\psi_{\kappa }^{a,r,CGC}$ on neighboring patches.
- Post-smoothing:
  Compute $\psi_{\kappa }^{a,r}$ by applying ν_2_ smoothing steps to $\psi_{\kappa }^{a,r,CGC}$ on Ω_κ_:
  (3.1.27) $$\begin{array}{l} \psi_{\kappa }^{a,r}=\text{SMOOTH}\left(\nu_{2}\;,\;\psi_{\kappa }^{a,r,CGC},\;L_{\kappa },\;R_{\kappa }\right) \end{array}$$
  – Update the ghost cells on κ level using interpolation and exchange for $\psi_{\kappa }^{a,r}$ on neighboring patches.

V– or W–cycle is defined via the cycle index γ = 1 and γ = 2, respectively. The initial value used in V-cycles may come from the converged solution at previous time **ψ**^*a*, 0^ = **ψ**^*a*−1^ or from coarse-grid approximations as in FMG method described in the next section. The SMOOTH routine in Equations (3.1.18), (3.1.22), and (3.1.27) uses Red-Black (or odd-even) ordering for the relaxation process, with ν_*b*_ extra smoothing steps for *d*_κ_ cells within the boundaries.

#### Adaptive FMG structure

**Figure 6:**
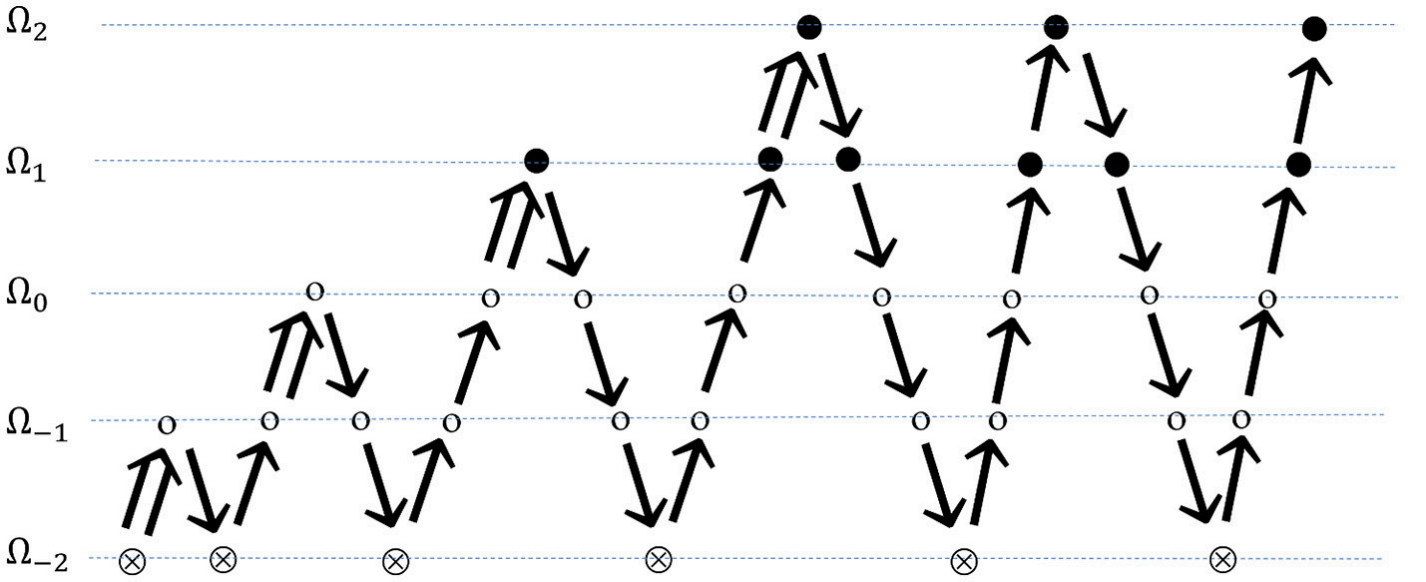
FMG on self-adaptive grids. Beginning on a series of global coarse grids, finer grid is introduced a layer per multigrid cycle. ⊗ represents solution on the coarsest grid; ⊗ represents smoothing on global grid levels; ∙ represents smoothing on refined grid levels; 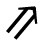
indicates FMG interpolation; ↘ indicates restriction; and ↗ indicates prolongation.

In FMG, a better initial approximation for finer-grid iteration is obtained from a coarser-grid level (Kronsjö and Dahlquist, 1972; Brandt, 1977). The procedure begins with a set of solutions on the coarsest grid, followed by interpolation of the solution set to a fine-grid level, providing an initial guess for the fine-grid multigrid approximation. After a few multigrid cycles on the fine-grid level, the solution is again interpolated to the next fine-grid level. This proceeds until the finest grid level has been reached, if refinement is needed for each level κ > 0. Discretization accuracy can be reached on each grid level within a few multigrid cycles in this manner. The structure of full multigrid is illustrated in Figure 6.

**Figure 7:**
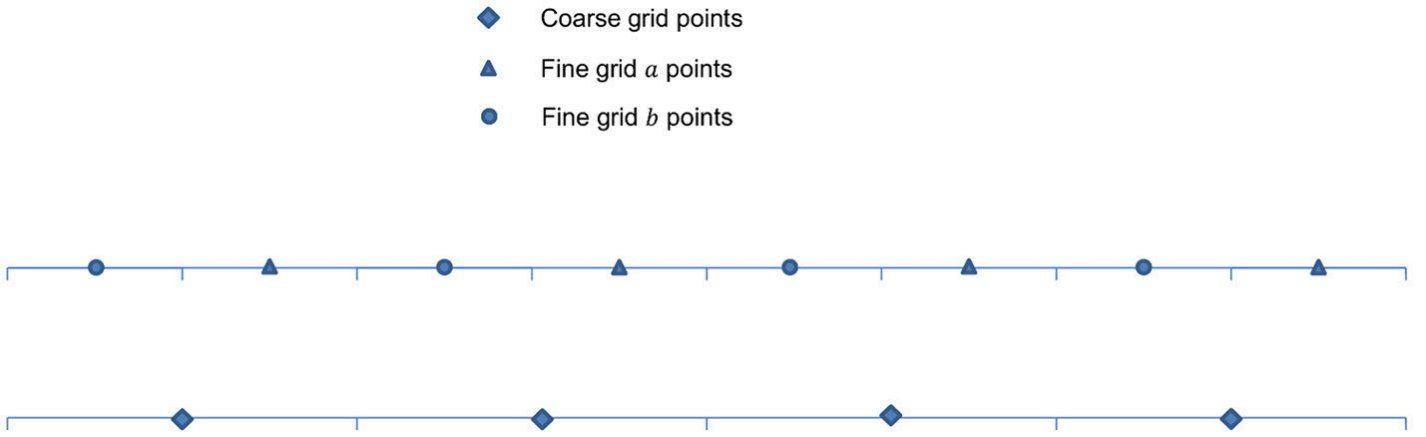
Arrangement of unknowns in 1D cell-centered discretization.

A higher order scheme is normally employed for an FMG interpolation. Cubic Lagrangian interpolation is one such method used in the FMG interpolation steps to compute an approximation at the fine grid. In 1D as depicted in Figure 7, approximation **ψ**_κ_(*x*) at the fine grid point can be computed as

(3.1.28) $$\begin{array}{l} \prod_{\kappa -1}^{\kappa }\psi_{\kappa -1}(x)=\frac{1}{128}[-7\psi_{\kappa -1}(x-\frac{5\eta_{\kappa }}{2})\\ \;+105\psi_{\kappa -1}(x-\frac{\eta_{\kappa }}{2})\\ \;+35\psi_{\kappa -1}(x+\frac{3\eta_{\kappa }}{2})\\ \;-5\psi_{\kappa -1}(x+\frac{7\eta_{\kappa }}{2})] \end{array}$$

(3.1.29) $$\begin{array}{l} \prod_{\kappa -1}^{\kappa }\psi_{\kappa -1}(x)=\frac{1}{128}[-7\psi_{\kappa -1}(x-\frac{5\eta_{\kappa }}{2})\\ \;+105\psi_{\kappa -1}(x-\frac{\eta_{\kappa }}{2})\\ \;+35\psi_{\kappa -1}(x-\frac{3\eta_{\kappa }}{2})\\ \;-5\psi_{\kappa -1}(x+\frac{7\eta_{\kappa }}{2})] \end{array}$$

for point *a* and *b* respectively. Cubic interpolation in the *y*- and *z*-directions for bicubic and tricubic interpolation, in 2D and 3D cases, respectively, are analogous to the 1D calculation shown.

FMG can be performed on locally refined grids which are defined *a priori*. Here, we use self-adaptive FMG, where refined grids may or may not be generated, one layer at a time, based on some predefined criteria. Self-adaptive FMG advances as follows:

1. Perform FMG on a series of global grids until a satisfactory accuracy has been obtained on grid level κ = 0.
2. Determine if refining the finest grid would be beneficial, based on the criteria outlined in the Multilevel Mesh Refinement subsection. If no, iteration stops. If yes, refine grid in local regions.
3. Use cubic interpolation on the current fine-grid (κ) approximation to compute an initial guess for the refined grid level (κ + 1), proceed by performing a number of multigrid cycles on the refined level until the required accuracy has been satisfied.
4. If the finest level κ_max_ has been reached, the time iteration stops. Otherwise, return to Step (2).

Let *tol*_κ_, γ_κ_, and *r*_κ_ be the residual tolerance, cycle index, and number of multigrid cycles performed on level κ. The self-adaptive FMG-FAS iteration is given by the following:

**Table:** 

Initialize$\psi_{\kappa }^{a=0}$, κ = κ_min_, …, κ_max_
Setγ_κ_, *r*_κ_, and *tol*_κ_, κ = κ_min_, …, κ_max_
For *a* = 1, *a*_max_
Set $\psi_{\kappa }^{a,r=0}=\psi_{\kappa }^{a-1},\kappa =\kappa_{\min},\ldots ,\kappa_{\max}$
(3.1.30) $$\psi_{\kappa }^{a}=\text{FMG}\left(\kappa ,\gamma ,\nu_{0},\nu_{1},\nu_{2},{\text{tol}}_{\text{k}}\right),\kappa =\kappa_{\min},\cdots ,\kappa_{\max}$$
End For

The recursive FMG routine from Rude (1993) is modified for Equation (3.1.30) above and shown as the following:

**Table:** 

For κ = κ_min_, κ_max_
If κ = κ_min_, employ a direct solver or perform ν_0_ smoothing steps at the coarsest level and return the solution:
${\bar{\psi }}_{\kappa_{\min}}^{a,0,\nu_{0}}=\text{SMOOTH}\left(\nu_{0},\;\psi_{\kappa_{\min}}^{a,r=0},\;L_{\kappa_{\min}},\;R_{\kappa_{\min}}\right)$
Else
If κ < 0, perform *r*_κ_ multigrid V-cycles at level κ:
*r* = *r* + 1
$\psi_{\kappa }^{a,r}=\text{ADAPFAS}(\kappa ,\gamma_{\kappa },tol_{\kappa },\nu_{0},\nu_{1},\nu_{2},\;\psi_{\kappa }^{a,r-1},\psi_{\kappa -1}^{a,r-1},$ ***L***_κ_**, *R***_κ_)
Else perform multigrid V-cycles at level κ until the approximation yields an error within bound:
Do
*r* = *r* + 1
$\psi_{\kappa }^{a,r}=\text{ADAPFAS}(\kappa ,\gamma_{\kappa },tol_{\kappa },\nu_{0},\nu_{1},\nu_{2},\;\psi_{\kappa }^{a,r-1},\psi_{\kappa -1}^{a,r-1},$ ***L***_κ_**,** ***R***_κ_)
While $||R_{\kappa }-L_{\kappa }\left(\psi_{\kappa }^{a,r}\right)||>tol_{\kappa }$
End If
If κ < 0, interpolate the coarse-grid solution to obtain fine-grid initial guess: $\psi_{\kappa +1}^{a,r-1}=\Pi_{\kappa }^{\kappa +1}\;\psi_{\kappa }^{a,r-1}$
Else
If 0 ≤ κ < κ_max_, flag cells on level κ to determine if refinement is needed: $F_{\kappa }^{a,r-1}=\text{FLAG}\left(\;\psi_{\kappa }^{a,r-1}\right)$
If any cells are flagged to be refined, generate blocks on level κ + 1 accordingly: $\begin{array}{l} B_{\kappa +1}=\text{BLOCKGEN }(threshold\text{\_}eff\text{ , }threshold\text{\_}size\text{, }min\text{\_}eff\text{ ,}\\ \;F_{\kappa }^{a,r-1}) \end{array}$
Then interpolate the coarse-grid solution to obtain fine-grid initial guess:
$\psi_{\kappa +1}^{a,r-1}=\Pi_{\kappa }^{\kappa +1}\;\psi_{\kappa }^{a,r-1}$
End If
End For

A parameter σ is used to adapt the accuracy to the different level of meshes. Tolerance is reduced by σ for each finer mesh level, $tol_{\kappa }=\frac{tol_{\kappa -1}}{\sigma }$. The parameter σ and the initial value of tolerance have to be selected with care in order to ensure the overall accuracy of the final grid structure.

### System solution

Next, we applied the fully adaptive non-linear FMG algorithm to solve the desmoplastic tumor model. The set of tumor model related parameters, constants, source terms, rate expressions, and adjustment factors are listed in Supplementary Tables 1–6. We assume that the tissue consists of a fixed fraction of liquid interstitial-fluid phase (ϕ_β_ = 0.2) and solid cell-ECM phase (ϕ_α_ = 0.8). We also assume that the solid cell-ECM phase is only composed of viable tumor cells (${\tilde{\phi }}_{V}$), dead tumor cells (${\tilde{\phi }}_{D}$), extracellular matrix ECM (${\tilde{\phi }}_{E}$), and healthy host cells (${\tilde{\phi }}_{H}$). Nutrients, waste products, and tumorigenic species are assumed to be carried in the liquid phase, while the myofibroblastic cell species (${\tilde{F}}_{E}$), blood (${\tilde{B}}_{n}^{E}$) and lymphatic (${\tilde{L}}_{n}^{E}$) microvessels are assumed to take up negligible volume and exist within the continuous ECM component.

We assume that the source and sink of viable tumor cells comes from cell proliferation and losses to necrosis respectively. The dead tumor cell species hence comes from the necrosed viable tumor cells and undergoes lysis. The ECM is assumed to be secreted by the myofibroblastic cell species and degraded by the matrix degrading enzyme species ($\tilde{m}$). The healthy host cells species is assumed to be homeostatic. Nutrients such as oxygen (ñ) and glucose ($\tilde{g}$) enter the tissue via blood vessels and are consumed mainly by the viable tumor species. Tumor growth factors ($t\tilde{g}f$) and tumor angiogenic factors ($t\tilde{a}f$) are assumed to be secreted by viable tumor cells and can undergo degradation. Tumor angiogenic factors also have an uptake term by proliferating vessels. Myofibroblastic cell species is assumed to go through proliferation, apoptosis, and necrosis, while the blood and lymphatic vessels undergo restructuring and potential loss due to crushing by the surrounding tissue pressure. To slightly perturb the symmetry of tumor progression, we let the fraction of blood vessels that are sprouting be different in different regions of the domain, ranging from 0.1 to 0.8.

As described in **Methods**, all PDEs were discretized using the Crank-Nicolson method. To solve the set of cell-centered discretized equations, we applied full multigrid algorithm and adaptive full approximation scheme V-cycle with Gauss-Seidel Red-Black smoothing to the multiple grid level system. Here, we use a total of five grid levels as depicted in Figure 1, with three increasingly refined global levels (κ = −2, −1, 0) covering the entire domain Ω and two adaptively refined levels (κ = 1, 2). The domain is Ω = (0, 40)^3^ and the mesh sizes for the finest global level to the finest adaptively refined level are η_0_ = 40/32, η_1_ = 40/64, and η_2_ = 40/128. The time step-size used is θ = 1 × 10^−2^ and minimum tolerance is set at 5 × 10^−4^. Key solver related parameters are listed in Table 1.

**Table 1:** Solver related parameters.

γ = 1	Cycle index (1 for V-cycle, 2 for W-cycle)
ν_0_ = 4	Number of smoothing steps on the coarsest grid level κ_−2_ (set to ν_1_+ν_2_)
ν_1_ = 2	Number of pre-correction smoothing steps during a V-cycle
ν_2_ = 2	Number of post-correction smoothing steps during a V-cycle
ν_*b*_ = 2	Number of extra smoothing steps near boundaries
$d_{\kappa }=\{\begin{array}{cc} 2^{\kappa +2} & ,for\kappa \leq 0\\ \frac{min\left(d_{x},d_{y},d_{z}\right)}{8} & ,for\kappa >0 \end{array}$	Number of cells inward from a boundary to be included in the near-boundary extra smoothing steps
	Set to $2^{\kappa -\kappa_{\min}}$ for κ ≤ 0
	Set to $\frac{1}{8}$ of the minimum block dimension (in x-, y-, and z-direction) for κ > 0
*r*_κ_ = 1	Number of V-cycles (iterations) performed before the finest global level is reached (for levels κ_min_ < κ < 0, which is *r*_−1_ here)
σ = 4	Factor by which the tolerance is reduced from level κ to κ + 1
*C*_κ_ = 0.05	Critical value used in the undivided gradient test in the FLAG routine
*threshold*_*eff* = 0.9	Upper limit of efficiency, any blocks below this limit will go through the splitting algorithm. (Used in the BLOCKGEN routine)
*threshold*_*size* = 10^3^	Minimum size of a block allowed. (Used in the BLOCKGEN routine)
min_*eff* = 0.5	Lowest limit of efficiency allowed, any blocks below this limit will be bisected. (Used in the BLOCKGEN routine)

The center of the computational domain is simply seeded with viable tumor cells (${\tilde{\phi }}_{V}=0.65$) at the beginning of the simulation. We assume that there are no dead tumor cells (${\tilde{\phi }}_{D}=0$) initially and the ECM is distributed evenly (${\tilde{\phi }}_{E}=0.35$) across the domain. The healthy host cells thus take up the remaining volume in the solid phase of the tissue. The initial tumor shape is therefore rectangular with a sharp interface. The simplified source term for the viable tumor cell species used in Equation (2.1), where only mitosis and necrosis are considered, is given by:

(5.1) $$\begin{array}{l} {\tilde{S}}_{v}=\{{\tilde{\lambda }}_{M,V}\tilde{n}\left(1+t\tilde{g}f\right)Η\left(\tilde{n}-{\tilde{n}}_{h}\right)\\ \;-{\tilde{\lambda }}_{N,V}\left[1-Η\left(\tilde{n}-{\tilde{n}}_{v,V}\right)Η\left(\tilde{g}-{\tilde{g}}_{v,V}\right)\right]\}{\tilde{\phi }}_{V}, \end{array}$$

where H is the Heaviside function, ñ_*h*_ = 0.3 is the hypoxic level of oxygen, ${\tilde{\lambda }}_{M,V}$ = 1 and ${\tilde{\lambda }}_{N,V}=3$ are the rate constants of mitosis and necrosis, respectively, for viable tumor cells. ñ_*v, V*_ = 0.21 and ${\tilde{g}}_{v,V}=0.1$ are the oxygen and glucose viability limits, respectively, for the viable tumor cell species. The mitosis rate is assumed to be upregulated by the level of tumor growth factors. The center of the tumor mass experiences a significant drop in nutrient levels. When the oxygen level drops below the hypoxic level, viable tumor cells cease to reproduce. Necrosis takes place if the oxygen level falls below the viability limit, where viable tumor cells necrosed to dead tumor cells. The source term for the dead tumor cell species in Equation (2.2) is hence given by

(5.2) $$\begin{array}{l} {\tilde{S}}_{D}={\tilde{\lambda }}_{N,V}\left[1-H\left(ñ-{ñ}_{v,V}\right)H\left(\tilde{g}-{\tilde{g}}_{v,V}\right)\right]{\tilde{\phi }}_{V}-{\tilde{\lambda }}_{L,D}{\tilde{\phi }}_{D}\;, \end{array}$$

where ${\tilde{\lambda }}_{L,D}=1$ is the lysis rate constant for the dead tumor cell species. The lysed dead tumor cells are assumed loss to the interstitial fluid. The other major solid component of the tissue is the ECM, and its source term used in Equation (2.3) is

(5.3) $$\begin{array}{l} {\tilde{S}}_{E}={\tilde{\lambda }}_{F,E}A_{F,E}\tilde{F}-{\tilde{\lambda }}_{de,E}\tilde{m}{\tilde{\phi }}_{E}\;, \end{array}$$

where ${\tilde{\lambda }}_{F,E}=5$ is the secretion rate constant of ECM by the myofibroblastic cell species $\tilde{F}$ and ${\tilde{\lambda }}_{de,E}=5$ is the degradation rate constant of ECM by the matrix degrading enzyme species $\tilde{m}$. Factors affecting the rate constant of ECM secretion by the myofibroblastic cell species are included in the adjustment factor $A_{F,E}$ given by

(5.4) $$\begin{array}{l} A_{F,E}=\begin{array}{c} \left(1-{\tilde{\phi }}_{T}-{\tilde{\phi }}_{E}\right)\left(1+t\tilde{g}f\right)\left[1+F_{n,E}^{F}\;\frac{{ñ}_{h}-ñ}{{ñ}_{h}-{ñ}_{v,F}}H\left({ñ}_{h}-ñ\right)\right]\\ H\left(ñ-{ñ}_{v,F}\right)H\left(t\tilde{g}f-{t\tilde{g}f}_{F,E}\right)H\left(1-{\tilde{\phi }}_{T}-{\tilde{\phi }}_{E}\right) \end{array} \end{array}$$

where $F_{n,E}^{F}=2$ is the effective factor of hypoxia on upregulating the production of ECM by myofibroblastic cells and ñ_*v, F*_ = 0.21 is the oxygen viability limit of the myofibroblastic cell species. The constant ${t\tilde{g}f}_{F,E}=0.2$ is a lower threshold of tumor growth factors, below which the production of ECM by the myofibroblastic cell species is assumed negligible. A complete list of source terms, rate expressions, and adjustment factors is shown in Supplementary Table 6.

**Figure 8:**
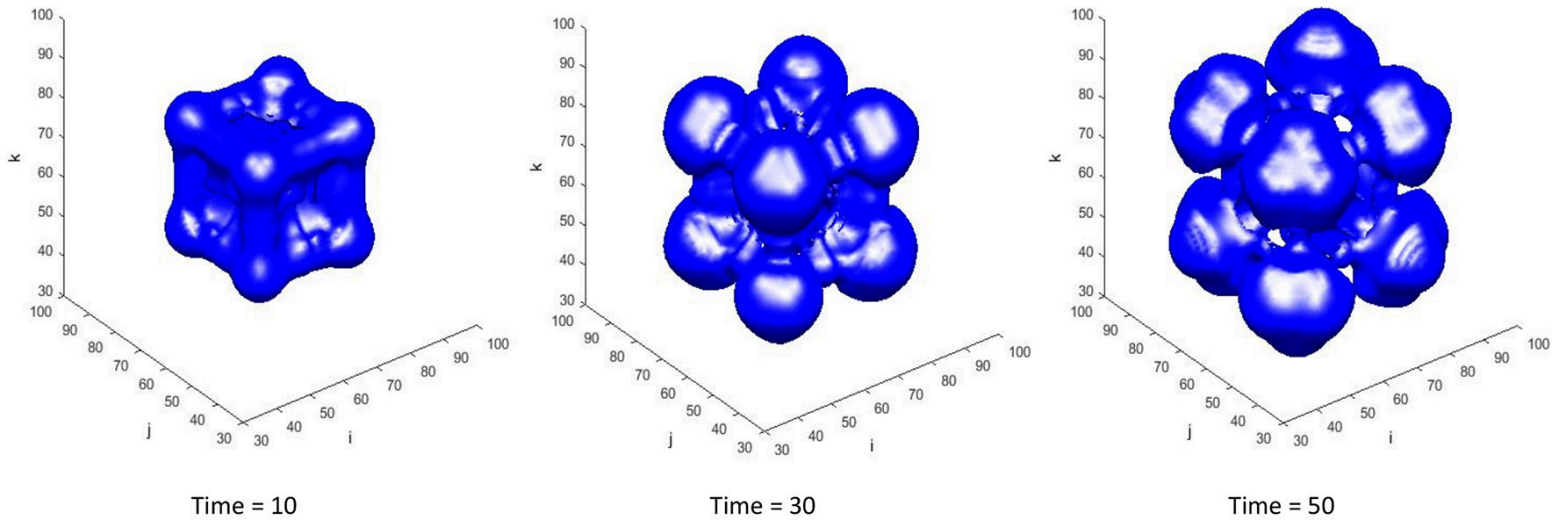
The transient ϕ_*T*_ = 0.15 isosurface at time = 10, 30, and 50. The total tumor volume fraction ϕ_*T*_ = ϕ_*V*_ + ϕ_*D*_. The center of the domain is initially seeded with viable tumor cells ϕ_*V*_ = 0.65. The starting extracellular matrix volume fraction is set to be homogenously distributed across the domain at ϕ_*E*_ = 0.35. The remaining volume fraction is thus made up by healthy host cells, denoted by ϕ_*H*_.

**Figure 9:**
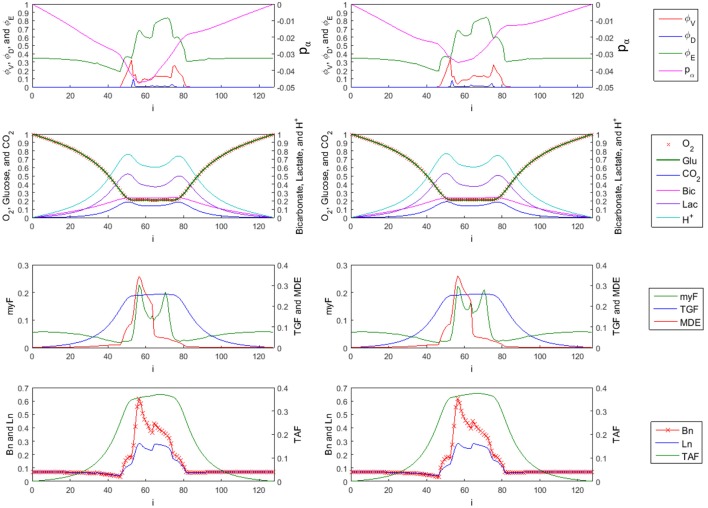
1-D Profiles of species with trilinear (left side) vs. cubic (right side) interpolation used in FMG-interpolation at time = 10 (sliced at j = k = 58). First row: tumor viable species ${\tilde{\phi }}_{V}$, dead species ${\tilde{\phi }}_{D}$, and ECM species ${\tilde{\phi }}_{E}$. The overall tumor pressure is labeled by *p*_α_. Second row: diffusible substances driving the tumor evolution, including oxygen (O_2_), glucose (Glu), carbon dioxide (CO_2_), bicarbonate (Bic), lactate (Lac), and hydrogen ions (H^+^). Third row: Concentration of myfibroblasts (myF), tumor growth factors (TGF), and matrix degrading enzymes (MDE). Fourth row: corresponding density of blood vasculature (Bn), lymphatic vasculature (Ln), and tumor angiogenic factors (TAF). The myofibrobrastic cell species density in tissue (shown in the third row of plots as myF) is computed by $\tilde{F}={\tilde{F}}_{E}{\tilde{\phi }}_{E}$. Similarly, the blood and lymphatic vessel densities in tissue (shown in the last row of plots as Bn and Ln) are given by ${\tilde{B}}_{n}={\tilde{B}}_{n}^{E}{\tilde{\phi }}_{E}$ and ${\tilde{L}}_{n}={\tilde{L}}_{n}^{E}{\tilde{\phi }}_{E}$ respectively.

The transient tumor progression is shown in Figure 8, via tracking the evolution of the tumor isosurface ${\tilde{\phi }}_{T}=0.15$. With the specific set of constants and parameters used in this case, the tumor undergoes regression in the time frame shown. Nutrients levels drop below the viability limit in the center of the tumor mass, causing dead tumor cells to accumulate from necrosed viable tumor cells, which are then lysed from the tumor mass. The increase in tumor growth factors in the tumor region upregulates the secretion of ECM by the myofibroblastic cell species, resulting in a high ECM environment within the tumor mass (as in Figure 9).

As mentioned in Results (Adaptive FMG Structure), a higher order scheme is normally used in the FMG interpolation step when interpolating the solution to a finer grid level. Trilinear interpolation as shown in Equations (3.1.12–3.1.15) and cubic interpolation given in Equations (3.1.28) and (3.1.29) are used in the FMG interpolation step and the simulation results for time = 10 are compared in Figure 9. The ECM and viable tumor levels are slightly higher in the case where cubic interpolation is used, resulting in higher solid cell–ECM phase pressure. Since the myofibroblastic cell species and vessels are assumed to reside within the ECM component, the higher ECM volume fraction in the high order scheme case also results in higher myofibroblastic cell species (presented in green as myF) and vessel densities (presented in red as Bn for blood and blue as Ln for lymphatic) in tissue. Concentration profiles of interstitial fluid based species show no visible difference in the two cases.

**Figure 10:**
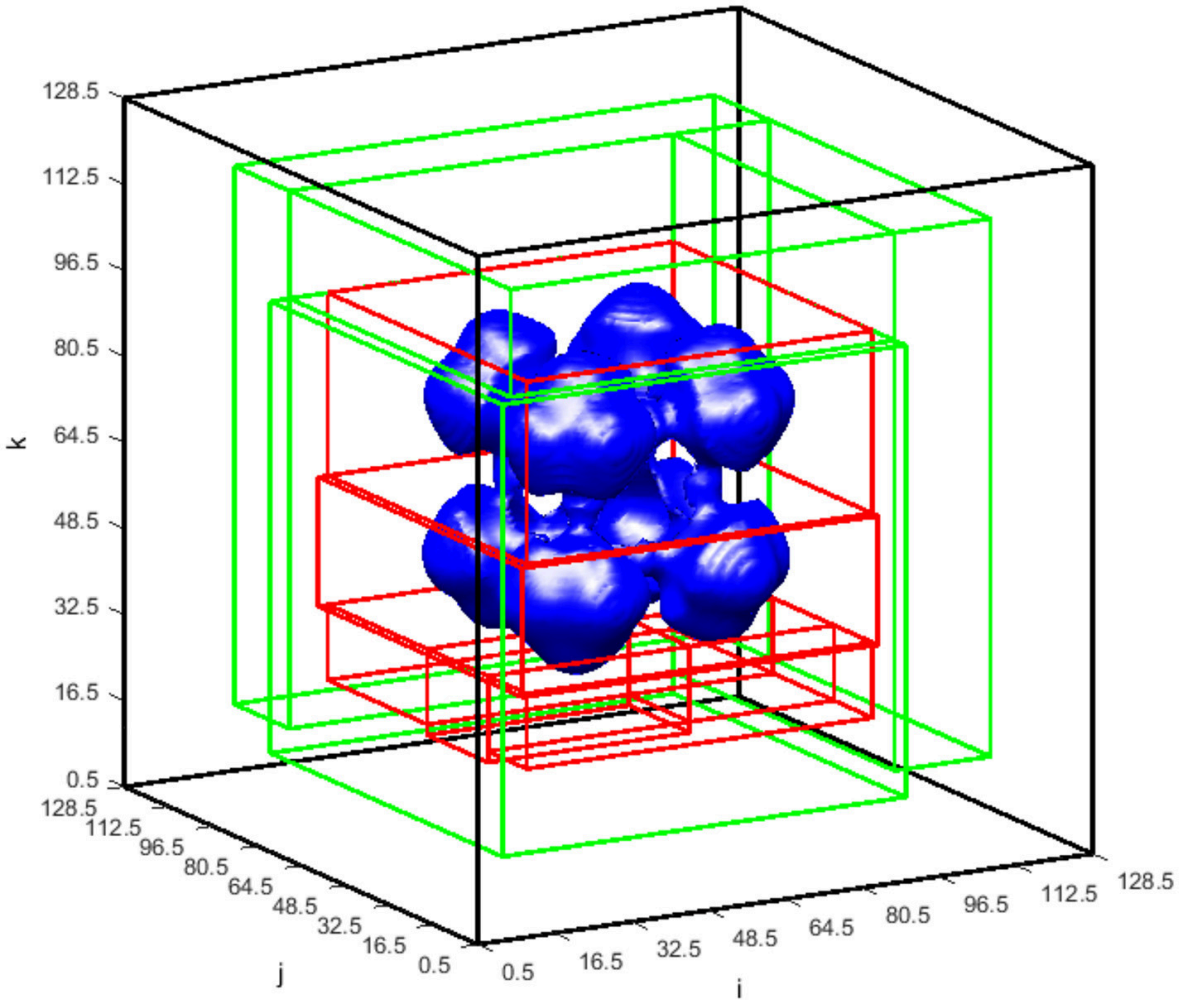
ϕ_*T*_ = 0.15 isosurface at time = 50 shown with the blocks of the block-structured mesh in the global and refined levels. There is one block (black) covering the entire global domain on the global level = 0 with the mesh spacing η_0_ = 40/32. There are two levels of refinement. Blocks covering the refined level = 1 (green) correspond to mesh spacing η_1_ = 40/64, whereas blocks on the refined level = 2 (red) correspond to mesh spacing η_2_ = 40/128.

The adaptive block-structured mesh system for the simulation in Figures 8, 9 at time = 50 is shown in Figure 10. For global level κ = 0, there is one block (shown in black) covering the entire domain with mesh size η_0_ = 40/32. In the first level of refinement at level κ = 1, there are four blocks (shown in green) with mesh size η_1_ = 40/64. In the next level of refinement at level κ = 2 and within the coarser domain Ω_1_, there are six blocks (shown in red) with mesh size η_2_ = 40/128. As shown in Table 1, the critical value of *C*_κ_ = 0.05 in Equation (3.1.1) is used in the undivided gradient test to flag cells for refinement. We also buffer each flag cell by flagging four cells surrounding it in each direction, creating a cube of 9 × 9 × 9 flagged cells, or less if it is near any external or ghost boundaries. The *thresholdsize* sets the minimum block size to 10^3^, resulting in overall bigger blocks generated by the BLOCKGEN routine in Equation (3.1.4).

**Figure 11:**
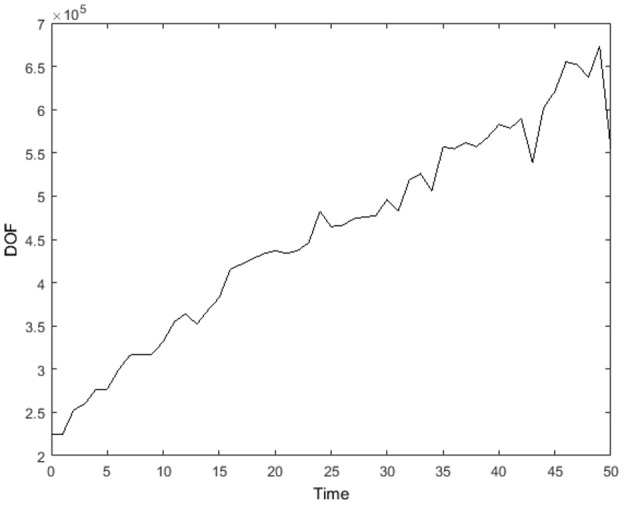
Degree of freedom, represented by total cell-centered grid points in the 3D domain, from time = 0 to 50. Here, external boundary and internal (ghost) boundary grid points are not accounted.

In Figure 11, the transient degree of freedom (DOF) for the simulation in Figures 8, 9 is plotted. The degree of freedom is represented by the total cell-centered grid points in the 3D domain. The mesh with just the three global levels (κ = −2, −1, 0) has only 37,376 DOF. In the first iteration, both refinement levels (κ = 1, 2) begin to contain flagged cells and the mesh now has 224,640 DOF. At time = 50, there are 554,568 DOF, increased by a factor of 2 from the first iteration.

A 3D convergence test as described in Wise et al. (2007) was done by varying grid and time-step sizes. Three simulations were performed where grid spacings used for the root level κ = 0 are 16^3^, 32^3^, and 64^3^, respectively. We focused on the convergence of the tumor volume fraction, ${\tilde{\phi }}_{T}$, and in all three cases, data were interpolated to the cell centers of their global uniform grids corresponding to their finest mesh η_κ_max__. With κ_max_ = 2, the grid sizes η_2_ and the corresponding time step sizes θ, which follow the linear refinement path θ = 0.032η, are outlined in Table 2. The errors were calculated by comparing cell center data to the averaged cell center data of the finer grid set, and the *l*_2_ norms of the errors were used to obtain the convergence rates as shown in Wise et al. (2007). The errors and the rate of convergence are also listed in Table 2. The results show that the algorithm presented herein is first-order accurate. Even though the discretization is second order in both time and space, the first-order rate of convergence attained, as also reported by Wise et al. (2011), is expected since the surface adhesion terms in Supplementary Equation (1.2.6) were treated explicitly. Non-smooth functions used in adjustment factors and first order interpolation function used in prolongation are other potential contributing factors to the first-order convergence.

**Table 2:** Errors and convergence rate.

Global level κ = 0 grids	16^3^		32^3^		64^3^
Grid sizes for η_κ_max__ = η_2_	$\frac{40}{64}$		$\frac{40}{128}$		$\frac{40}{256}$
Time step sizes θ	2 × 10^−2^		1 × 10^−2^		5 × 10^−3^
Error		3.41765 × 10^−3^		1.76463 × 10^−3^	
Rate			0.95364		

## Discussion

This paper illustrates the application of a fully adaptive, non-linear full multigrid, finite-difference algorithm to solve a diffuse interface desmoplastic tumor system (Ng and Frieboes, 2017). The set of PDEs in the model is discretized in time using the Crank-Nicolson method. A Non-linear Full Approximation Scheme is used in the full multigrid V-cycle iterations, and Red-Black ordering is used in the Gauss-Seidel relaxation. A block-structure multilevel Cartesian mesh isused consisting of three global levels with mesh sizes η_−2_ = 40/8, η_−1_ = 40/16, η_0_ = 40/32, and two adaptively refined levels with mesh sizes η_1_ = 40/64, η_2_ = 40/128. A numerical simulation of desmoplastic tumor progression in 3D which shows that the algorithm is capable of simulating an ECM-rich tumor environment and the handling of morphological changes during tumor progression (Ng and Frieboes, 2017).

The diffuse interface model is chosen to bypass the need to track the transient position of sharp interfaces, as well as enforcing complicated boundary conditions across these interfaces, such as those required in a sharp interface model. The adaptive multigrid algorithm used can efficiently handle the narrow transition layers as well as larger morphological evolutions. As opposed to lexicographic ordering, the Red-Black sweep used here in relaxations/error smoothings allows for easy parallelization to minimize the computational expense.

In the future, 3D meshes with more refinement levels and finer grids will be evaluated. Different criteria, such as the relative truncation error test or a simple volume fraction test, will be used to flag cells for refinement, and the minimum block size allowed during block generation will also be reduced. The mathematical model may be augmented to include additional tumor cell species as well as immune cell species, together with their corresponding functions and effects. Tumorigenic species such as hormonal growth factors and chemoattractants could be added. An anticancer drug species may be added to study various tumor responses to different therapeutics. A more expansive role and interaction of the lymphatic system with the microenvironment (e.g., Scianna et al., 2013; Swartz, 2014) could be implemented. The current numerical model is coded in C and is partially parallelized. Full parallelization of the program will be explored to speed up and extend the capabilities of the code to handle models of larger scale.

## Author contributions

HF: study conception and scientific oversight; CN and HF: model definition and design; CN: initial implementation; CN and HF: code debugging; CN and HF: manuscript writing and revision. Both authors reviewed and approved the final manuscript.

### Conflict of interest statement

The authors declare that the research was conducted in the absence of any commercial or financial relationships that could be construed as a potential conflict of interest. The reviewer LP and handling Editor declared their shared affiliation.
