# Supplementary material for: Simulation of Multispecies Desmoplastic Cancer Growth via a Fully Adaptive Non-linear Full Multigrid Algorithm
Source: Front Physiol. 2018 Jul 12;9:821. doi: 10.3389/fphys.2018.00821 (PMC6052761; doi:10.3389/fphys.2018.00821)
Supplement: Supplementary file 1 [file Presentation_1.pdf]

Supplementary Material

Simulation of Multispecies Desmoplastic Cancer Growth via a Fully Adaptive Nonlinear Full Multigrid Algorithm

Chin F. Ng[1], Hermann B. Frieboes[1,2*]

[1]Department of Bioengineering, University of Louisville, Louisville, KY, USA

[2]James Graham Brown Cancer Center, University of Louisville, Louisville, KY, USA

*** Correspondence:** Hermann B. Frieboes hbfrie01@louisville.edu

1 Numerical Methods

1.1 Differential, Laplacian, and Flux Terms

Let all variables be defined on cell centers, except velocities $\tilde{\boldsymbol{u}}_\alpha$ and $\tilde{\boldsymbol{u}}_\beta$, which are defined on edge centers. Spatial derivatives acting on dimensionless scalar $\tilde{f}_{i,j,k}$ and vector $\tilde{\boldsymbol{f}}_{i,j,k}$ functions are replaced by differential operators D_x, D_y, and D_z in the following manner:

$$\nabla_d\left(\tilde{f}_{i,j,k}\right) = D_x\left(\tilde{f}_{i,j,k}\right) + D_y\left(\tilde{f}_{i,j,k}\right) + D_z\left(\tilde{f}_{i,j,k}\right) = \frac{\tilde{f}_{i+\frac{1}{2},j,k} - \tilde{f}_{i-\frac{1}{2},j,k}}{\eta}\boldsymbol{\delta_i} + \frac{\tilde{f}_{i,j+\frac{1}{2},k} - \tilde{f}_{i,j-\frac{1}{2},k}}{\eta}\boldsymbol{\delta_j} + \frac{\tilde{f}_{i,j,k+\frac{1}{2}} - \tilde{f}_{i,j,k-\frac{1}{2}}}{\eta}\boldsymbol{\delta_k} \quad (1.1.1)$$

$$\nabla_d \cdot \left(\tilde{\boldsymbol{f}}_{i,j,k}\right) = D_x\left(\tilde{f}^{\,x}_{i,j,k}\right) + D_y\left(\tilde{f}^{\,y}_{i,j,k}\right) + D_z\left(\tilde{f}^{\,z}_{i,j,k}\right) = \frac{\tilde{f}^{\,x}_{i+\frac{1}{2},j,k} - \tilde{f}^{\,x}_{i-\frac{1}{2},j,k}}{\eta} + \frac{\tilde{f}^{\,y}_{i,j+\frac{1}{2},k} - \tilde{f}^{\,y}_{i,j-\frac{1}{2},k}}{\eta} + \frac{\tilde{f}^{\,z}_{i,j,k+\frac{1}{2}} - \tilde{f}^{\,z}_{i,j,k-\frac{1}{2}}}{\eta} \quad (1.1.2)$$

where i, j, and k in the two equations above include non-integers; $\boldsymbol{\delta_i}$, $\boldsymbol{\delta_j}$, and $\boldsymbol{\delta_k}$ are unit vectors in the $x-$, $y-$, and $z-$ direction, respectively. Following definitions given in Eqs. (1.1.1) & (1.1.2), the discrete Laplacian operator is defined by the second order approximation below:

$$
\begin{aligned}
\Delta_d(\tilde{f}_{i,j,k}) =& \ \nabla_d \cdot [\nabla_d(\tilde{f}_{i,j,k})] \\
&= D_x[\nabla_d(\tilde{f}_{i,j,k})]^x + D_y[\nabla_d(\tilde{f}_{i,j,k})]^y + D_z[\nabla_d(\tilde{f}_{i,j,k})]^z \\
&= \frac{\left[D_x\left(\tilde{f}_{i+\frac{1}{2},j,k}\right)\right]^x - \left[D_x\left(\tilde{f}_{i-\frac{1}{2},j,k}\right)\right]^x}{\eta} + \frac{\left[D_y\left(\tilde{f}_{i,j+\frac{1}{2},k}\right)\right]^y - \left[D_y\left(\tilde{f}_{i,j-\frac{1}{2},k}\right)\right]^y}{\eta} \\
&\quad + \frac{\left[D_z\left(\tilde{f}_{i,j,k+\frac{1}{2}}\right)\right]^z - \left[D_z\left(\tilde{f}_{i,j,k-\frac{1}{2}}\right)\right]^z}{\eta} \\
&= \frac{1}{\eta}\left[\left(\frac{\tilde{f}_{i+1,j,k} - \tilde{f}_{i,j,k}}{\eta}\right) - \left(\frac{\tilde{f}_{i,j,k} - \tilde{f}_{i-1,j,k}}{\eta}\right)\right] \\
&\quad + \frac{1}{\eta}\left[\left(\frac{\tilde{f}_{i,j+1,k} - \tilde{f}_{i,j,k}}{\eta}\right) - \left(\frac{\tilde{f}_{i,j,k} - \tilde{f}_{i,j-1,k}}{\eta}\right)\right] \\
&\quad + \frac{1}{\eta}\left[\left(\frac{\tilde{f}_{i,j,k+1} - \tilde{f}_{i,j,k}}{\eta}\right) - \left(\frac{\tilde{f}_{i,j,k} - \tilde{f}_{i,j,k-1}}{\eta}\right)\right] \\
&= \frac{\tilde{f}_{i-1,j,k} + \tilde{f}_{i,j-1,k} + \tilde{f}_{i,j,k-1} - 6\tilde{f}_{i,j,k} + \tilde{f}_{i+1,j,k} + \tilde{f}_{i,j+1,k} + \tilde{f}_{i,j,k+1}}{\eta^2}
\end{aligned} \tag{1.1.3}
$$

Using the above definitions, Laplacian terms in Eqs. (2.1) – (2.3) can be approximated by

$$
\begin{aligned}
&\nabla_d \cdot [(\widetilde{M}_{i,j,k})\,\nabla_d(\tilde{\mu}_{i,j,k})] \\
&= \frac{1}{\eta^2}\left[A_x\left(\widetilde{M}_{i+\frac{1}{2},j,k}\right)(\tilde{\mu}_{i+1,j,k} - \tilde{\mu}_{i,j,k}) - A_x\left(\widetilde{M}_{i-\frac{1}{2},j,k}\right)(\tilde{\mu}_{i,j,k} - \tilde{\mu}_{i-1,j,k})\right] \\
&\quad + \frac{1}{\eta^2}\left[A_y\left(\widetilde{M}_{i,j+\frac{1}{2},k}\right)(\tilde{\mu}_{i,j+1,k} - \tilde{\mu}_{i,j,k}) - A_y\left(\widetilde{M}_{i,j-\frac{1}{2},k}\right)(\tilde{\mu}_{i,j,k} - \tilde{\mu}_{i,j-1,k})\right] \\
&\quad + \frac{1}{\eta^2}\left[A_z\left(\widetilde{M}_{i,j,k+\frac{1}{2}}\right)(\tilde{\mu}_{i,j,k+1} - \tilde{\mu}_{i,j,k}) - A_z\left(\widetilde{M}_{i,j,k-\frac{1}{2}}\right)(\tilde{\mu}_{i,j,k} - \tilde{\mu}_{i,j,k-1})\right] \\
&= \frac{1}{\eta^2}\Big\{A_x\left(\widetilde{M}_{i+\frac{1}{2},j,k}\right)\tilde{\mu}_{i+1,j,k} + A_x\left(\widetilde{M}_{i-\frac{1}{2},j,k}\right)\tilde{\mu}_{i-1,j,k} + A_y\left(\widetilde{M}_{i,j+\frac{1}{2},k}\right)\tilde{\mu}_{i,j+1,k} \\
&\quad + A_y\left(\widetilde{M}_{i,j-\frac{1}{2},k}\right)\tilde{\mu}_{i,j-1,k} + A_z\left(\widetilde{M}_{i,j,k+\frac{1}{2}}\right)\tilde{\mu}_{i,j,k+1} + A_z\left(\widetilde{M}_{i,j,k-\frac{1}{2}}\right)\tilde{\mu}_{i,j,k-1} \\
&\quad - \tilde{\mu}_{i,j,k}\Big[A_x\left(\widetilde{M}_{i+\frac{1}{2},j,k}\right) + A_x\left(\widetilde{M}_{i-\frac{1}{2},j,k}\right) + A_y\left(\widetilde{M}_{i,j+\frac{1}{2},k}\right) + A_y\left(\widetilde{M}_{i,j-\frac{1}{2},k}\right) \\
&\quad + A_z\left(\widetilde{M}_{i,j,k+\frac{1}{2}}\right) + A_z\left(\widetilde{M}_{i,j,k-\frac{1}{2}}\right)\Big]\Big\}
\end{aligned} \tag{1.1.4}
$$

where edge-centered approximation of cell-centered variables may be determined by the following averaging operators A:

$$A_x\left(\widetilde{M}_{i\pm\frac{1}{2},j,k}\right) = \frac{\widetilde{M}_{i\pm1,j,k} + \widetilde{M}_{i,j,k}}{2}$$
$$A_y\left(\widetilde{M}_{i,j\pm\frac{1}{2},k}\right) = \frac{\widetilde{M}_{i,j\pm1,k} + \widetilde{M}_{i,j,k}}{2} \qquad (1.1.5)$$
$$A_z\left(\widetilde{M}_{i,j,k\pm\frac{1}{2}}\right) = \frac{\widetilde{M}_{i,j,k\pm1} + \widetilde{M}_{i,j,k}}{2}$$

Note that the averaging operators above also apply to cell-centered approximation of edge-centered variables.

Similarly, the convective flux terms in Eqs. (2.1) – (2.3) and Eqs. (2.33) – (2.35) are approximated by

$$\begin{aligned}\nabla_d \cdot \left[\left(\tilde{\phi}_{i,j,k}\right)\left(\tilde{\boldsymbol{u}}_{i,j,k}\right)\right] = &\frac{1}{\eta}\left[W_x\left(\tilde{\phi}_{i+\frac{1}{2},j,k}\right)\left(\tilde{u}^x_{i+\frac{1}{2},j,k}\right) - W_x\left(\tilde{\phi}_{i-\frac{1}{2},j,k}\right)\left(\tilde{u}^x_{i-\frac{1}{2},j,k}\right)\right] \\ &+ \frac{1}{\eta}\left[W_y\left(\tilde{\phi}_{i,j+\frac{1}{2},k}\right)\left(\tilde{u}^y_{i,j+\frac{1}{2},k}\right) - W_y\left(\tilde{\phi}_{i,j-\frac{1}{2},k}\right)\left(\tilde{u}^y_{i,j-\frac{1}{2},k}\right)\right] \\ &+ \frac{1}{\eta}\left[W_z\left(\tilde{\phi}_{i,j,k+\frac{1}{2}}\right)\left(\tilde{u}^z_{i,j,k+\frac{1}{2}}\right) - W_z\left(\tilde{\phi}_{i,j,k-\frac{1}{2}}\right)\left(\tilde{u}^z_{i,j,k-\frac{1}{2}}\right)\right]\end{aligned} \qquad (1.1.6)$$

where $\tilde{u}^x$, $\tilde{u}^y$, and $\tilde{u}^z$ are edge-centered velocity components. The edge-centered approximations of cell-centered $\tilde{\phi}$ can be computed using the upwind biased WENO scheme (1, 2):

$$W_x\left(\tilde{\phi}_{i\pm\frac{1}{2},j,k}\right) = \frac{-\tilde{\phi}_{\left(i\pm\frac{1}{2}\right)-\frac{3}{2},j,k} + 5\,\tilde{\phi}_{\left(i\pm\frac{1}{2}\right)-\frac{1}{2},j,k} + 2\,\tilde{\phi}_{\left(i\pm\frac{1}{2}\right)+\frac{1}{2},j,k}}{6}$$
$$W_y\left(\tilde{\phi}_{i,j\pm\frac{1}{2},k}\right) = \frac{-\tilde{\phi}_{i,\left(j\pm\frac{1}{2}\right)-\frac{3}{2},k} + 5\,\tilde{\phi}_{i,\left(j\pm\frac{1}{2}\right)-\frac{1}{2},k} + 2\,\tilde{\phi}_{i,\left(j\pm\frac{1}{2}\right)+\frac{1}{2},k}}{6} \qquad (1.1.7)$$
$$W_z\left(\tilde{\phi}_{i,j,k\pm\frac{1}{2}}\right) = \frac{-\tilde{\phi}_{i,j,\left(k\pm\frac{1}{2}\right)-\frac{3}{2}} + 5\,\tilde{\phi}_{i,j,\left(k\pm\frac{1}{2}\right)-\frac{1}{2}} + 2\,\tilde{\phi}_{i,j,\left(k\pm\frac{1}{2}\right)+\frac{1}{2}}}{6}$$

In our case, a simple upwind Donor-Cell advection is used to estimate edge-centered values from cell-centered data.

1.2 Discretized Governing Equations

The model consists of a set of stiff differential equations that are fourth-order in space. At time step a with time step size θ, they are discretized in time using the Crank-Nicolson Method as in Wise, Lowengrub (3) (terms computed from the converged solution of the previous time step are in blue).

Cells and ECM Components

$$\left(\tilde{\phi}_V\right)_{i,j,k}^{a} - \frac{\theta}{2}\left[\nabla\cdot\left(\tilde{M}_V\nabla\tilde{\mu}_T\right)_{i,j,k}^{a}\right] + \frac{\theta}{2}\left[\nabla\cdot\left(\tilde{\boldsymbol{u}}_\alpha\tilde{\phi}_V\right)_{i,j,k}^{a}\right] - \frac{\theta}{2}\left[\left(\tilde{S}_V\right)_{i,j,k}^{a}\right] = \left(\tilde{\phi}_V\right)_{i,j,k}^{a-1} + \frac{\theta}{2}\left[\nabla\cdot\left(\tilde{M}_V\nabla\tilde{\mu}_T\right)_{i,j,k}^{a-1}\right] - \frac{\theta}{2}\left[\nabla\cdot\left(\tilde{\boldsymbol{u}}_\alpha\tilde{\phi}_V\right)_{i,j,k}^{a-1}\right] + \frac{\theta}{2}\left[\left(\tilde{S}_V\right)_{i,j,k}^{a-1}\right] \quad (1.2.1)$$

$$\left(\tilde{\phi}_D\right)_{i,j,k}^{a} - \frac{\theta}{2}\left[\nabla\cdot\left(\tilde{M}_D\nabla\tilde{\mu}_T\right)_{i,j,k}^{a}\right] + \frac{\theta}{2}\left[\nabla\cdot\left(\tilde{\boldsymbol{u}}_\alpha\tilde{\phi}_D\right)_{i,j,k}^{a}\right] - \frac{\theta}{2}\left[\left(\tilde{S}_D\right)_{i,j,k}^{a}\right] = \left(\tilde{\phi}_D\right)_{i,j,k}^{a-1} + \frac{\theta}{2}\left[\nabla\cdot\left(\tilde{M}_D\nabla\tilde{\mu}_T\right)_{i,j,k}^{a-1}\right] - \frac{\theta}{2}\left[\nabla\cdot\left(\tilde{\boldsymbol{u}}_\alpha\tilde{\phi}_D\right)_{i,j,k}^{a-1}\right] + \frac{\theta}{2}\left[\left(\tilde{S}_D\right)_{i,j,k}^{a-1}\right] \quad (1.2.2)$$

$$\left(\tilde{\phi}_E\right)_{i,j,k}^{a} - \frac{\theta}{2}\left[\nabla\cdot\left(\tilde{M}_E\nabla\tilde{\mu}_E\right)_{i,j,k}^{a}\right] + \frac{\theta}{2}\left[\nabla\cdot\left(\tilde{\boldsymbol{u}}_\alpha\tilde{\phi}_E\right)_{i,j,k}^{a}\right] - \frac{\theta}{2}\left[\left(\tilde{S}_E\right)_{i,j,k}^{a}\right] = \left(\tilde{\phi}_E\right)_{i,j,k}^{a-1} + \frac{\theta}{2}\left[\nabla\cdot\left(\tilde{M}_E\nabla\tilde{\mu}_E\right)_{i,j,k}^{a-1}\right] - \frac{\theta}{2}\left[\nabla\cdot\left(\tilde{\boldsymbol{u}}_\alpha\tilde{\phi}_E\right)_{i,j,k}^{a-1}\right] + \frac{\theta}{2}\left[\left(\tilde{S}_E\right)_{i,j,k}^{a-1}\right] \quad (1.2.3)$$

Chemical Potentials

$$(\tilde{\mu}_T)_{i,j,k}^{a} = \left(\frac{\partial\tilde{F}_b}{\partial\tilde{\phi}_T}\right)_{i,j,k}^{a} - \tilde{\epsilon}_T^{\,2}\,\nabla^2\left[\left(\tilde{\phi}_T\right)_{i,j,k}^{a}\right] - \tilde{\epsilon}_{TE}^{\,2}\,\nabla^2\left[\left(\tilde{\phi}_E\right)_{i,j,k}^{a}\right] \quad (1.2.4)$$

$$(\tilde{\mu}_E)_{i,j,k}^{a} = \left(\frac{\partial\tilde{F}_b}{\partial\tilde{\phi}_E}\right)_{i,j,k}^{a} + \left(\frac{\partial\tilde{W}}{\partial\tilde{\phi}_E}\right)_{i,j,k}^{a} - \tilde{\epsilon}_E^{\,2}\,\nabla^2\left[\left(\tilde{\phi}_E\right)_{i,j,k}^{a}\right] - \tilde{\epsilon}_{TE}^{\,2}\,\nabla^2\left[\left(\tilde{\phi}_T\right)_{i,j,k}^{a}\right] \quad (1.2.5)$$

Pressures and Velocities

$$\nabla\cdot\left[\tilde{k}_\alpha\left(\tilde{\phi}_T,\tilde{\phi}_E\right)_{i,j,k}^{a}\nabla(\tilde{p})_{i,j,k}^{a}\right] + \left(\tilde{S}_V + \tilde{S}_D + \tilde{S}_E\right)_{i,j,k}^{a} = \frac{\tilde{\gamma}_T}{\tilde{\epsilon}_T}\nabla\cdot\left[\tilde{k}_\alpha\left(\tilde{\phi}_T,\tilde{\phi}_E\right)_{ijk}^{a-1}(\tilde{\mu}_T)_{i,j,k}^{a-1}\nabla\left(\tilde{\phi}_T\right)_{i,j,k}^{a-1}\right] + \frac{\tilde{\gamma}_E}{\tilde{\epsilon}_E}\nabla\cdot\left[\tilde{k}_\alpha\left(\tilde{\phi}_T,\tilde{\phi}_E\right)_{ijk}^{a-1}(\tilde{\mu}_E)_{i,j,k}^{a-1}\nabla\left(\tilde{\phi}_E\right)_{i,j,k}^{a-1}\right] \quad (1.2.6)$$

$$\nabla^2(\tilde{q})_{i,j,k}^{a} = \frac{R_{\alpha,\beta}}{\tilde{k}_\beta}\left(\tilde{S}_V + \tilde{S}_D + \tilde{S}_E\right)_{i,j,k}^{a} \quad (1.2.7)$$

$$(\tilde{\boldsymbol{u}}_\alpha)_{i,j,k}^{a} = -\tilde{k}_\alpha\left[\nabla(\tilde{p})_{i,j,k}^{a} - \frac{\tilde{\gamma}_T}{\tilde{\epsilon}_T}(\tilde{\mu}_T)_{i,j,k}^{a}\nabla\left(\tilde{\phi}_T\right)_{i,j,k}^{a} - \frac{\tilde{\gamma}_E}{\tilde{\epsilon}_E}(\tilde{\mu}_E)_{i,j,k}^{a}\nabla\left(\tilde{\phi}_E\right)_{i,j,k}^{a}\right] \quad (1.2.8)$$

$$\left(\tilde{\boldsymbol{u}}_\beta\right)_{i,j,k}^{a} = -\,\tilde{k}_\beta\nabla(\tilde{q})_{i,j,k}^{a} \quad (1.2.9)$$

$$(\tilde{\boldsymbol{u}}_E)_{i,j,k}^{a} = \tilde{\boldsymbol{u}}_\alpha - \tilde{M}\nabla(\tilde{\mu}_E)_{i,j,k}^{a} \quad (1.2.10)$$

Nutrients and Waste Products

$$0 = \nabla\cdot\left(\tilde{D}_n\nabla\tilde{n}\right)_{i,j,k}^{a} + \tilde{n}_C\left(\tilde{k}_{n1}\right)_{i,j,k}^{a} - \left[\left(\tilde{k}_{n1}+\tilde{k}_{n2}\right)\tilde{n}\right]_{i,j,k}^{a} \tag{1.2.11}$$

$$0 = \nabla\cdot\left(\tilde{D}_g\nabla\tilde{g}\right)_{i,j,k}^{a} + \tilde{g}_C\left(\tilde{k}_{g1}\right)_{i,j,k}^{a} - \left[\left(\tilde{k}_{g1}+\tilde{k}_{g2}\right)\tilde{g}\right]_{i,j,k}^{a} \tag{1.2.12}$$

$$0 = \nabla\cdot\left(\tilde{D}_w\nabla\tilde{w}\right)_{i,j,k}^{a} + \left[\tilde{k}_{n2}\tilde{n} + \tilde{k}_r\tilde{b}\tilde{a} + \tilde{k}_w\tilde{w}_C\right]_{i,j,k}^{a} - \left(\tilde{k}_f+\tilde{k}_w\right)_{i,j,k}^{a}(\tilde{w})_{i,j,k}^{a} \tag{1.2.13}$$

$$\begin{aligned} 0 = &-\tilde{z}_\ell\nabla\cdot\left[\tilde{D}_\ell\,\tilde{\ell}\left(\frac{\tilde{z}_\ell\tilde{D}_\ell\nabla\tilde{\ell}+\tilde{z}_b\tilde{D}_b\nabla\tilde{b}+\tilde{D}_a\nabla\tilde{a}+\tilde{z}_s\tilde{D}_s\nabla\tilde{s}+\tilde{z}_r\tilde{D}_r\nabla\tilde{r}}{\tilde{z}_\ell^2\,\tilde{D}_\ell\,\tilde{\ell}+\tilde{z}_b^2\,\tilde{D}_b\,\tilde{b}+\tilde{D}_a\,\tilde{a}+\tilde{z}_s^2\,\tilde{D}_s\,\tilde{s}+\tilde{z}_r^2\,\tilde{D}_r\,\tilde{r}}\right)\right]_{i,j,k}^{a} \\ &+\nabla\cdot\left(\tilde{D}_\ell\nabla\tilde{\ell}\right)_{i,j,k}^{a} + 2R_{g,n}\left(\tilde{k}_{g2}\tilde{g}\right)_{i,j,k}^{a} - \frac{1}{3}\left(\tilde{k}_{n2}\tilde{n}\right)_{i,j,k}^{a} + \tilde{\ell}_C\left(\tilde{k}_\ell\right)_{i,j,k}^{a} \\ &-\left(\tilde{k}_\ell\tilde{\ell}\right)_{i,j,k}^{a} \end{aligned} \tag{1.2.14}$$

$$\begin{aligned} 0 = &-\tilde{z}_b\nabla\cdot\left[\tilde{D}_b\tilde{b}\left(\frac{\tilde{z}_\ell\tilde{D}_\ell\nabla\tilde{\ell}+\tilde{z}_b\tilde{D}_b\nabla\tilde{b}+\tilde{D}_a\nabla\tilde{a}+\tilde{z}_s\tilde{D}_s\nabla\tilde{s}+\tilde{z}_r\tilde{D}_r\nabla\tilde{r}}{\tilde{z}_\ell^2\,\tilde{D}_\ell\,\tilde{\ell}+\tilde{z}_b^2\,\tilde{D}_b\,\tilde{b}+\tilde{D}_a\,\tilde{a}+\tilde{z}_s^2\,\tilde{D}_s\,\tilde{s}+\tilde{z}_r^2\,\tilde{D}_r\,\tilde{r}}\right)\right]_{i,j,k}^{a} \\ &+\nabla\cdot\left(\tilde{D}_b\nabla\tilde{b}\right)_{i,j,k}^{a} + \left(\tilde{k}_f\tilde{w}\right)_{i,j,k}^{a} - \left(\tilde{k}_r\tilde{b}\tilde{a}\right)_{i,j,k}^{a} \end{aligned} \tag{1.2.15}$$

$$\begin{aligned} 0 = &-\tilde{z}_a\nabla\cdot\left[\tilde{D}_a\tilde{a}\left(\frac{\tilde{z}_\ell\tilde{D}_\ell\nabla\tilde{\ell}+\tilde{z}_b\tilde{D}_b\nabla\tilde{b}+\tilde{D}_a\nabla\tilde{a}+\tilde{z}_s\tilde{D}_s\nabla\tilde{s}+\tilde{z}_r\tilde{D}_r\nabla\tilde{r}}{\tilde{z}_\ell^2\,\tilde{D}_\ell\,\tilde{\ell}+\tilde{z}_b^2\,\tilde{D}_b\,\tilde{b}+\tilde{D}_a\,\tilde{a}+\tilde{z}_s^2\,\tilde{D}_s\,\tilde{s}+\tilde{z}_r^2\,\tilde{D}_r\,\tilde{r}}\right)\right]_{i,j,k}^{a} \\ &+\nabla\cdot\left(\tilde{D}_a\nabla\tilde{a}\right)_{i,j,k}^{a} + 2R_{g,n}\left(\tilde{k}_{g2}\tilde{g}\right)_{i,j,k}^{a} - \frac{1}{3}\left(\tilde{k}_{n2}\tilde{n}\right)_{i,j,k}^{a} + \tilde{k}_f(\tilde{w})_{i,j,k}^{a} \\ &-\tilde{k}_r\left(\tilde{b}\tilde{a}\right)_{i,j,k}^{a} + \tilde{\ell}_C\left(\tilde{k}_\ell\right)_{i,j,k}^{a} - \left(\tilde{k}_\ell\tilde{\ell}\right)_{i,j,k}^{a} \end{aligned} \tag{1.2.16}$$

$$\begin{aligned} 0 = &-\tilde{z}_s\,\nabla\cdot\left[\tilde{D}_s\tilde{s}\left(\frac{\tilde{z}_\ell\tilde{D}_\ell\nabla\tilde{\ell}+\tilde{z}_b\tilde{D}_b\nabla\tilde{b}+\tilde{D}_a\nabla\tilde{a}+\tilde{z}_s\tilde{D}_s\nabla\tilde{s}+\tilde{z}_r\tilde{D}_r\nabla\tilde{r}}{\tilde{z}_\ell^2\,\tilde{D}_\ell\,\tilde{\ell}+\tilde{z}_b^2\,\tilde{D}_b\,\tilde{b}+\tilde{D}_a\,\tilde{a}+\tilde{z}_s^2\,\tilde{D}_s\,\tilde{s}+\tilde{z}_r^2\,\tilde{D}_r\,\tilde{r}}\right)\right]_{i,j,k}^{a} \\ &+\nabla\cdot\left(\tilde{D}_s\nabla\tilde{s}\right)_{i,j,k}^{a} \end{aligned} \tag{1.2.17}$$

$$(\tilde{r})_{i,j,k}^{a} = -\frac{1}{\tilde{z}_r}\left[\tilde{z}_\ell\left(\tilde{\ell}\right)_{i,j,k}^{a} + \tilde{z}_b\left(\tilde{b}\right)_{i,j,k}^{a} + \tilde{z}_a(\tilde{a})_{i,j,k}^{a} + \tilde{z}_s(\tilde{s})_{i,j,k}^{a}\right] \tag{1.2.18}$$

Tumorigenic Species

$$0 = \nabla\cdot\left(\tilde{D}_{tgf}\nabla\widetilde{tgf}\right)_{i,j,k}^{a} + \left(\tilde{\lambda}_{tgf}\right)_{i,j,k}^{a} - \left(\tilde{\lambda}_{tgf}+\tilde{\lambda}_{de,tgf}+\tilde{\lambda}_{U,tgf}\right)_{i,j,k}^{a}\left(\widetilde{tgf}\right)_{i,j,k}^{a} \tag{1.2.19}$$

$$0 = \nabla\cdot\left(\tilde{D}_{taf}\nabla\widetilde{taf}\right)_{i,j,k}^{a} + \left(\tilde{\lambda}_{taf}\right)_{i,j,k}^{a} - \left(\tilde{\lambda}_{taf}+\tilde{\lambda}_{de,taf}+\tilde{\lambda}_{U,taf}\right)_{i,j,k}^{a}\left(\widetilde{taf}\right)_{i,j,k}^{a} \tag{1.2.20}$$

$$\begin{aligned} &(\tilde{m})_{i,j,k}^{a} - \frac{\theta}{2}\left[\nabla\cdot\left(\tilde{D}_m\nabla\tilde{m}\right)_{i,j,k}^{a} + \left(\tilde{S}_m\right)_{i,j,k}^{a}\right] \\ &= (\tilde{m})_{i,j,k}^{a-1} + \frac{\theta}{2}\left[\nabla\cdot\left(\tilde{D}_m\nabla\tilde{m}\right)_{i,j,k}^{a-1} + \left(\tilde{S}_m\right)_{i,j,k}^{a-1}\right] \end{aligned} \tag{1.2.21}$$

$$\left(\tilde{F}_E\right)_{i,j,k}^{a} + \frac{\theta}{2}\left[\nabla\cdot\left(\tilde{F}_E\tilde{\boldsymbol{u}}_E\right)_{i,j,k}^{a} + \nabla\cdot\left(\tilde{D}_F\tilde{F}_E\nabla\widetilde{tgf}\right)_{i,j,k}^{a} - \left(\tilde{S}_{FE}\right)_{i,j,k}^{a}\right]$$
$$= \left(\tilde{F}_E\right)_{i,j,k}^{a-1} - \frac{\theta}{2}\left[\nabla\cdot\left(\tilde{F}_E\tilde{\boldsymbol{u}}_E\right)_{i,j,k}^{a-1} + \nabla\cdot\left(\tilde{D}_F\tilde{F}_E\nabla\widetilde{tgf}\right)_{i,j,k}^{a-1} - \left(\tilde{S}_{FE}\right)_{i,j,k}^{a-1}\right] \quad (1.2.22)$$

Blood and Lymphatic Vessels

$$\left(\tilde{B}_n^E\right)_{i,j,k}^{a} + \frac{\theta}{2}\Big\{\nabla\cdot\left(\tilde{B}_n^E\tilde{\boldsymbol{u}}_E\right)_{i,j,k}^{a} + \nabla\cdot\left[\tilde{\chi}_{che,BnE}\left(\mathcal{A}_{che,BnE}\tilde{B}_n^E\nabla\widetilde{taf}\right)_{i,j,k}^{a}\right]$$
$$+ \nabla\cdot\left[\tilde{\chi}_{hap,BnE}\left(\mathcal{A}_{hap,BnE}\tilde{B}_n^E\nabla\tilde{\phi}_E\right)_{i,j,k}^{a}\right] - \nabla\cdot\left(\tilde{D}_{BnE}\nabla\tilde{B}_n^E\right)_{i,j,k}^{a} - \left(\tilde{S}_{BnE}\right)_{i,j,k}^{a}\Big\}$$
$$= \left(\tilde{B}_n^E\right)_{i,j,k}^{a-1} - \frac{\theta}{2}\Big\{\nabla\cdot\left(\tilde{B}_n^E\tilde{\boldsymbol{u}}_E\right)_{i,j,k}^{a-1} - \left(\tilde{S}_{BnE}\right)_{i,j,k}^{a-1} + \nabla\cdot\left[\tilde{\chi}_{che,BnE}\left(\mathcal{A}_{che,BnE}\tilde{B}_n^E\nabla\widetilde{taf}\right)_{i,j,k}^{a-1}\right] \quad (1.2.23)$$
$$+ \nabla\cdot\left[\tilde{\chi}_{hap,BnE}\left(\mathcal{A}_{hap,BnE}\tilde{B}_n^E\nabla\tilde{\phi}_E\right)_{i,j,k}^{a-1}\right] - \nabla\cdot\left(\tilde{D}_{BnE}\nabla\tilde{B}_n^E\right)_{i,j,k}^{a-1}\Big\}$$

$$\left(\tilde{L}_n^E\right)_{i,j,k}^{a} + \frac{\theta}{2}\Big\{\nabla\cdot\left(\tilde{L}_n^E\tilde{\boldsymbol{u}}_E\right)_{i,j,k}^{a} + \nabla\cdot\left[\tilde{\chi}_{che,LnE}\left(\mathcal{A}_{che,LnE}\tilde{L}_n^E\nabla\widetilde{taf}\right)_{i,j,k}^{a}\right]$$
$$+ \nabla\cdot\left[\tilde{\chi}_{hap,LnE}\left(\mathcal{A}_{hap,LnE}\tilde{L}_n^E\nabla\tilde{\phi}_E\right)_{i,j,k}^{a}\right] - \nabla\cdot\left(\tilde{D}_{LnE}\nabla\tilde{L}_n^E\right)_{i,j,k}^{a} - \left(\tilde{S}_{LnE}\right)_{i,j,k}^{a}\Big\}$$
$$= \left(\tilde{L}_n^E\right)_{i,j,k}^{a-1} - \frac{\theta}{2}\Big\{\nabla\cdot\left(\tilde{L}_n^E\tilde{\boldsymbol{u}}_E\right)_{i,j,k}^{a-1} + \nabla\cdot\left[\tilde{\chi}_{che,LnE}\left(\mathcal{A}_{che,LnE}\tilde{L}_n^E\nabla\widetilde{taf}\right)_{i,j,k}^{a-1}\right] \quad (1.2.24)$$
$$+ \nabla\cdot\left[\tilde{\chi}_{hap,LnE}\left(\mathcal{A}_{hap,LnE}\tilde{L}_n^E\nabla\tilde{\phi}_E\right)_{i,j,k}^{a-1}\right] - \nabla\cdot\left(\tilde{D}_{LnE}\nabla\tilde{L}_n^E\right)_{i,j,k}^{a-1} - \left(\tilde{S}_{LnE}\right)_{i,j,k}^{a-1}\Big\}$$

Extraction of the strain tensor from the set of elastic energy equations shown in Eqs. (2.10) – (2.16) is discussed in the next section.

1.3 Multigrid V–Cycle Iterations

Terms at current time step a in equation sets in the previous secction are divided according to their iteration sequence. Each iteration travels through one V–cycle, starting from the finest mesh level (see also Adaptive FAS V-Cycle in **Results**). Letting the iteration number be r, we rewrite all governing equations and group all known terms entering each V–cycle to the RHS of equations in the following manner (terms computed from the previous iteration of the current time step are in green):

Cells and ECM Components

$$\left(\tilde{\phi}_V\right)_{i,j,k}^{a,r} - \frac{\theta}{2}\left[\nabla\cdot\left(\tilde{M}_V\nabla\tilde{\mu}_T\right)_{i,j,k}^{a,r}\right]$$
$$= \left(\tilde{\phi}_V\right)_{i,j,k}^{a-1} + \frac{\theta}{2}\left[\nabla\cdot\left(\tilde{M}_V\nabla\tilde{\mu}_T\right)_{i,j,k}^{a-1} - \nabla\cdot\left(\tilde{\boldsymbol{u}}_\alpha\tilde{\phi}_V\right)_{i,j,k}^{a-1} + \left(\tilde{S}_V\right)_{i,j,k}^{a-1}\right] \quad (1.3.1)$$
$$+ \frac{\theta}{2}\left[\left(\tilde{S}_V\right)_{i,j,k}^{a,r-1} - \nabla\cdot\left(\tilde{\boldsymbol{u}}_\alpha\tilde{\phi}_V\right)_{i,j,k}^{a,r-1}\right]$$

$$(\tilde{\phi}_D)^{a,r}_{i,j,k} - \frac{\theta}{2}\left[\nabla\cdot\left(\widetilde{M}_D\nabla\tilde{\mu}_T\right)^{a,r}_{i,j,k}\right]$$
$$= (\tilde{\phi}_D)^{a-1}_{i,j,k} + \frac{\theta}{2}\left[\nabla\cdot\left(\widetilde{M}_D\nabla\tilde{\mu}_T\right)^{a-1}_{i,j,k} - \nabla\cdot\left(\tilde{\boldsymbol{u}}_\alpha\tilde{\phi}_D\right)^{a-1}_{i,j,k} + \left(\tilde{S}_D\right)^{a-1}_{i,j,k}\right]$$
$$+ \frac{\theta}{2}\left[\left(\tilde{S}_D\right)^{a,r-1}_{i,j,k} - \nabla\cdot\left(\tilde{\boldsymbol{u}}_\alpha\tilde{\phi}_D\right)^{a,r-1}_{i,j,k}\right] \tag{1.3.2}$$

$$(\tilde{\phi}_E)^{a,r}_{i,j,k} - \frac{\theta}{2}\left[\nabla\cdot\left(\widetilde{M}_E\nabla\tilde{\mu}_E\right)^{a,r}_{i,j,k}\right]$$
$$= (\tilde{\phi}_E)^{a-1}_{i,j,k} + \frac{\theta}{2}\left[\nabla\cdot\left(\widetilde{M}_E\nabla\tilde{\mu}_E\right)^{a-1}_{i,j,k} - \nabla\cdot\left(\tilde{\boldsymbol{u}}_\alpha\tilde{\phi}_E\right)^{a-1}_{i,j,k} + \left(\tilde{S}_E\right)^{a-1}_{i,j,k}\right]$$
$$+ \frac{\theta}{2}\left[\left(\tilde{S}_E\right)^{a,r-1}_{i,j,k} - \nabla\cdot\left(\tilde{\boldsymbol{u}}_\alpha\tilde{\phi}_E\right)^{a,r-1}_{i,j,k}\right] \tag{1.3.3}$$

Chemical Potentials

$$(\tilde{\mu}_T)^{a,r}_{i,j,k} - \left(\frac{\partial\tilde{F}_b}{\partial\tilde{\phi}_T}\right)^{a,r}_{i,j,k} + \tilde{\epsilon}_T{}^2\,\nabla^2\left(\tilde{\phi}_T\right)^{a,r}_{i,j,k} + \tilde{\epsilon}_{TE}{}^2\,\nabla^2\left(\tilde{\phi}_E\right)^{a,r}_{i,j,k} = 0 \tag{1.3.4}$$

$$(\tilde{\mu}_E)^{a,r}_{i,j,k} - \left(\frac{\partial\tilde{F}_b}{\partial\tilde{\phi}_E}\right)^{a,r}_{i,j,k} - \left(\frac{\partial\widetilde{\mathcal{W}}}{\partial\tilde{\phi}_E}\right)^{a,r}_{i,j,k} + \tilde{\epsilon}_E{}^2\,\nabla^2\left[\left(\tilde{\phi}_E\right)^{a,r}_{i,j,k}\right] + \tilde{\epsilon}_{TE}{}^2\,\nabla^2\left[\left(\tilde{\phi}_T\right)^{a,r}_{i,j,k}\right] = 0 \tag{1.3.5}$$

Pressures and Velocities

$$\nabla\cdot\left[\tilde{k}_\alpha\left(\tilde{\phi}_T,\tilde{\phi}_E\right)^{a,r-1}_{i,j,k}\nabla(\tilde{p})^{a,r}_{i,j,k}\right] = \frac{\tilde{\gamma}_T}{\tilde{\epsilon}_T}\nabla\cdot\left[\tilde{k}_\alpha\left(\tilde{\phi}_T,\tilde{\phi}_E\right)^{a-1}_{ijk}(\tilde{\mu}_T)^{a-1}_{i,j,k}\nabla\left(\tilde{\phi}_T\right)^{a-1}_{i,j,k}\right]$$
$$+\frac{\tilde{\gamma}_E}{\tilde{\epsilon}_E}\nabla\cdot\left[\tilde{k}_\alpha\left(\tilde{\phi}_T,\tilde{\phi}_E\right)^{a-1}_{ijk}(\tilde{\mu}_E)^{a-1}_{i,j,k}\nabla\left(\tilde{\phi}_E\right)^{a-1}_{i,j,k}\right]$$
$$-\left(\tilde{S}_V+\tilde{S}_D+\tilde{S}_E\right)^{a,r-1}_{i,j,k} \tag{1.3.6}$$

$$\nabla^2(\tilde{q})^{a,r}_{i,j,k} = \frac{R_{\alpha,\beta}}{\tilde{k}_\beta}\left(\tilde{S}_V+\tilde{S}_D+\tilde{S}_E\right)^{a,r-1}_{i,j,k} \tag{1.3.7}$$

$$(\tilde{\boldsymbol{u}}_\alpha)^{a,r}_{i,j,k} = -\tilde{k}_\alpha\left[\nabla(\tilde{p})^{a,r}_{i,j,k} - \frac{\tilde{\gamma}_T}{\tilde{\epsilon}_T}(\tilde{\mu}_T)^{a,r}_{i,j,k}\nabla\left(\tilde{\phi}_T\right)^{a,r}_{i,j,k} - \frac{\tilde{\gamma}_E}{\tilde{\epsilon}_E}(\tilde{\mu}_E)^{a,r}_{i,j,k}\nabla\left(\tilde{\phi}_E\right)^{a,r}_{i,j,k}\right] \tag{1.3.8}$$

$$\left(\tilde{\boldsymbol{u}}_\beta\right)^{a,r}_{i,j,k} = -\,\tilde{k}_\beta\nabla(\tilde{q})^{a,r}_{i,j,k} \tag{1.3.9}$$

$$(\tilde{\boldsymbol{u}}_E)^{a,r}_{i,j,k} = (\tilde{\boldsymbol{u}}_\alpha)^{a,r}_{i,j,k} - \widetilde{M}\nabla(\tilde{\mu}_E)^{a,r}_{i,j,k} \tag{1.3.10}$$

Nutrients and Waste Products

$$\nabla\cdot\left(\widetilde{D}_n{}^{a,r-1}\,\nabla\,\tilde{n}^{a,r}\right)_{i,j,k} - \left(\tilde{k}_{n1}+\tilde{k}_{n2}\right)^{a,r-1}_{i,j,k}(\tilde{n})^{a,r}_{i,j,k} = -\,\tilde{n}_C\left(\tilde{k}_{n1}\right)^{a,r-1}_{i,j,k} \tag{1.3.11}$$

$$\nabla\cdot\left(\widetilde{D}_g{}^{a,r-1}\,\nabla\,\tilde{g}^{a,r}\right)_{i,j,k} - \left(\tilde{k}_{g1}+\tilde{k}_{g2}\right)^{a,r-1}_{i,j,k}(\tilde{g})^{a,r}_{i,j,k} = -\,\tilde{g}_C\left(\tilde{k}_{g1}\right)^{a,r-1}_{i,j,k} \tag{1.3.12}$$

$$\nabla\cdot\left(\widetilde{D}_w{}^{a,r-1}\,\nabla\,\widetilde{w}^{a,r}\right)_{i,j,k}-\left(\tilde{k}_f+\tilde{k}_w\right)_{i,j,k}^{a,r-1}(\widetilde{w})_{i,j,k}^{a,r}=-\left[\tilde{k}_{n2}\tilde{n}\;+\tilde{k}_r\tilde{b}\tilde{a}+\tilde{k}_w\widetilde{w}_C\right]_{i,j,k}^{a,r-1}\qquad(1.3.13)$$

$$\nabla\cdot\left(\widetilde{D}_\ell{}^{a,r-1}\nabla\tilde{\ell}^{a,r}\right)_{i,j,k}-\left(\tilde{k}_\ell\right)_{i,j,k}^{a,r-1}(\tilde{\ell})_{i,j,k}^{a,r}$$
$$-\tilde{z}_\ell\nabla\cdot\left[\widetilde{D}_\ell{}^{a,r-1}\,\tilde{\ell}^{a,r}\left(\frac{\tilde{z}_\ell\widetilde{D}_\ell\nabla\tilde{\ell}+\tilde{z}_b\widetilde{D}_b\nabla\tilde{b}+\widetilde{D}_a\nabla\tilde{a}+\tilde{z}_s\widetilde{D}_s\nabla\tilde{s}+\tilde{z}_r\widetilde{D}_r\nabla\tilde{r}}{\tilde{z}_\ell^2\,\widetilde{D}_\ell\,\tilde{\ell}+\tilde{z}_b^2\,\widetilde{D}_b\,\tilde{b}+\widetilde{D}_a\,\tilde{a}+\tilde{z}_s^2\,\widetilde{D}_s\,\tilde{s}+\tilde{z}_r^2\,\widetilde{D}_r\,\tilde{r}}\right)^{a,r-1}\right]_{i,j,k}\qquad(1.3.14)$$
$$=\frac{1}{3}\left(\tilde{k}_{n2}\tilde{n}\right)_{i,j,k}^{a,r-1}-\;2R_{g,n}\left(\tilde{k}_{g2}\tilde{g}\right)_{i,j,k}^{a,r-1}-\;\tilde{\ell}_C\left(\tilde{k}_\ell\right)_{i,j,k}^{a,r-1}$$

$$\nabla\cdot\left(\widetilde{D}_b{}^{a,r-1}\nabla\tilde{b}^{a,r}\right)_{i,j,k}-\;\tilde{k}_r(\tilde{a})_{i,j,k}^{a,r-1}(\tilde{b})_{i,j,k}^{a,r}$$
$$-\tilde{z}_b\nabla\cdot\left[\widetilde{D}_b{}^{a,r-1}\,\tilde{b}^{a,r}\left(\frac{\tilde{z}_\ell\widetilde{D}_\ell\nabla\tilde{\ell}+\tilde{z}_b\widetilde{D}_b\nabla\tilde{b}+\widetilde{D}_a\nabla\tilde{a}+\tilde{z}_s\widetilde{D}_s\nabla\tilde{s}+\tilde{z}_r\widetilde{D}_r\nabla\tilde{r}}{\tilde{z}_\ell^2\,\widetilde{D}_\ell\,\tilde{\ell}+\tilde{z}_b^2\,\widetilde{D}_b\,\tilde{b}+\widetilde{D}_a\,\tilde{a}+\tilde{z}_s^2\,\widetilde{D}_s\,\tilde{s}+\tilde{z}_r^2\,\widetilde{D}_r\,\tilde{r}}\right)^{a,r-1}\right]_{i,j,k}\qquad(1.3.15)$$
$$=\;-\tilde{k}_f(\widetilde{w})_{i,j,k}^{a,r-1}$$

$$\nabla\cdot\left(\widetilde{D}_a{}^{a,r-1}\nabla\tilde{a}^{a,r}\right)_{i,j,k}-\;\tilde{k}_r(\tilde{b})_{i,j,k}^{a,r-1}(\tilde{a})_{i,j,k}^{a,r}$$
$$-\tilde{z}_a\nabla\cdot\left[\widetilde{D}_a{}^{a,r-1}\,\tilde{a}^{a,r}\left(\frac{\tilde{z}_\ell\widetilde{D}_\ell\nabla\tilde{\ell}+\tilde{z}_b\widetilde{D}_b\nabla\tilde{b}+\widetilde{D}_a\nabla\tilde{a}+\tilde{z}_s\widetilde{D}_s\nabla\tilde{s}+\tilde{z}_r\widetilde{D}_r\nabla\tilde{r}}{\tilde{z}_\ell^2\,\widetilde{D}_\ell\,\tilde{\ell}+\tilde{z}_b^2\,\widetilde{D}_b\,\tilde{b}+\widetilde{D}_a\,\tilde{a}+\tilde{z}_s^2\,\widetilde{D}_s\,\tilde{s}+\tilde{z}_r^2\,\widetilde{D}_r\,\tilde{r}}\right)^{a,r-1}\right]_{i,j,k}\qquad(1.3.16)$$
$$=\;-2R_{g,n}\left(\tilde{k}_{g2}\tilde{g}\right)_{i,j,k}^{a,r-1}+\frac{1}{3}\left(\tilde{k}_{n2}\tilde{n}\right)_{i,j,k}^{a,r-1}-\;\tilde{k}_f(\widetilde{w})_{i,j,k}^{a,r-1}-\;\tilde{\ell}_C\left(\tilde{k}_\ell\right)_{i,j,k}^{a,r-1}+\;\left(\tilde{k}_\ell\tilde{\ell}\right)_{i,j,k}^{a,r-1}$$

$$\nabla\cdot\left(\widetilde{D}_s{}^{a,r-1}\,\nabla\,\tilde{s}^{a,r}\right)_{i,j,k}$$
$$-\tilde{z}_s\nabla\cdot\left[\widetilde{D}_s{}^{a,r-1}\,\tilde{s}^{a,r}\left(\frac{\tilde{z}_\ell\widetilde{D}_\ell\nabla\tilde{\ell}+\tilde{z}_b\widetilde{D}_b\nabla\tilde{b}+\widetilde{D}_a\nabla\tilde{a}+\tilde{z}_s\widetilde{D}_s\nabla\tilde{s}+\tilde{z}_r\widetilde{D}_r\nabla\tilde{r}}{\tilde{z}_\ell^2\,\widetilde{D}_\ell\,\tilde{\ell}+\tilde{z}_b^2\,\widetilde{D}_b\,\tilde{b}+\widetilde{D}_a\,\tilde{a}+\tilde{z}_s^2\,\widetilde{D}_s\,\tilde{s}+\tilde{z}_r^2\,\widetilde{D}_r\,\tilde{r}}\right)^{a,r-1}\right]_{i,j,k}=0\qquad(1.3.17)$$

$$(\tilde{r})_{i,j,k}^{a,r}=-\frac{1}{\tilde{z}_r}\left[\tilde{z}_\ell(\tilde{\ell})_{i,j,k}^{a,r}+\tilde{z}_b(\tilde{b})_{i,j,k}^{a,r}+\tilde{z}_a(\tilde{a})_{i,j,k}^{a,r}+\tilde{z}_s(\tilde{s})_{i,j,k}^{a,r}\right]\qquad(1.3.18)$$

Tumorigenic Species

$$\nabla\cdot\left(\widetilde{D}_{tgf}{}^{a,r-1}\,\nabla\,\widetilde{tgf}^{a,r}\right)_{i,j,k}-\left(\tilde{\lambda}_{tgf}+\tilde{\lambda}_{de,tgf}+\tilde{\lambda}_{U,tgf}\right)_{i,j,k}^{a,r-1}\left(\widetilde{tgf}\right)_{i,j,k}^{a,r}$$
$$=-\left(\tilde{\lambda}_{tgf}\right)_{i,j,k}^{a,r-1}\qquad(1.3.19)$$

$$\nabla\cdot\left(\widetilde{D}_{taf}{}^{a,r-1}\,\nabla\,\widetilde{taf}^{a,r}\right)_{i,j,k}-\left(\tilde{\lambda}_{taf}+\tilde{\lambda}_{de,taf}+\tilde{\lambda}_{U,taf}\right)_{i,j,k}^{a,r-1}\left(\widetilde{taf}\right)_{i,j,k}^{a,r}$$
$$=-\left(\tilde{\lambda}_{taf}\right)_{i,j,k}^{a,r-1}\qquad(1.3.20)$$

$$(\widetilde{m})_{i,j,k}^{a,r} - \frac{\theta}{2}\left[\nabla\cdot\left(\widetilde{D}_m{}^{a,r-1}\nabla\widetilde{m}^{a,r}\right)_{i,j,k}\right]$$
$$= (\widetilde{m})_{i,j,k}^{a-1} + \frac{\theta}{2}\left[\nabla\cdot\left(\widetilde{D}_m\nabla\widetilde{m}\right)_{i,j,k}^{a-1} + \left(\tilde{S}_m\right)_{i,j,k}^{a-1}\right] + \frac{\theta}{2}\left[\left(\tilde{S}_m\right)_{i,j,k}^{a,r-1}\right] \quad (1.3.21)$$

$$\left(\tilde{F}_E\right)_{i,j,k}^{a,r} = \left(\tilde{F}_E\right)_{i,j,k}^{a-1} - \frac{\theta}{2}\left[\nabla\cdot\left(\tilde{F}_E\tilde{\boldsymbol{u}}_E\right)_{i,j,k}^{a-1} + \nabla\cdot\left(\widetilde{D}_F\tilde{F}_E\nabla\widetilde{tgf}\right)_{i,j,k}^{a-1} - \left(\tilde{S}_{FE}\right)_{i,j,k}^{a-1}\right]$$
$$+ \frac{\theta}{2}\left[\left(\tilde{S}_{FE}\right)_{i,j,k}^{a,r-1} - \nabla\cdot\left(\tilde{F}_E\tilde{\boldsymbol{u}}_E\right)_{i,j,k}^{a,r-1} - \nabla\cdot\left(\widetilde{D}_F\tilde{F}_E\nabla\widetilde{tgf}\right)_{i,j,k}^{a,r-1}\right] \quad (1.3.22)$$

Blood and Lymphatic Vessels

$$\left(\tilde{B}_n^E\right)_{i,j,k}^{a,r} + \frac{\theta}{2}\Big\{\nabla\cdot\left[\tilde{\chi}_{che,BnE}\left(\mathcal{A}_{che,BnE}\right)^{a,r-1}\left(\tilde{B}_n^E\right)^{a,r}\nabla\,\widetilde{taf}^{a,r}\right]_{i,j,k}$$
$$+ \nabla\cdot\left[\tilde{\chi}_{hap,BnE}\left(\mathcal{A}_{hap,BnE}\right)^{a,r-1}\left(\tilde{B}_n^E\right)^{a,r}\nabla\left(\tilde{\phi}_E\right)^{a,r}\right]_{i,j,k}$$
$$- \nabla\cdot\left[\left(\widetilde{D}_{BnE}\right)^{a,r-1}\nabla\left(\tilde{B}_n^E\right)^{a,r}\right]_{i,j,k}\Big\}$$
$$= \left(\tilde{B}_n^E\right)_{i,j,k}^{a-1} - \frac{\theta}{2}\Big\{\nabla\cdot\left(\tilde{B}_n^E\tilde{\boldsymbol{u}}_E\right)_{i,j,k}^{a-1} + \nabla\cdot\left[\tilde{\chi}_{che,BnE}\left(\mathcal{A}_{che,BnE}\tilde{B}_n^E\nabla\widetilde{taf}\right)_{i,j,k}^{a-1}\right]$$
$$+ \nabla\cdot\left[\tilde{\chi}_{hap,BnE}\left(\mathcal{A}_{hap,BnE}\tilde{B}_n^E\nabla\tilde{\phi}_E\right)_{i,j,k}^{a-1}\right] - \nabla\cdot\left(\widetilde{D}_{BnE}\nabla\tilde{B}_n^E\right)_{i,j,k}^{a-1} - \left(\tilde{S}_{BnE}\right)_{i,j,k}^{a-1}\Big\}$$
$$- \frac{\theta}{2}\left[\nabla\cdot\left(\tilde{B}_n^E\,\tilde{\boldsymbol{u}}_E\right)_{i,j,k}^{a,r-1} - \left(\tilde{S}_{BnE}\right)_{i,j,k}^{a,r-1}\right] \quad (1.3.23)$$

$$\left(\tilde{L}_n^E\right)_{i,j,k}^{a,r} + \frac{\theta}{2}\Big\{\nabla\cdot\left[\tilde{\chi}_{che,LnE}\left(\mathcal{A}_{che,LnE}\right)^{a,r-1}\left(\tilde{L}_n^E\right)^{a,r}\nabla\,\widetilde{taf}^{a,r}\right]_{i,j,k}$$
$$+ \nabla\cdot\left[\tilde{\chi}_{hap,LnE}\left(\mathcal{A}_{hap,LnE}\right)^{a,r-1}\left(\tilde{L}_n^E\right)^{a,r}\nabla\left(\tilde{\phi}_E\right)^{a,r}\right]_{i,j,k}$$
$$- \nabla\cdot\left[\left(\widetilde{D}_{LnE}\right)^{a,r-1}\nabla\left(\tilde{L}_n^E\right)^{a,r}\right]_{i,j,k}\Big\}$$
$$= \left(\tilde{L}_n^E\right)_{i,j,k}^{a-1} - \frac{\theta}{2}\Big\{\nabla\cdot\left(\tilde{L}_n^E\,\tilde{\boldsymbol{u}}_E\right)_{i,j,k}^{a-1} + \nabla\cdot\left[\tilde{\chi}_{che,LnE}\left(\mathcal{A}_{che,LnE}\tilde{L}_n^E\nabla\widetilde{taf}\right)_{i,j,k}^{a-1}\right]$$
$$+ \nabla\cdot\left[\tilde{\chi}_{hap,LnE}\left(\mathcal{A}_{hap,LnE}\tilde{L}_n^E\nabla\tilde{\phi}_E\right)_{i,j,k}^{a-1}\right] - \nabla\cdot\left(\widetilde{D}_{LnE}\nabla\tilde{L}_n^E\right)_{i,j,k}^{a-1} - \left(\tilde{S}_{LnE}\right)_{i,j,k}^{a-1}\Big\}$$
$$- \frac{\theta}{2}\left[\nabla\cdot\left(\tilde{L}_n^E\,\tilde{\boldsymbol{u}}_E\right)_{i,j,k}^{a,r-1} - \left(\tilde{S}_{LnE}\right)_{i,j,k}^{a,r-1}\right] \quad (1.3.24)$$

Displacement vectors are computed at the beginning of each time step from $\nabla\cdot\widetilde{\mathbb{T}}_i = 0$, using relations listed in Eqs. (2.10) – (2.16) and known values from the previous iteration. The m–component of the displacement vector can therefore be written as (current unknown is shown in red):

$$4\left\{A_m\left(\tilde{L}_2+\tilde{L}_1\right)_{m+\frac{1}{2}}+A_m\left(\tilde{L}_2+\tilde{L}_1\right)_{m-\frac{1}{2}}+\sum_{n=\{x,y,z\}}\left[A_n\left(\tilde{L}_2\right)_{n+\frac{1}{2}}+A_n\left(\tilde{L}_2\right)_{n-\frac{1}{2}}\right]\right\}(u_m^d)_{i,j,k}^{a,r,n}$$
$$= 8\,A_m\left(\tilde{L}_2\right)_{m+\frac{1}{2}}(u_m^d)_{m+1}+8\,A_m\left(\tilde{L}_2\right)_{m-\frac{1}{2}}(u_m^d)_{m-1}$$
$$+A_m\left(\tilde{L}_1\right)_{m+\frac{1}{2}}\left\{\sum_{\substack{n=\{x,y,z\}\\ n\neq m}}\left[(u_n^d)_{m+1,n+1}+(u_n^d)_{n+1}-(u_n^d)_{m+1,n-1}-(u_n^d)_{n-1}\right]+4(u_m^d)_{m+1}\right\}$$
$$-A_m\left(\tilde{L}_1\right)_{m-\frac{1}{2}}\left\{\sum_{\substack{n=\{x,y,z\}\\ n\neq m}}\left[(u_n^d)_{m-1,n+1}+(u_n^d)_{n+1}-(u_n^d)_{m-1,n-1}-(u_n^d)_{n-1}\right]-4(u_m^d)_{m-1}\right\} \quad (1.3.25)$$
$$+\sum_{\substack{n=\{x,y,z\}\\ n\neq m}}\left\{A_n\left(\tilde{L}_2\right)_{n+\frac{1}{2}}\left[(u_n^d)_{m+1,n+1}+(u_n^d)_{m+1}-(u_n^d)_{m-1}-(u_n^d)_{m-1,n+1}+4(u_m^d)_{n+1}\right]\right.$$
$$\left.-A_n\left(\tilde{L}_2\right)_{n-\frac{1}{2}}\left[(u_n^d)_{m+1,n-1}+(u_n^d)_{m+1}-(u_n^d)_{m-1}-(u_n^d)_{m-1,n-1}-4(u_m^d)_{n-1}\right]\right\}$$
$$-2\,\eta\left[\left(\tilde{\mathbb{T}}_{T,m1}^*\right)_{i+1,j,k}-\left(\tilde{\mathbb{T}}_{T,m1}^*\right)_{i-1,j,k}+\left(\tilde{\mathbb{T}}_{T,m2}^*\right)_{i,j+1,k}-\left(\tilde{\mathbb{T}}_{T,m2}^*\right)_{i,j-1,k}+\left(\tilde{\mathbb{T}}_{T,m3}^*\right)_{i,j,k+1}\right.$$
$$\left.-\left(\tilde{\mathbb{T}}_{T,m3}^*\right)_{i,j,k-1}\right]$$

where the subscript m represents both the direction x, y, or z and its corresponding index i, j, or k. Terms $\tilde{\mathbb{T}}_{T,mi}^*$ are computed from

$$\tilde{\mathbb{T}}_{T,ij}^* = \tilde{\mathbb{T}}_{T,ji}^* = 2\,\tilde{L}_2\,\tilde{\mathcal{E}}_{ij}^* \quad (1.3.26)$$

$$\tilde{\mathbb{T}}_{T,ii}^* = 2\,\tilde{L}_2\,\tilde{\mathcal{E}}_{ii}^*+\tilde{L}_1\sum_{k=1}^{3}\tilde{\mathcal{E}}_{kk}^* \quad (1.3.27)$$

Let the set of variables be $\boldsymbol{\psi}$. With the exceptions of Eqs. (1.3.4), (1.3.5), (1.3.8) – (1.3.10) and (1.3.18), all terms on the RHS of equations above, which pertain to the previous time step $a-1$ and the previous iteration of the current time step $a, r-1$, for each variable $\varkappa$ are grouped as $R_\varkappa\left(\boldsymbol{\psi}_{i,j,k}^{a-1}, \boldsymbol{\psi}_{i,j,k}^{a,r-1}\right)$, and let the LHS of equations be $L_\varkappa\left(\boldsymbol{\psi}_{i,j,k}^{a,r}, \boldsymbol{\psi}_{i,j,k}^{a,r-1}\right)$. We aim to seek a unique solution for the set of equations $\boldsymbol{L}\left(\boldsymbol{\psi}_{i,j,k}^{a,r}, \boldsymbol{\psi}_{i,j,k}^{a,r-1}\right)=\boldsymbol{R}\left(\boldsymbol{\psi}_{i,j,k}^{a-1}, \boldsymbol{\psi}_{i,j,k}^{a,r-1}\right)$, where we have defined $\boldsymbol{L}=(L_V, L_D, \cdots, L_{LnE})$ as the operator terms and $\boldsymbol{R}=(R_V, R_D, \cdots, R_{LnE})$ as their corresponding source terms.

1.4 Nonlinear Gauss–Seidel Relaxations

The FAS uses local linearization such as the nonlinear Gauss-Seidel (GS) smoothing described in this section. We show the lexicographical Gauss-Seidel (GS-LEX) method here because of its simplicity.

In our relaxation procedure, the red-black ordering of the GS scheme (GS-RB) is used for faster convergence.

Letting the current smoothing pass be n, one smoothing step consists of relaxing the following system of equations lexicographically to obtain $\boldsymbol{\psi}_{i,j,k}^{a,r,n}$ (current time step, current iteration, and previous sweep are in peach; current time step, current iteration, and current sweep with already updated variables are in pink, while those variables to be updated are in red):

Cells and ECM Components

$$\begin{aligned}
&(\tilde{\phi}_V)_{i,j,k}^{a,r,n} + (\tilde{\mu}_T)_{i,j,k}^{a,r,n} \frac{\theta \widetilde{M}}{4\,\eta^2}\Big[(\tilde{\phi}_V)_{i-1,j,k}^{a,r,n} + (\tilde{\phi}_V)_{i,j-1,k}^{a,r,n} + (\tilde{\phi}_V)_{i,j,k-1}^{a,r,n} + 6\,(\tilde{\phi}_V)_{i,j,k}^{a,r,n-1} \\
&+(\tilde{\phi}_V)_{i+1,j,k}^{a,r,n-1} + (\tilde{\phi}_V)_{i,j+1,k}^{a,r,n-1} + (\tilde{\phi}_V)_{i,j,k+1}^{a,r,n-1}\Big] \\
&= R_V\big(\boldsymbol{\psi}_{i,j,k}^{a-1}, \boldsymbol{\psi}_{i,j,k}^{a,r-1}\big) + \frac{\theta \widetilde{M}}{4\,\eta^2}\Big\{(\tilde{\mu}_T)_{i+1,j,k}^{a,r,n-1}\Big[(\tilde{\phi}_V)_{i,j,k}^{a,r,n-1} + (\tilde{\phi}_V)_{i+1,j,k}^{a,r,n-1}\Big] \\
&\quad + (\tilde{\mu}_T)_{i,j,k+1}^{a,r,n-1}\Big[(\tilde{\phi}_V)_{i,j,k}^{a,r,n-1} + (\tilde{\phi}_V)_{i,j,k+1}^{a,r,n-1}\Big] + (\tilde{\mu}_T)_{i,j+1,k}^{a,r,n-1}\Big[(\tilde{\phi}_V)_{i,j,k}^{a,r,n-1} + (\tilde{\phi}_V)_{i,j+1,k}^{a,r,n-1}\Big] \\
&\quad + (\tilde{\mu}_T)_{i,j-1,k}^{a,r,n}\Big[(\tilde{\phi}_V)_{i,j,k}^{a,r,n-1} + (\tilde{\phi}_V)_{i,j-1,k}^{a,r,n}\Big] + (\tilde{\mu}_T)_{i-1,j,k}^{a,r,n}\Big[(\tilde{\phi}_V)_{i,j,k}^{a,r,n-1} + (\tilde{\phi}_V)_{i-1,j,k}^{a,r,n}\Big] \\
&\quad + (\tilde{\mu}_T)_{i,j,k-1}^{a,r,n}\Big[(\tilde{\phi}_V)_{i,j,k}^{a,r,n-1} + (\tilde{\phi}_V)_{i,j,k-1}^{a,r,n}\Big]\Big\}
\end{aligned} \tag{1.4.1}$$

$$\begin{aligned}
&(\tilde{\phi}_D)_{i,j,k}^{a,r,n} + (\tilde{\mu}_T)_{i,j,k}^{a,r,n} \frac{\theta \widetilde{M}}{4\,\eta^2}\Big[(\tilde{\phi}_D)_{i-1,j,k}^{a,r,n} + (\tilde{\phi}_D)_{i,j-1,k}^{a,r,n} + (\tilde{\phi}_D)_{i,j,k-1}^{a,r,n} + 6\,(\tilde{\phi}_D)_{i,j,k}^{a,r,n-1} \\
&+(\tilde{\phi}_D)_{i+1,j,k}^{a,r,n-1} + (\tilde{\phi}_D)_{i,j+1,k}^{a,r,n-1} + (\tilde{\phi}_D)_{i,j,k+1}^{a,r,n-1}\Big] \\
&= R_D\big(\boldsymbol{\psi}_{i,j,k}^{a-1}, \boldsymbol{\psi}_{i,j,k}^{a,r-1}\big) + \frac{\theta \widetilde{M}}{4\,\eta^2}\Big\{(\tilde{\mu}_T)_{i+1,j,k}^{a,r,n-1}\Big[(\tilde{\phi}_D)_{i,j,k}^{a,r,n-1} + (\tilde{\phi}_D)_{i+1,j,k}^{a,r,n-1}\Big] \\
&\quad + (\tilde{\mu}_T)_{i,j,k+1}^{a,r,n-1}\Big[(\tilde{\phi}_D)_{i,j,k}^{a,r,n-1} + (\tilde{\phi}_D)_{i,j,k+1}^{a,r,n-1}\Big] + (\tilde{\mu}_T)_{i,j+1,k}^{a,r,n-1}\Big[(\tilde{\phi}_D)_{i,j,k}^{a,r,n-1} + (\tilde{\phi}_D)_{i,j+1,k}^{a,r,n-1}\Big] \\
&\quad + (\tilde{\mu}_T)_{i,j-1,k}^{a,r,n}\Big[(\tilde{\phi}_D)_{i,j,k}^{a,r,n-1} + (\tilde{\phi}_D)_{i,j-1,k}^{a,r,n}\Big] + (\tilde{\mu}_T)_{i-1,j,k}^{a,r,n}\Big[(\tilde{\phi}_D)_{i,j,k}^{a,r,n-1} + (\tilde{\phi}_D)_{i-1,j,k}^{a,r,n}\Big] \\
&\quad + (\tilde{\mu}_T)_{i,j,k-1}^{a,r,n}\Big[(\tilde{\phi}_D)_{i,j,k}^{a,r,n-1} + (\tilde{\phi}_D)_{i,j,k-1}^{a,r,n}\Big]\Big\}
\end{aligned} \tag{1.4.2}$$

$$\begin{aligned}
&(\tilde{\phi}_E)_{i,j,k}^{a,r,n} + (\tilde{\mu}_E)_{i,j,k}^{a,r,n} \frac{\theta \widetilde{M}}{4\,\eta^2}\Big[(\tilde{\phi}_E)_{i-1,j,k}^{a,r,n} + (\tilde{\phi}_E)_{i,j-1,k}^{a,r,n} + (\tilde{\phi}_E)_{i,j,k-1}^{a,r,n} + 6\,(\tilde{\phi}_E)_{i,j,k}^{a,r,n-1} \\
&+(\tilde{\phi}_E)_{i+1,j,k}^{a,r,n-1} + (\tilde{\phi}_E)_{i,j+1,k}^{a,r,n-1} + (\tilde{\phi}_E)_{i,j,k+1}^{a,r,n-1}\Big] \\
&= R_E\big(\boldsymbol{\psi}_{i,j,k}^{a-1}, \boldsymbol{\psi}_{i,j,k}^{a,r-1}\big) + \frac{\theta \widetilde{M}}{4\,\eta^2}\Big\{(\tilde{\mu}_E)_{i+1,j,k}^{a,r,n-1}\Big[(\tilde{\phi}_E)_{i,j,k}^{a,r,n-1} + (\tilde{\phi}_E)_{i+1,j,k}^{a,r,n-1}\Big] \\
&\quad + (\tilde{\mu}_E)_{i,j,k+1}^{a,r,n-1}\Big[(\tilde{\phi}_E)_{i,j,k}^{a,r,n-1} + (\tilde{\phi}_E)_{i,j,k+1}^{a,r,n-1}\Big] + (\tilde{\mu}_E)_{i,j+1,k}^{a,r,n-1}\Big[(\tilde{\phi}_E)_{i,j,k}^{a,r,n-1} + (\tilde{\phi}_E)_{i,j+1,k}^{a,r,n-1}\Big] \\
&\quad + (\tilde{\mu}_E)_{i,j-1,k}^{a,r,n}\Big[(\tilde{\phi}_E)_{i,j,k}^{a,r,n-1} + (\tilde{\phi}_E)_{i,j-1,k}^{a,r,n}\Big] + (\tilde{\mu}_E)_{i-1,j,k}^{a,r,n}\Big[(\tilde{\phi}_E)_{i,j,k}^{a,r,n-1} + (\tilde{\phi}_E)_{i-1,j,k}^{a,r,n}\Big] \\
&\quad + (\tilde{\mu}_E)_{i,j,k-1}^{a,r,n}\Big[(\tilde{\phi}_E)_{i,j,k}^{a,r,n-1} + (\tilde{\phi}_E)_{i,j,k-1}^{a,r,n}\Big]\Big\}
\end{aligned} \tag{1.4.3}$$

Chemical Potentials

$$
\begin{aligned}
&(\tilde{\mu}_T)^{a,r,n}_{i,j,k} - \left[\frac{6\tilde{\epsilon}_T{}^2}{\eta^2} + \left(\frac{\partial^2 \tilde{F}_b}{\partial \tilde{\phi}_T^2}\right)^{a,r,n-1}_{i,j,k}\right](\tilde{\phi}_T)^{a,r,n}_{i,j,k} - \frac{6\tilde{\epsilon}_{TE}{}^2}{\eta^2}(\tilde{\phi}_E)^{a,r,n}_{i,j,k} \\
&= R_{\mu T}\left(\boldsymbol{\Psi}^{a-1}_{i,j,k}, \boldsymbol{\Psi}^{a,r-1}_{i,j,k}\right) + \left[\left(\frac{\partial \tilde{F}_b}{\partial \tilde{\phi}_T}\right)^{a,r,n-1}_{i,j,k} - (\tilde{\phi}_T)^{a,r,n-1}_{i,j,k}\left(\frac{\partial^2 \tilde{F}_b}{\partial \tilde{\phi}_T^2}\right)^{a,r,n-1}_{i,j,k}\right] \\
&- \frac{\tilde{\epsilon}_T{}^2}{\eta^2}\left[(\tilde{\phi}_T)^{a,r,n}_{i-1,j,k} + (\tilde{\phi}_T)^{a,r,n}_{i,j-1,k} + (\tilde{\phi}_T)^{a,r,n}_{i,j,k-1} + (\tilde{\phi}_T)^{a,r,n-1}_{i+1,j,k} + (\tilde{\phi}_T)^{a,r,n-1}_{i,j+1,k} + (\tilde{\phi}_T)^{a,r,n-1}_{i,j,k+1}\right] \\
&- \frac{\tilde{\epsilon}_{TE}{}^2}{\eta^2}\left[(\tilde{\phi}_E)^{a,r,n}_{i-1,j,k} + (\tilde{\phi}_E)^{a,r,n}_{i,j-1,k} + (\tilde{\phi}_E)^{a,r,n}_{i,j,k-1} + (\tilde{\phi}_E)^{a,r,n-1}_{i+1,j,k} + (\tilde{\phi}_E)^{a,r,n-1}_{i,j+1,k} + (\tilde{\phi}_E)^{a,r,n-1}_{i,j,k+1}\right]
\end{aligned}
\tag{1.4.4}
$$

$$
\begin{aligned}
&(\tilde{\mu}_E)^{a,r,n}_{i,j,k} - \left[\frac{6\,\tilde{\epsilon}_E{}^2}{\eta^2} + \left(\frac{\partial^2 \tilde{F}_b}{\partial \tilde{\phi}_E^2}\right)^{a,r,n-1}_{i,j,k} + \left(\frac{\partial^2 \widetilde{\mathcal{W}}}{\partial \tilde{\phi}_E^2}\right)^{a,r,n-1}_{i,j,k}\right](\tilde{\phi}_E)^{a,r,n}_{i,j,k} \\
&- \frac{6\,\tilde{\epsilon}_{TE}{}^2}{\eta^2}(\tilde{\phi}_T)^{a,r,n}_{i,j,k} \\
&= R_{\mu E}\left(\boldsymbol{\Psi}^{a-1}_{i,j,k}, \boldsymbol{\Psi}^{a,r-1}_{i,j,k}\right) + \left[\left(\frac{\partial \tilde{F}_b}{\partial \tilde{\phi}_E}\right)^{a,r,n-1}_{i,j,k} - (\tilde{\phi}_E)^{a,r,n-1}_{i,j,k}\left(\frac{\partial^2 \tilde{F}_b}{\partial \tilde{\phi}_E^2}\right)^{a,r,n-1}_{i,j,k}\right] \\
&+ \left[\left(\frac{\partial \widetilde{\mathcal{W}}}{\partial \tilde{\phi}_E}\right)^{a,r,n-1}_{i,j,k} - (\tilde{\phi}_E)^{a,r,n-1}_{i,j,k}\left(\frac{\partial^2 \widetilde{\mathcal{W}}}{\partial \tilde{\phi}_E^2}\right)^{a,r,n-1}_{i,j,k}\right] \\
&- \frac{\tilde{\epsilon}_E{}^2}{\eta^2}\left[(\tilde{\phi}_E)^{a,r,n}_{i-1,j,k} + (\tilde{\phi}_E)^{a,r,n}_{i,j-1,k} + (\tilde{\phi}_E)^{a,r,n}_{i,j,k-1} + (\tilde{\phi}_E)^{a,r,n-1}_{i+1,j,k} + (\tilde{\phi}_E)^{a,r,n-1}_{i,j+1,k} + (\tilde{\phi}_E)^{a,r,n-1}_{i,j,k+1}\right] \\
&- \frac{\tilde{\epsilon}_{TE}{}^2}{\eta^2}\left[(\tilde{\phi}_T)^{a,r,n}_{i-1,j,k} + (\tilde{\phi}_T)^{a,r,n}_{i,j-1,k} + (\tilde{\phi}_T)^{a,r,n}_{i,j,k-1} + (\tilde{\phi}_T)^{a,r,n-1}_{i+1,j,k} + (\tilde{\phi}_T)^{a,r,n-1}_{i,j+1,k} + (\tilde{\phi}_T)^{a,r,n-1}_{i,j,k+1}\right]
\end{aligned}
\tag{1.4.5}
$$

Pressures and Velocities

$$
-\frac{1}{2\eta^2}(\tilde{\varphi}^k_\alpha)^{a,r-1}(\tilde{p})^{a,r,n}_{i,j,k} = R_p\left(\boldsymbol{\Psi}^{a-1}_{i,j,k}, \boldsymbol{\Psi}^{a,r-1}_{i,j,k}\right) - \frac{1}{2\eta^2}\left[\left(\tilde{\varphi}_1^{k\alpha,p}\right)^{a,r,n-1} + \left(\tilde{\varphi}_2^{k\alpha,p}\right)^{a,r,n}\right] \tag{1.4.6}
$$

$$
\begin{aligned}
-\frac{6}{\eta^2}(\tilde{q})^{a,r,n}_{i,j,k} = R_q\left(\boldsymbol{\Psi}^{a-1}_{i,j,k}, \boldsymbol{\Psi}^{a,r-1}_{i,j,k}\right) - \frac{1}{\eta^2}\Big[&(\tilde{q})^{a,r,n}_{i-1,j,k} + (\tilde{q})^{a,r,n}_{i,j-1,k} + (\tilde{q})^{a,r,n}_{i,j,k-1} \\
&+ (\tilde{q})^{a,r,n-1}_{i+1,j,k} + (\tilde{q})^{a,r,n-1}_{i,j+1,k} + (\tilde{q})^{a,r,n-1}_{i,j,k+1}\Big]
\end{aligned}
\tag{1.4.7}
$$

$$
\begin{aligned}
(\tilde{\boldsymbol{u}}_\alpha)^{a,r,n}_{i,j,k} = -\tilde{k}_\alpha\Big[&\nabla(\tilde{p})^{a,r,n}_{i,j,k} - \frac{\tilde{\gamma}_T}{\tilde{\epsilon}_T}(\tilde{\mu}_T)^{a,r,n}_{i,j,k}\nabla(\tilde{\phi}_T)^{a,r,n}_{i,j,k} - \frac{\tilde{\gamma}_T}{\tilde{\epsilon}_T}(\tilde{\mu}_M)^{a,r,n}_{i,j,k}\nabla(\tilde{\phi}_M)^{a,r,n}_{i,j,k} \\
&- \frac{\tilde{\gamma}_G}{\tilde{\epsilon}_G}(\tilde{\mu}_G)^{a,r,n}_{i,j,k}\nabla(\tilde{\phi}_G)^{a,r,n}_{i,j,k} - \frac{\tilde{\gamma}_E}{\tilde{\epsilon}_E}(\tilde{\mu}_E)^{a,r,n}_{i,j,k}\nabla(\tilde{\phi}_E)^{a,r,n}_{i,j,k}\Big]
\end{aligned}
\tag{1.4.8}
$$

$$
(\tilde{\boldsymbol{u}}_\beta)^{a,r,n}_{i,j,k} = -\,\tilde{k}_\beta\nabla(\tilde{q})^{a,r,n}_{i,j,k} \tag{1.4.9}
$$

$$
(\tilde{\boldsymbol{u}}_E)^{a,r,n}_{i,j,k} = (\tilde{\boldsymbol{u}}_\alpha)^{a,r,n}_{i,j,k} - \widetilde{M}\nabla(\tilde{\mu}_E)^{a,r,n}_{i,j,k} \tag{1.4.10}
$$

Nutrients and Waste Products

$$-\left[\left(\tilde{k}_{n1}+\tilde{k}_{n2}\right)_{i,j,k}^{a,r-1}+\frac{1}{2\,\eta^2}(\tilde{\varphi}_n^D)^{a,r-1}\right](\tilde{n})_{i,j,k}^{a,r,n}$$
$$=R_n\left(\boldsymbol{\Psi}_{i,j,k}^{a-1},\boldsymbol{\Psi}_{i,j,k}^{a,r-1}\right)-\frac{1}{2\,\eta^2}\left[\left(\tilde{\varphi}_1^{D,n}\right)^{a,r,n-1}+\left(\tilde{\varphi}_2^{D,n}\right)^{a,r,n}\right] \quad (1.4.11)$$

$$-\left[\left(\tilde{k}_{g1}+\tilde{k}_{g2}\right)_{i,j,k}^{a,r-1}+\frac{1}{2\,\eta^2}(\tilde{\varphi}_g^D)^{a,r-1}\right](\tilde{g})_{i,j,k}^{a,r,n}$$
$$=R_g\left(\boldsymbol{\Psi}_{i,j,k}^{a-1},\boldsymbol{\Psi}_{i,j,k}^{a,r-1}\right)-\frac{1}{2\,\eta^2}\left[\left(\tilde{\varphi}_1^{D,g}\right)^{a,r,n-1}+\left(\tilde{\varphi}_2^{D,g}\right)^{a,r,n}\right] \quad (1.4.12)$$

$$-\left[\left(\tilde{k}_f+\tilde{k}_w\right)_{i,j,k}^{a,r-1}+\frac{1}{2\,\eta^2}(\tilde{\varphi}_w^D)^{a,r-1}\right](\tilde{w})_{i,j,k}^{a,r,n}$$
$$=R_w\left(\boldsymbol{\Psi}_{i,j,k}^{a-1},\boldsymbol{\Psi}_{i,j,k}^{a,r-1}\right)-\frac{1}{2\,\eta^2}\left[\left(\tilde{\varphi}_1^{D,w}\right)^{a,r,n-1}+\left(\tilde{\varphi}_2^{D,w}\right)^{a,r,n}\right] \quad (1.4.13)$$

$$-\left[\frac{1}{2\,\eta^2}(\tilde{\varphi}_\ell^D)^{a,r-1}+\frac{\tilde{z}_\ell}{2\eta}\left(\tilde{\varphi}_{\mathcal{N}\ell,\ell}+\tilde{\varphi}_{\mathcal{N}\ell,b}+\tilde{\varphi}_{\mathcal{N}\ell,a}+\tilde{\varphi}_{\mathcal{N}\ell,s}+\tilde{\varphi}_{\mathcal{N}\ell,r}\right)\right.$$
$$\left.+\left(\tilde{k}_\ell\right)_{i,j,k}^{a,r-1}\right](\tilde{\ell})_{i,j,k}^{a,r,n}$$
$$=R_\ell\left(\boldsymbol{\Psi}_{i,j,k}^{a-1},\boldsymbol{\Psi}_{i,j,k}^{a,r-1}\right)-\frac{1}{2\,\eta^2}\left[\left(\tilde{\varphi}_1^{D,\ell}\right)^{a,r,n-1}+\left(\tilde{\varphi}_2^{D,\ell}\right)^{a,r,n}\right] \quad (1.4.14)$$
$$+\frac{\tilde{z}_\ell}{2\eta}\left(\tilde{\varphi}_{\mathcal{N}\ell,\ell,\ell}+\tilde{\varphi}_{\mathcal{N}\ell,b,\ell}+\tilde{\varphi}_{\mathcal{N}\ell,a,\ell}+\tilde{\varphi}_{\mathcal{N}\ell,s,\ell}+\tilde{\varphi}_{\mathcal{N}\ell,r,\ell}\right)$$

$$-\left[\frac{1}{2\,\eta^2}(\tilde{\varphi}_b^D)^{a,r-1}+\frac{\tilde{z}_b}{2\eta}\left(\tilde{\varphi}_{\mathcal{N}b,\ell}+\tilde{\varphi}_{\mathcal{N}b,b}+\tilde{\varphi}_{\mathcal{N}b,a}+\tilde{\varphi}_{\mathcal{N}b,s}+\tilde{\varphi}_{\mathcal{N}b,r}\right)\right.$$
$$\left.+\tilde{k}_r(\tilde{a})_{i,j,k}^{a,r-1}\right](\tilde{b})_{i,j,k}^{a,r,n}$$
$$=R_b\left(\boldsymbol{\Psi}_{i,j,k}^{a-1},\boldsymbol{\Psi}_{i,j,k}^{a,r-1}\right)-\frac{1}{2\,\eta^2}\left[\left(\tilde{\varphi}_1^{D,b}\right)^{a,r,n-1}+\left(\tilde{\varphi}_2^{D,b}\right)^{a,r,n}\right] \quad (1.4.15)$$
$$+\frac{\tilde{z}_b}{2\eta}\left(\tilde{\varphi}_{\mathcal{N}b,\ell,b}+\tilde{\varphi}_{\mathcal{N}b,b,b}+\tilde{\varphi}_{\mathcal{N}b,a,b}+\tilde{\varphi}_{\mathcal{N}b,s,b}+\tilde{\varphi}_{\mathcal{N}b,r,b}\right)$$

$$-\left[\frac{1}{2\,\eta^2}(\tilde{\varphi}_a^D)^{a,r-1}+\frac{\tilde{z}_a}{2\eta}\left(\tilde{\varphi}_{\mathcal{N}a,\ell}+\tilde{\varphi}_{\mathcal{N}a,b}+\tilde{\varphi}_{\mathcal{N}a,a}+\tilde{\varphi}_{\mathcal{N}a,s}+\tilde{\varphi}_{\mathcal{N}a,r}\right)\right.$$
$$\left.+\tilde{k}_r\left(\tilde{b}\right)_{i,j,k}^{a,r-1}\right](\tilde{a})_{i,j,k}^{a,r,n}$$
$$=R_a\left(\boldsymbol{\Psi}_{i,j,k}^{a-1},\boldsymbol{\Psi}_{i,j,k}^{a,r-1}\right)-\frac{1}{2\,\eta^2}\left[\left(\tilde{\varphi}_1^{D,a}\right)^{a,r,n-1}+\left(\tilde{\varphi}_2^{D,a}\right)^{a,r,n}\right] \quad (1.4.16)$$
$$+\frac{\tilde{z}_a}{2\eta}\left(\tilde{\varphi}_{\mathcal{N}a,\ell,a}+\tilde{\varphi}_{\mathcal{N}a,b,a}+\tilde{\varphi}_{\mathcal{N}a,a,a}+\tilde{\varphi}_{\mathcal{N}a,s,a}+\tilde{\varphi}_{\mathcal{N}a,r,a}\right)$$

$$-\left[\frac{1}{2\,\eta^2}(\tilde{\varphi}_s^D)^{a,r-1} + \frac{\tilde{z}_s}{2\eta}\left(\tilde{\varphi}_{\mathcal{N}s,\ell} + \tilde{\varphi}_{\mathcal{N}s,b} + \tilde{\varphi}_{\mathcal{N}s,a} + \tilde{\varphi}_{\mathcal{N}s,s} + \tilde{\varphi}_{\mathcal{N}s,r}\right)\right](\tilde{s})_{i,j,k}^{a,r,n}$$
$$= R_s\left(\boldsymbol{\psi}_{i,j,k}^{a-1}, \boldsymbol{\psi}_{i,j,k}^{a,r-1}\right) - \frac{1}{2\,\eta^2}\left[\left(\tilde{\varphi}_1^{D,s}\right)^{a,r,n-1} + \left(\tilde{\varphi}_2^{D,s}\right)^{a,r,n}\right] \tag{1.4.17}$$
$$+ \frac{\tilde{z}_s}{2\eta}\left(\tilde{\varphi}_{\mathcal{N}s,\ell,s} + \tilde{\varphi}_{\mathcal{N}s,b,s} + \tilde{\varphi}_{\mathcal{N}s,a,s} + \tilde{\varphi}_{\mathcal{N}s,s,s} + \tilde{\varphi}_{\mathcal{N}s,r,s}\right)$$

$$(\tilde{r})_{i,j,k}^{a,r,n} = -\frac{1}{\tilde{z}_r}\left[\tilde{z}_\ell(\tilde{\ell})_{i,j,k}^{a,r,n} + \tilde{z}_b(\tilde{b})_{i,j,k}^{a,r,n} + \tilde{z}_a(\tilde{a})_{i,j,k}^{a,r,n} + \tilde{z}_s(\tilde{s})_{i,j,k}^{a,r,n}\right] \tag{1.4.18}$$

Tumorigenic Species

$$-\left[\left(\tilde{\lambda}_{tgf} + \tilde{\lambda}_{de,tgf} + \tilde{\lambda}_{U,tgf}\right)_{i,j,k}^{a,r-1} + \frac{1}{2\,\eta^2}\left(\tilde{\varphi}_{tgf}^D\right)^{a,r-1}\right](\widetilde{tgf})_{i,j,k}^{a,r,n}$$
$$= R_{tgf}\left(\boldsymbol{\psi}_{i,j,k}^{a-1}, \boldsymbol{\psi}_{i,j,k}^{a,r-1}\right) - \frac{1}{2\,\eta^2}\left[\left(\tilde{\varphi}_1^{D,tgf}\right)^{a,r,n-1} + \left(\tilde{\varphi}_2^{D,tgf}\right)^{a,r,n}\right] \tag{1.4.19}$$

$$-\left[\left(\tilde{\lambda}_{taf} + \tilde{\lambda}_{de,taf} + \tilde{\lambda}_{U,taf}\right)_{i,j,k}^{a,r-1} + \frac{1}{2\,\eta^2}\left(\tilde{\varphi}_{taf}^D\right)^{a,r-1}\right](\widetilde{taf})_{i,j,k}^{a,r,n}$$
$$= R_{taf}\left(\boldsymbol{\psi}_{i,j,k}^{a-1}, \boldsymbol{\psi}_{i,j,k}^{a,r-1}\right) - \frac{1}{2\,\eta^2}\left[\left(\tilde{\varphi}_1^{D,taf}\right)^{a,r,n-1} + \left(\tilde{\varphi}_2^{D,taf}\right)^{a,r,n}\right] \tag{1.4.20}$$

$$\left[1 + \frac{\theta}{4\,\eta^2}(\tilde{\varphi}_m^D)^{a,r-1}\right](\tilde{m})_{i,j,k}^{a,r,n}$$
$$= R_m\left(\boldsymbol{\psi}_{i,j,k}^{a-1}, \boldsymbol{\psi}_{i,j,k}^{a,r-1}\right) + \frac{\theta}{4\,\eta^2}\left[\left(\tilde{\varphi}_1^{D,m}\right)^{a,r,n-1} + \left(\tilde{\varphi}_2^{D,m}\right)^{a,r,n}\right] \tag{1.4.21}$$

$$\left(\tilde{F}_E\right)_{i,j,k}^{a,r,n} = R_{FE}\left(\boldsymbol{\psi}_{i,j,k}^{a-1}, \boldsymbol{\psi}_{i,j,k}^{a,r-1}\right) \tag{1.4.22}$$

Blood and Lymphatic Vessels

$$\left\{1 + \frac{\theta}{4\eta}\left[\tilde{\chi}_{che,BnE}\tilde{\varphi}_{CheB,taf} + \tilde{\chi}_{hap,BnE}\tilde{\varphi}_{HapB,E} + \frac{1}{\eta}(\tilde{\varphi}_{BnE}^D)^{a,r-1}\right]\right\}(\tilde{B}_n^E)_{i,j,k}^{a,r,n}$$
$$= R_{BnE}\left(\boldsymbol{\psi}_{i,j,k}^{a-1}, \boldsymbol{\psi}_{i,j,k}^{a,r-1}\right) - \frac{\theta}{4\eta}\left\{\tilde{\chi}_{che,BnE}\tilde{\varphi}_{CheB,BnE,taf} + \tilde{\chi}_{hap,BnE}\tilde{\varphi}_{HapB,BnE,E}\right. \tag{1.4.23}$$
$$\left.- \frac{1}{\eta}\left[\left(\tilde{\varphi}_1^{D,BnE}\right)^{a,r,n-1} + \left(\tilde{\varphi}_2^{D,BnE}\right)^{a,r,n}\right]\right\}$$

$$\left\{1 + \frac{\theta}{4\eta}\left[\tilde{\chi}_{che,LnE}\tilde{\varphi}_{CheL,taf} + \tilde{\chi}_{hap,LnE}\tilde{\varphi}_{HapL,E} + \frac{1}{\eta}(\tilde{\varphi}_{LnE}^D)^{a,r-1}\right]\right\}(\tilde{L}_n^E)_{i,j,k}^{a,r,n}$$
$$= R_{LnE}\left(\boldsymbol{\psi}_{i,j,k}^{a-1}, \boldsymbol{\psi}_{i,j,k}^{a,r-1}\right) - \frac{\theta}{4\eta}\left\{\tilde{\chi}_{che,LnE}\tilde{\varphi}_{CheL,LnE,taf} + \tilde{\chi}_{hap,LnE}\tilde{\varphi}_{HapL,LnE,E}\right. \tag{1.4.24}$$
$$\left.- \frac{1}{\eta}\left[\left(\tilde{\varphi}_1^{D,LnE}\right)^{a,r,n-1} + \left(\tilde{\varphi}_2^{D,LnE}\right)^{a,r,n}\right]\right\}$$

Tissue myofibroblastic cell concentration is obtained from $(\tilde{F})_{i,j,k}^{a,r,n} = (\tilde{\phi}_E)_{i,j,k}^{a,r,n}(\tilde{F}_E)_{i,j,k}^{a,r,n}$, whereas tissue blood and lymphatic vessel concentrations are calculated from their concentrations in ECM, $(\tilde{B}_n)_{i,j,k}^{a,r,n} = (\tilde{\phi}_E)_{i,j,k}^{a,r,n}(\tilde{B}_n^E)_{i,j,k}^{a,r,n}$ and $(\tilde{L}_n)_{i,j,k}^{a,r,n} = (\tilde{\phi}_E)_{i,j,k}^{a,r,n}(\tilde{L}_n^E)_{i,j,k}^{a,r,n}$.

The second-derivative terms in Eqs. (1.4.4) & (1.4.5) result from the Taylor expansion used in estimating $(\partial\tilde{F}_b/\partial\tilde{\phi}_T)_{i,j,k}^{a,r,n}$, $(\partial\tilde{F}_b/\partial\tilde{\phi}_E)_{i,j,k}^{a,r,n}$, and $(\partial\tilde{\mathcal{W}}/\partial\tilde{\phi}_E)_{i,j,k}^{a,r,n}$. Using Eqs. (2.7) – (2.9), the second derivative terms are computed as:

$$\left(\frac{\partial^2\tilde{F}_b}{\partial\tilde{\phi}_T^2}\right)_{i,j,k}^{a,r,n-1} = 12A_1(\tilde{\phi}_T)_{i,j,k}^{a,r,n-1}\left[(\tilde{\phi}_T)_{i,j,k}^{a,r,n-1} + (\tilde{\phi}_E)_{i,j,k}^{a,r,n-1} - 1\right] + 2A_1\left[(\tilde{\phi}_E)_{i,j,k}^{a,r,n-1} - 1\right]^2 \quad (1.4.25)$$

$$\left(\frac{\partial^2\tilde{F}_b}{\partial\tilde{\phi}_E^2}\right)_{i,j,k}^{a,r,n-1} = 2A_1\left[(\tilde{\phi}_T)_{i,j,k}^{a,r,n-1}\right]^2 - 6(\tilde{\phi}_E)_{i,j,k}^{a,r,n-1} + 2(1 + A_2 + A_4 - A_5) \quad (1.4.26)$$

$$\left(\frac{\partial^2\tilde{\mathcal{W}}}{\partial\tilde{\phi}_E^2}\right)_{i,j,k}^{a,r,n-1} = \tilde{\epsilon}_E\left\{6\left[1 - 2(\tilde{\phi}_E)_{i,j,k}^{a,r,n-1}\right]\sum_{m,n=1}^{3}\left[\frac{1}{2}(\tilde{\mathcal{E}}_T)_{mn}\tilde{\mathbb{T}}_{mn}^* - (\tilde{\mathcal{E}}_T^*)_{mn}\tilde{\mathbb{T}}_{mn}\right] + \left[6(\tilde{\phi}_E)_{i,j,k}^{a,r,n-1}\left(1 - (\tilde{\phi}_E)_{i,j,k}^{a,r,n-1}\right)\right]^2\sum_{m,n=1}^{3}(\tilde{\mathcal{E}}_T^*)_{mn}\left[(\tilde{\mathbb{T}}_T)_{mn} - 2\,\tilde{\mathbb{T}}_{mn}^*\right]\right\} \quad (1.4.27)$$

where $(\tilde{\mathcal{E}}_T)_{mn}$, $\tilde{\mathbb{T}}_{mn}^*$, $(\tilde{\mathcal{E}}_T^*)_{mn}$, and $\tilde{\mathbb{T}}_{mn}$ are calculated as in Eqs. (2.10) – (2.16) and $(\tilde{\mathbb{T}}_T)_{mn}$ is given by

$$(\tilde{\mathbb{T}}_T)_{mn} = 2\,\tilde{L}_2\,(\tilde{\mathcal{E}}_T^*)_{mn} + \tilde{L}_1\delta_{mn}\sum_{k=1}^{3}(\tilde{\mathcal{E}}_T^*)_{kk}\ . \quad (1.4.28)$$

In Eqs. (1.4.14) – (1.4.17), terms in the forms of $\tilde{\varphi}_{\mathcal{N}\sigma,\gamma}$ and $\tilde{\varphi}_{\mathcal{N}\sigma,\gamma,\sigma}$ are computed as:

$$
\begin{aligned}
\tilde{\varphi}_{\mathcal{N}\sigma,\gamma} = \;& A_x\left(\widetilde{\mathcal{N}}_\sigma^\gamma\right)_{i+\frac{1}{2},j,k}^{a,r-1} D_x(\tilde{\gamma})_{i+\frac{1}{2},j,k}^{a,r-1} - A_x\left(\widetilde{\mathcal{N}}_\sigma^\gamma\right)_{i-\frac{1}{2},j,k}^{a,r-1} D_x(\tilde{\gamma})_{i-\frac{1}{2},j,k}^{a,r-1} \\
&+ A_y\left(\widetilde{\mathcal{N}}_\sigma^\gamma\right)_{i,j+\frac{1}{2},k}^{a,r-1} D_y(\tilde{\gamma})_{i,j+\frac{1}{2},k}^{a,r-1} - A_y\left(\widetilde{\mathcal{N}}_\sigma^\gamma\right)_{i,j-\frac{1}{2},k}^{a,r-1} D_y(\tilde{\gamma})_{i,j-\frac{1}{2},k}^{a,r-1} \\
&+ A_z\left(\widetilde{\mathcal{N}}_\sigma^\gamma\right)_{i,j,k+\frac{1}{2}}^{a,r-1} D_z(\tilde{\gamma})_{i,j,k+\frac{1}{2}}^{a,r-1} - A_z\left(\widetilde{\mathcal{N}}_\sigma^\gamma\right)_{i,j,k-\frac{1}{2}}^{a,r-1} D_z(\tilde{\gamma})_{i,j,k-\frac{1}{2}}^{a,r-1} \\
= \;& \tilde{\varphi}_{\sigma\gamma 1} - \tilde{\varphi}_{\sigma\gamma 2} + \tilde{\varphi}_{\sigma\gamma 3} - \tilde{\varphi}_{\sigma\gamma 4} + \tilde{\varphi}_{\sigma\gamma 5} - \tilde{\varphi}_{\sigma\gamma 6}
\end{aligned} \tag{1.4.29}
$$

$$
\begin{aligned}
\tilde{\varphi}_{\mathcal{N}\sigma,\gamma,\sigma} = \;& \tilde{\varphi}_{\sigma\gamma 1}(\tilde{\sigma})_{i+1,j,k}^{a,r,n-1} - \tilde{\varphi}_{\sigma\gamma 2}(\tilde{\sigma})_{i-1,j,k}^{a,r,n} + \tilde{\varphi}_{\sigma\gamma 3}(\tilde{\sigma})_{i,j+1,k}^{a,r,n-1} \\
&- \tilde{\varphi}_{\sigma\gamma 4}(\tilde{\sigma})_{i,j-1,k}^{a,r,n} + \tilde{\varphi}_{\sigma\gamma 5}(\tilde{\sigma})_{i,j,k+1}^{a,r,n-1} - \tilde{\varphi}_{\sigma\gamma 6}(\tilde{\sigma})_{i,j,k-1}^{a,r,n}
\end{aligned} \tag{1.4.30}
$$

where $\sigma = \{\tilde{\ell}, \tilde{b}, \tilde{a}, \tilde{s}\}$, $\gamma = \{\tilde{\ell}, \tilde{b}, \tilde{a}, \tilde{s}, \tilde{r}\}$, and $\widetilde{\mathcal{N}}_\sigma^\gamma$ represents

$$
\begin{aligned}
\left(\widetilde{\mathcal{N}}_\sigma^\gamma\right)_{i,j,k}^{a,r-1} = \;& \left(\widetilde{D}_\sigma\right)_{i,j,k}^{a,r-1} \left(\frac{\tilde{z}_\gamma \, \widetilde{D}_\gamma}{\tilde{z}_\ell^2 \, \widetilde{D}_\ell \, \tilde{\ell} + \tilde{z}_b^2 \, \widetilde{D}_b \, \tilde{b} + \widetilde{D}_a \, \tilde{a} + \tilde{z}_s^2 \, \widetilde{D}_s \, \tilde{s} + \tilde{z}_r^2 \, \widetilde{D}_r \, \tilde{r}}\right)_{i,j,k}^{a,r-1} \\
= \;& \left(\widetilde{D}_\sigma\right)_{i,j,k}^{a,r-1} \left(\frac{\tilde{z}_\gamma \, \widetilde{D}_\gamma}{\widetilde{\mathcal{N}}}\right)_{i,j,k}^{a,r-1} \\
= \;& \left(\widetilde{D}_\sigma\right)_{i,j,k}^{a,r-1} \left(\widetilde{\mathcal{N}}_\gamma\right)_{i,j,k}^{a,r-1} .
\end{aligned} \tag{1.4.31}
$$

Letting σ represent blood (B) and lymphatic (L) vessels, $\tilde{\varphi}_{CheB,taf}$, $\tilde{\varphi}_{CheL,taf}$, $\tilde{\varphi}_{HapB,E}$, $\tilde{\varphi}_{HapL,E}$, $\tilde{\varphi}_{CheB,BnE,taf}$, $\tilde{\varphi}_{CheL,LnE,taf}$, $\tilde{\varphi}_{HapB,BnE,E}$, and $\tilde{\varphi}_{HapL,LnE,E}$ in Eqs. (1.4.23) & (1.4.24) can be decompressed as the following:

$$
\begin{aligned}
\tilde{\varphi}_{Che\sigma,taf} = \;& \Big[A_x\left(\mathcal{A}_{che,\sigma nE}\right)_{i+\frac{1}{2},j,k}^{a,r-1} D_x\left(\widetilde{taf}\right)_{i+\frac{1}{2},j,k}^{a,r,n-1} - A_x\left(\mathcal{A}_{che,\sigma nE}\right)_{i-\frac{1}{2},j,k}^{a,r-1} D_x\left(\widetilde{taf}\right)_{i-\frac{1}{2},j,k}^{a,r,n} \\
&+ A_y\left(\mathcal{A}_{che,\sigma nE}\right)_{i,j+\frac{1}{2},k}^{a,r-1} D_y\left(\widetilde{taf}\right)_{i,j+\frac{1}{2},k}^{a,r,n-1} - A_y\left(\mathcal{A}_{che,\sigma nE}\right)_{i,j-\frac{1}{2},k}^{a,r-1} D_y\left(\widetilde{taf}\right)_{i,j-\frac{1}{2},k}^{a,r,n} \\
&+ A_z\left(\mathcal{A}_{che,\sigma nE}\right)_{i,j,k+\frac{1}{2}}^{a,r-1} D_z\left(\widetilde{taf}\right)_{i,j,k+\frac{1}{2}}^{a,r,n-1} - A_z\left(\mathcal{A}_{che,\sigma nE}\right)_{i,j,k-\frac{1}{2}}^{a,r-1} D_z\left(\widetilde{taf}\right)_{i,j,k-\frac{1}{2}}^{a,r,n}\Big] \\
= \;& \tilde{\varphi}_{Che\sigma 1} - \tilde{\varphi}_{Che\sigma 2} + \tilde{\varphi}_{Che\sigma 3} - \tilde{\varphi}_{Che\sigma 4} + \tilde{\varphi}_{Che\sigma 5} - \tilde{\varphi}_{Che\sigma 6}
\end{aligned} \tag{1.4.32}
$$

$$
\begin{aligned}
\tilde{\varphi}_{Hap\sigma,E} = \;& \Big[A_x\left(\mathcal{A}_{hap,\sigma nE}\right)_{i+\frac{1}{2},j,k}^{a,r-1} D_x\left(\tilde{\phi}_E\right)_{i+\frac{1}{2},j,k}^{a,r,n-1} - A_x\left(\mathcal{A}_{hap,\sigma nE}\right)_{i-\frac{1}{2},j,k}^{a,r-1} D_x\left(\tilde{\phi}_E\right)_{i-\frac{1}{2},j,k}^{a,r,n} \\
&+ A_y\left(\mathcal{A}_{hap,\sigma nE}\right)_{i,j+\frac{1}{2},k}^{a,r-1} D_y\left(\tilde{\phi}_E\right)_{i,j+\frac{1}{2},k}^{a,r,n-1} - A_y\left(\mathcal{A}_{hap,\sigma nE}\right)_{i,j-\frac{1}{2},k}^{a,r-1} D_y\left(\tilde{\phi}_E\right)_{i,j-\frac{1}{2},k}^{a,r,n} \\
&+ A_z\left(\mathcal{A}_{hap,\sigma nE}\right)_{i,j,k+\frac{1}{2}}^{a,r-1} D_z\left(\tilde{\phi}_E\right)_{i,j,k+\frac{1}{2}}^{a,r,n-1} - A_z\left(\mathcal{A}_{hap,\sigma nE}\right)_{i,j,k-\frac{1}{2}}^{a,r-1} D_z\left(\tilde{\phi}_E\right)_{i,j,k-\frac{1}{2}}^{a,r,n}\Big] \\
= \;& \tilde{\varphi}_{Hap\sigma 1} - \tilde{\varphi}_{Hap\sigma 2} + \tilde{\varphi}_{Hap\sigma 3} - \tilde{\varphi}_{Hap\sigma 4} + \tilde{\varphi}_{Hap\sigma 5} - \tilde{\varphi}_{Hap\sigma 6}
\end{aligned} \tag{1.4.33}
$$

$$\tilde{\varphi}_{Che\sigma,\sigma nE,taf} = \tilde{\varphi}_{Che\sigma 1}(\tilde{\sigma}_n^E)_{i+1,j,k}^{a,r,n-1} - \tilde{\varphi}_{Che\sigma 2}(\tilde{\sigma}_n^E)_{i-1,j,k}^{a,r,n} + \tilde{\varphi}_{Che\sigma 3}(\tilde{\sigma}_n^E)_{i,j+1,k}^{a,r,n-1} - \tilde{\varphi}_{Che\sigma 4}(\tilde{\sigma}_n^E)_{i,j-1,k}^{a,r,n} + \tilde{\varphi}_{Che\sigma 5}(\tilde{\sigma}_n^E)_{i,j,k+1}^{a,r,n-1} - \tilde{\varphi}_{Che\sigma 6}(\tilde{\sigma}_n^E)_{i,j,k-1}^{a,r,n} \quad (1.4.34)$$

$$\tilde{\varphi}_{Hap\sigma,\sigma nE,E} = \tilde{\varphi}_{Hap\sigma 1}(\tilde{\sigma}_n^E)_{i+1,j,k}^{a,r,n-1} - \tilde{\varphi}_{Hap\sigma 2}(\tilde{\sigma}_n^E)_{i-1,j,k}^{a,r,n} + \tilde{\varphi}_{Hap\sigma 3}(\tilde{\sigma}_n^E)_{i,j+1,k}^{a,r,n-1} - \tilde{\varphi}_{Hap\sigma 4}(\tilde{\sigma}_n^E)_{i,j-1,k}^{a,r,n} + \tilde{\varphi}_{Hap\sigma 5}(\tilde{\sigma}_n^E)_{i,j,k+1}^{a,r,n-1} - \tilde{\varphi}_{Hap\sigma 6}(\tilde{\sigma}_n^E)_{i,j,k-1}^{a,r,n} \quad (1.4.35)$$

Again, letting σ represent species in Eqs. (1.4.11) – (1.4.17), (1.4.19) – (1.4.21), (1.4.23) & (1.4.24), $(\tilde{\varphi}_\sigma^D)^{a,r-1}$, $\left(\tilde{\varphi}_1^{D,\sigma}\right)^{a,r,n-1}$, and $\left(\tilde{\varphi}_2^{D,\sigma}\right)^{a,r,n}$ terms are given below:

$$(\tilde{\varphi}_\sigma^D)^{a,r-1} = (\tilde{D}_\sigma)_{i-1,j,k}^{a,r-1} + (\tilde{D}_\sigma)_{i,j-1,k}^{a,r-1} + (\tilde{D}_\sigma)_{i,j,k-1}^{a,r-1} + 6\,(\tilde{D}_\sigma)_{i,j,k}^{a,r-1} + (\tilde{D}_\sigma)_{i+1,j,k}^{a,r-1} + (\tilde{D}_\sigma)_{i,j+1,k}^{a,r-1} + (\tilde{D}_\sigma)_{i,j,k+1}^{a,r-1} \quad (1.4.36)$$

$$\left(\tilde{\varphi}_1^{D,\sigma}\right)^{a,r,n-1} = (\tilde{\sigma})_{i+1,j,k}^{a,r,n-1}\left[(\tilde{D}_\sigma)_{i,j,k}^{a,r-1} + (\tilde{D}_\sigma)_{i+1,j,k}^{a,r-1}\right] + (\tilde{\sigma})_{i,j,k+1}^{a,r,n-1}\left[(\tilde{D}_\sigma)_{i,j,k}^{a,r-1} + (\tilde{D}_\sigma)_{i,j,k+1}^{a,r-1}\right] + (\tilde{\sigma})_{i,j+1,k}^{a,r,n-1}\left[(\tilde{D}_\sigma)_{i,j,k}^{a,r-1} + (\tilde{D}_\sigma)_{i,j+1,k}^{a,r-1}\right] \quad (1.4.37)$$

$$\left(\tilde{\varphi}_2^{D,\sigma}\right)^{a,r,n} = (\tilde{\sigma})_{i,j-1,k}^{a,r,n}\left[(\tilde{D}_\sigma)_{i,j,k}^{a,r-1} + (\tilde{D}_\sigma)_{i,j-1,k}^{a,r-1}\right] + (\tilde{\sigma})_{i-1,j,k}^{a,r,n}\left[(\tilde{D}_\sigma)_{i,j,k}^{a,r-1} + (\tilde{D}_\sigma)_{i-1,j,k}^{a,r-1}\right] + (\tilde{\sigma})_{i,j,k-1}^{a,r,n}\left[(\tilde{D}_\sigma)_{i,j,k}^{a,r-1} + (\tilde{D}_\sigma)_{i,j,k-1}^{a,r-1}\right] \quad (1.4.38)$$

Note that the terms $(\tilde{\varphi}_\alpha^k)^{a,r-1}$, $\left(\tilde{\varphi}_1^{k\alpha,p}\right)^{a,r,n-1}$, and $\left(\tilde{\varphi}_2^{k\alpha,p}\right)^{a,r,n}$ in Eq. (1.4.6) are given in the same forms as shown in Eqs. (1.4.36), (1.4.37), and (1.4.38), respectively. Refer to **Supplementary Table 6** for source terms, rates expressions, as well as their corresponding adjustment factors.

In each relaxation sweep, the following parallel and sequential steps take place:

- Solve Eqs. (1.4.1) – (1.4.5) simultaneously for $(\tilde{\phi}_V)_{i,j,k}^{a,r,n}$, $(\tilde{\phi}_D)_{i,j,k}^{a,r,n}$, $(\tilde{\phi}_E)_{i,j,k}^{a,r,n}$, $(\tilde{\mu}_T)_{i,j,k}^{a,r,n}$, and $(\tilde{\mu}_E)_{i,j,k}^{a,r,n}$.
- Solve Eqs. (1.4.6), (1.4.7), (1.4.11) – (1.4.17), and (1.4.19) – (1.4.21) by simple division for $(\tilde{p})_{i,j,k}^{a,r,n}$, $(\tilde{q})_{i,j,k}^{a,r,n}$, $(\tilde{n})_{i,j,k}^{a,r,n}$, $(\tilde{g})_{i,j,k}^{a,r,n}$, $(\tilde{w})_{i,j,k}^{a,r,n}$, $(\tilde{\ell})_{i,j,k}^{a,r,n}$, $(\tilde{b})_{i,j,k}^{a,r,n}$, $(\tilde{a})_{i,j.k}^{a,r,n}$, $(\tilde{s})_{i,j.k}^{a,r,n}$, $(\widetilde{tgf})_{i,j,k}^{a,r,n}$, $(\widetilde{taf})_{i,j,k}^{a,r,n}$, and $(\tilde{m})_{i,j,k}^{a,r,n}$.
- Solve Eqs. (1.4.22) – (1.4.24) by simple division for $(\tilde{F}_E)_{i,j,k}^{a,r,n}$, $(\tilde{B}_n^E)_{i,j,k}^{a,r,n}$, and $(\tilde{L}_n^E)_{i,j,k}^{a,r,n}$.
- Update $(\tilde{\boldsymbol{u}}_\alpha)_{i,j,k}^{a,r,n}$, $(\tilde{\boldsymbol{u}}_\beta)_{i,j,k}^{a,r,n}$, $(\tilde{\boldsymbol{u}}_E)_{i,j,k}^{a,r,n}$, and $(\tilde{r})_{i,j,k}^{a,r,n}$ with Eqs. (1.4.8) – (1.4.10), and (1.4.18).

After $n = \nu$ full relaxation sweeps at a grid level κ, we arrive at $\boldsymbol{\psi}_\kappa^{a,r,\nu}$ represented by

$$\boldsymbol{\psi}_\kappa^{a,r,\nu} = \text{SMOOTH}\left(\nu, \boldsymbol{\psi}_\kappa^{a,r,0}, \boldsymbol{L}_\kappa, \boldsymbol{R}_\kappa\right) \tag{1.4.39}$$

where $\boldsymbol{\psi}_\kappa^{a,r,0}$ is the set of initial values used in the smoother. In the model, the numbers of pre– and post– smoothing cycles may be different and are denoted by ν_1 and ν_2, respectively.

2 Supplementary Tables

Supplementary Table 1 Dimensionless Diffusivities (from (4)).

Dimensionless Parameter	Biological Representation	Scaling Factor *	Value Assigned
$\widetilde{D}_n$	Effective diffusivity of O_2	$D_{n,T}$	computed
$\widetilde{D}_{n,E}$	Diffusivity of O_2 through ECM regions	$D_{n,T}$	1.0
$\widetilde{D}_{n,T}$	Diffusivity of O_2 through tumor regions	$D_{n,T}$	1.0
$\widetilde{D}_{n,H}$	Diffusivity of O_2 through host regions	$D_{n,T}$	1.0
$\widetilde{D}_g$	Effective diffusivity of glucose	$D_{n,T}$	computed
$\widetilde{D}_{g,E}$	Diffusivity of glucose through ECM regions	$D_{n,T}$	1.0
$\widetilde{D}_{g,T}$	Diffusivity of glucose through tumor regions	$D_{n,T}$	1.0
$\widetilde{D}_{g,H}$	Diffusivity of glucose through host regions	$D_{n,T}$	1.0
$\widetilde{D}_w$	Effective diffusivity of CO_2	$D_{n,T}$	computed
$\widetilde{D}_{w,E}$	Diffusivity of CO_2 through ECM regions	$D_{n,T}$	1.0
$\widetilde{D}_{w,T}$	Diffusivity of CO_2 through tumor regions	$D_{n,T}$	1.0
$\widetilde{D}_{w,H}$	Diffusivity of CO_2 through host regions	$D_{n,T}$	1.0
$\widetilde{D}_\ell$	Effective diffusivity of lactate	$D_{n,T}$	computed
$\widetilde{D}_{\ell,E}$	Diffusivity of lactate through ECM regions	$D_{n,T}$	1.0
$\widetilde{D}_{\ell,T}$	Diffusivity of lactate through tumor regions	$D_{n,T}$	1.0
$\widetilde{D}_{\ell,H}$	Diffusivity of lactate through host regions	$D_{n,T}$	1.0
$\widetilde{D}_b$	Effective diffusivity of bicarbonate	$D_{n,T}$	computed
$\widetilde{D}_{b,E}$	Diffusivity of bicarbonate through ECM regions	$D_{n,T}$	1.0
$\widetilde{D}_{b,T}$	Diffusivity of bicarbonate through tumor regions	$D_{n,T}$	1.0
$\widetilde{D}_{b,H}$	Diffusivity of bicarbonate through host regions	$D_{n,T}$	1.0
$\widetilde{D}_a$	Effective diffusivity of H^+ ions	$D_{n,T}$	computed
$\widetilde{D}_{a,E}$	Diffusivity of H^+ through ECM regions	$D_{n,T}$	1.0
$\widetilde{D}_{a,T}$	Diffusivity of H^+ ions through tumor regions	$D_{n,T}$	1.0
$\widetilde{D}_{a,H}$	Diffusivity of H^+ ions through host regions	$D_{n,T}$	1.0
$\widetilde{D}_s$	Effective diffusivity of Na^+ ions	$D_{n,T}$	computed
$\widetilde{D}_{s,E}$	Diffusivity of Na^+ through ECM regions	$D_{n,T}$	1.0
$\widetilde{D}_{s,T}$	Diffusivity of Na^+ ions through tumor regions	$D_{n,T}$	1.0

Dimensionless Parameter	Biological Representation	Scaling Factor *	Value Assigned
$\widetilde{D}_{s,H}$	Diffusivity of Na^{+} ions through host regions	$D_{n,T}$	1.0
$\widetilde{D}_{tgf}$	Effective diffusivity of TGFs	$D_{n,T}$	computed
$\widetilde{D}_{tgf,E}$	Diffusivity of TGFs through ECM regions	$D_{n,T}$	1.0
$\widetilde{D}_{tgf,T}$	Diffusivity of TGFs through tumor regions	$D_{n,T}$	1.0
$\widetilde{D}_{tgf,H}$	Diffusivity of TGFs through host regions	$D_{n,T}$	1.0
$\widetilde{D}_{taf}$	Effective diffusivity of TAFs	$D_{n,T}$	computed
$\widetilde{D}_{taf,E}$	Diffusivity of TAFs through ECM regions	$D_{n,T}$	1.0
$\widetilde{D}_{taf,T}$	Diffusivity of TAFs through tumor regions	$D_{n,T}$	1.0
$\widetilde{D}_{taf,H}$	Diffusivity of TAFs through host regions	$D_{n,T}$	1.0
$\widetilde{D}_{m}$	Effective diffusivity of MDEs	$\mathcal{L}^2/\mathcal{T}$	computed
$\widetilde{D}_{m,E}$	Diffusivity of MDEs through ECM regions	$\mathcal{L}^2/\mathcal{T}$	0.05
$\widetilde{D}_{m,T}$	Diffusivity of MDEs through tumor regions	$\mathcal{L}^2/\mathcal{T}$	0.01
$\widetilde{D}_{m,H}$	Diffusivity of MDEs through host regions	$\mathcal{L}^2/\mathcal{T}$	0.01
$\widetilde{D}_{F}$	Effective diffusivity of Myofibroblastic cells (MFC)	$\overline{\overline{D}}_F$	computed
$\widetilde{D}_{F,E}$	Diffusivity of MFCs through ECM regions	$\overline{\overline{D}}_F$	1.0
$\widetilde{D}_{F,T}$	Diffusivity of MFCs through tumor regions	$\overline{\overline{D}}_F$	0.0
$\widetilde{D}_{F,H}$	Diffusivity of MFCs through host regions	$\overline{\overline{D}}_F$	0.0
$\widetilde{D}_{BnE}$	Effective diffusivity of ECS	$\mathcal{L}^2/\mathcal{T}$	computed
$\widetilde{D}_{BnE,E}$	Diffusivity of ECs through ECM regions	$\mathcal{L}^2/\mathcal{T}$	1.0
$\widetilde{D}_{BnE,T}$	Diffusivity of ECs through tumor regions	$\mathcal{L}^2/\mathcal{T}$	0.0
$\widetilde{D}_{BnE,H}$	Diffusivity of ECs through host regions	$\mathcal{L}^2/\mathcal{T}$	0.0
$\widetilde{D}_{LnE}$	Effective diffusivity of LECs	$\mathcal{L}^2/\mathcal{T}$	computed
$\widetilde{D}_{LnE,E}$	Diffusivity of LECs through ECM regions	$\mathcal{L}^2/\mathcal{T}$	1.0
$\widetilde{D}_{LnE,T}$	Diffusivity of LECs through tumor regions	$\mathcal{L}^2/\mathcal{T}$	0.0
$\widetilde{D}_{LnE,H}$	Diffusivity of LECs through host regions	$\mathcal{L}^2/\mathcal{T}$	0.0

* For example, $\widetilde{D}_n = D_n/D_{n,T}$

Supplementary Table 2 Dimensionless Rate Constants (from (4)).

Dimensionless Parameter	Biological Representation	Scaling Factor *	Value Assigned
$\tilde{\lambda}_{M,V}$	Mitosis rate constant of viable tumor cells	$\lambda_{M,V}$	1.0
$\tilde{\lambda}_{N,V}$	Necrosis rate constant of viable tumor cells	$\lambda_{M,V}$	3.0
$\tilde{\lambda}_{L,D}$	Lysis rate constant of dead tumor cells	$\lambda_{M,V}$	1.0
$\tilde{\lambda}_{F,E}$	ECM rate of secretion by myofibroblastic cells	$\tilde{\phi}_{\alpha}\lambda_{M,V}/F_{max}$	5.0
$\tilde{\lambda}_{de,E}$	Degradation rate of ECM	$\lambda_{M,V}$	1.0
$\tilde{\lambda}_{B,n}$	Apparent transfer coefficient of O_2 via capillary network	$\lambda_{U,V,n}$	computed
$\tilde{\lambda}_{B,n,E}$	Transfer coefficient of O_2 via capillary network in ECM regions	$\lambda_{U,V,n}$	0.1
$\tilde{\lambda}_{B,n,T}$	Transfer coefficient of O_2 via capillary network in tumor regions	$\lambda_{U,V,n}$	0.001
$\tilde{\lambda}_{B,n,H}$	Transfer coefficient of O_2 via capillary network in host regions	$\lambda_{U,V,n}$	0.01
$\tilde{\lambda}_{U,V,n}$	Uptake rate constant of O_2 by viable tumor cells	$\lambda_{U,V,n}$	1.0
$\tilde{\lambda}_{U,H,n}$	Uptake rate constant of O_2 by healthy host cells	$\lambda_{U,V,n}$	0.0001
$\tilde{\lambda}_{B,g}$	Apparent transfer coefficient of glucose via capillary network	$\lambda_{U,V,n}$	computed
$\tilde{\lambda}_{B,g,E}$	Transfer coefficient of glucose via capillary network in ECM regions	$\lambda_{U,V,n}$	0.1
$\tilde{\lambda}_{B,g,T}$	Transfer coefficient of glucose via capillary network in tumor regions	$\lambda_{U,V,n}$	0.001
$\tilde{\lambda}_{B,g,H}$	Transfer coefficient of glucose via capillary network in host regions	$\lambda_{U,V,n}$	0.01
$\tilde{\lambda}_{U,V,g}$	Uptake rate constant of glucose by viable tumor cells	$\lambda_{U,V,n}$	1.0
$\tilde{\lambda}_{U,H,g}$	Uptake rate constant of glucose by healthy host cells	$\lambda_{U,V,n}$	0.0001
$\tilde{\lambda}_{B,w}$	Apparent transfer coefficient of CO_2 via capillary network	$\lambda_{U,V,n}$	computed
$\tilde{\lambda}_{B,w,E}$	Transfer coefficient of CO_2 via capillary network in ECM regions	$\lambda_{U,V,n}$	1.0
$\tilde{\lambda}_{B,w,T}$	Transfer coefficient of CO_2 via capillary network in tumor regions	$\lambda_{U,V,n}$	1.0
$\tilde{\lambda}_{B,w,H}$	Transfer coefficient of CO_2 via capillary network	$\lambda_{U,V,n}$	1.0

Dimensionless Parameter	Biological Representation	Scaling Factor *	Value Assigned
	in host regions		
$\tilde{k}_f$	Forward reaction rate of the dissolution of CO_2 and H_2O	$\lambda_{U,V,n}$	1.0
$\tilde{k}_r$	Backward reaction rate of the dissolution of CO_2 and H_2O	$\lambda_{U,V,n}/n_\infty$	1.0
$\tilde{\lambda}_{B,\ell}$	Apparent transfer coefficient of lactate via capillary network	$\lambda_{U,V,n}$	computed
$\tilde{\lambda}_{B,\ell,E}$	Transfer coefficient of lactate via capillary network in ECM regions	$\lambda_{U,V,n}$	1.0
$\tilde{\lambda}_{B,\ell,T}$	Transfer coefficient of lactate via capillary network in tumor regions	$\lambda_{U,V,n}$	0.1
$\tilde{\lambda}_{B,\ell,H}$	Transfer coefficient of lactate via capillary network in host regions	$\lambda_{U,V,n}$	0.5
$\tilde{\lambda}_{V,tgf}$	Production rate constant of TGFs by viable tumor cells	$\lambda_{U,V,n}$	0.2
$\tilde{\lambda}_{de,tgf}$	Degradation rate constant of TGFs	$\lambda_{U,V,n}$	0.05
$\tilde{\lambda}_{U,V,tgf}$	Uptake rate constant of TGFs by viable tumor cells	$\lambda_{U,V,n}$	0.0
$\tilde{\lambda}_{V,taf}$	Production rate constant of TAFs by viable tumor cells	$\lambda_{U,V,n}$	0.2
$\tilde{\lambda}_{de,taf}$	Degradation rate constant of TAFs	$\lambda_{U,V,n}$	0.05
$\tilde{\lambda}_{U,B,taf}$	Uptake rate constant of TAFs by proliferating ECs	$\lambda_{U,V,n}/B_{max}$	0.0011574
$\tilde{\lambda}_{U,L,taf}$	Uptake rate constant of TAFs by proliferating LECs	$\lambda_{U,V,n}/L_{max}$	0.0011574
$\tilde{\lambda}_{V,m}$	Production rate constant of MDEs by viable tumor cells	$\lambda_{M,V}$	0.2
$\tilde{\lambda}_{de,m}$	Decay rate constant of MDEs	$\lambda_{M,V}$	5.0
$\tilde{\lambda}_{M,FE}$	Mitosis rate constant of MFCs	$\lambda_{M,V}$	0.1
$\tilde{\lambda}_{A,FE}$	Apoptosis rate constant of MFCs	$\lambda_{M,V}$	0.1
$\tilde{\lambda}_{N,FE}$	Necrosis rate constant of MFCs	$\lambda_{M,V}$	0.3
$\tilde{\lambda}_{m,BnE}$	Maximum mitosis rate constant of ECs	$\lambda_{M,V}$	1.0
$\tilde{\lambda}_{crush,BnE}$	Maximum degradation rate constant of new blood vessels due to cell pressure	$\lambda_{M,V}$	1.0
$\tilde{\lambda}_{re,BnE}$	Remodeling rate constant of new blood vessels by	$\lambda_{M,V}/m_{sat}$	1.0

Dimensionless Parameter	Biological Representation	Scaling Factor *	Value Assigned
	MDEs		
$\tilde{\lambda}_{m,LnE}$	Maximum mitosis rate constant of LECs	$\lambda_{M,V}$	1.0
$\tilde{\lambda}_{crush,LnE}$	Maximum degradation rate constant of new lymphatic vessels due to cell pressure	$\lambda_{M,V}$	1.0
$\tilde{\lambda}_{re,LnE}$	Remodeling rate constant of new lymphatic vessels by MDEs	$\lambda_{M,V}/m_{sat}$	1.0

* For example, $\tilde{\lambda}_{N,V} = \lambda_{N,V}/\lambda_{M,V}$.

Supplementary Table 3 Mobility, Motilities, and Taxis Coefficients (from (4)).

Dimensionless Parameter	Biological Representation	Scaling Factor*	Value Assigned
$\tilde{M}$	Mobility of cell species	$\mathcal{M}$	0.1
$\tilde{k}_\alpha$	Motility of the solid phase (cells)	$\bar{\bar{k}}_\alpha$	Computed†
$\tilde{k}_T$	Motility of the tumor cell phase	$\bar{\bar{k}}_\alpha$	10.0
$\tilde{k}_E$	Motility of the ECM phase	$\bar{\bar{k}}_\alpha$	10.0
$\tilde{k}_H$	Motility of the healthy host cell phase	$\bar{\bar{k}}_\alpha$	10.0
$\tilde{k}_\beta$	Motility of the fluid phase (interstitial fluid)	$\bar{\bar{k}}_\beta$	1.0
$\tilde{\chi}_{che,BnE}$	Chemotaxis coefficient of ECs	$\mathcal{L}^2/(\mathcal{T}\ taf_{sat})$	1.0
$\tilde{\chi}_{hap,BnE}$	Haptotaxis coefficient of ECs	$\mathcal{L}^2/(\mathcal{T}\ \tilde{\phi}_\alpha)$	1.0
$\tilde{\chi}_{hap,BnE}^{max}$	Maximum haptotaxis coefficient of ECs	$\mathcal{L}^2/(\mathcal{T}\ \tilde{\phi}_\alpha)$	1.0
$\tilde{\chi}_{hap,BnE}^{min}$	Minimum haptotaxis coefficient of ECs	$\mathcal{L}^2/(\mathcal{T}\ \tilde{\phi}_\alpha)$	1.0
$\tilde{\chi}_{che,LnE}$	Chemotaxis coefficient of LECs	$\mathcal{L}^2/(\mathcal{T}\ taf_{sat})$	1.0
$\tilde{\chi}_{hap,LnE}$	Haptotaxis coefficient of LECs	$\mathcal{L}^2/(\mathcal{T}\ \tilde{\phi}_\alpha)$	1.0
$\tilde{\chi}_{hap,LnE}^{max}$	Maximum haptotaxis coefficient of LECs	$\mathcal{L}^2/(\mathcal{T}\ \tilde{\phi}_\alpha)$	1.0
$\tilde{\chi}_{hap,LnE}^{min}$	Minimum haptotaxis coefficient of LECs	$\mathcal{L}^2/(\mathcal{T}\ \tilde{\phi}_\alpha)$	1.0

All nondimensionalized chemotaxis and haptotaxis coefficients are set to 1 as an initial value in this study. A wider range of values will be tested and analyzed in future work.

* For example, $\tilde{\chi}_{che,BnE} = \chi_{che,BnE}\mathcal{T}\ taf_{sat}/\mathcal{L}^2$.

† The solid phase motility $\tilde{k}_\alpha$ is computed from $\tilde{k}_E$, $\tilde{k}_T$, and $\tilde{k}_H$ using Eq.(2.38) by replacing $\tilde{\psi}_i$ with $\tilde{k}_i$.

Supplementary Table 4 Dimensionless Constants (from (4)).

Dimensionless Constant	Biological Representation	Scaling Factors*	Value Assigned
$\tilde{\epsilon}_T$	Interaction strength for tumor cells	$\bar{\bar{\epsilon}}$	0.05
$\tilde{\epsilon}_E$	Interaction strength for ECM	$\bar{\bar{\epsilon}}$	0.05
$\tilde{\epsilon}_{TE}$	Interaction strength between tumor cells and ECM	$\bar{\bar{\epsilon}}$	0.02
$\tilde{\epsilon}_e$	Strain energy coefficient	$\bar{\bar{\epsilon}}_e$	0.001
$\tilde{n}_h$	Hypoxic level of O_2	n_∞	0.3
$\tilde{n}_C$	O_2 level in capillaries	n_∞	1.0
$\tilde{g}_C$	Glucose level in capillaries	g_∞	1.0
$\tilde{w}_C$	CO_2 level in capillaries	n_∞	0.0
$\tilde{\ell}_C$	Lactate level in capillaries	n_∞	0.0
$\tilde{n}_{v,V}$	O_2 viability limit of viable tumor cells	n_∞	0.21
$\tilde{n}_{v,F}$	O_2 viability limit of MFCs	n_∞	0.21
$\tilde{g}_{v,V}$	Glucose viability limit of viable tumor cells	g_∞	0.1
$\tilde{z}_\ell$	Charge of a lactate ion	z_a	-1.0
$\tilde{z}_b$	Charge of a bicarbonate ion	z_a	-1.0
$\tilde{z}_s$	Charge of Na^+	z_a	1.0
$\tilde{z}_r$	Charge of Cl^-	z_a	-1.0
$\widetilde{tgf}_{FE}$	Threshold level of tgf corresponding to the onset of the upregulation of myofibroblastic cell proliferation	tgf_{sat}	0.1
$\widetilde{taf}_{Bn}$	Threshold level of taf corresponding to the onset of EC proliferation	taf_{sat}	0.2
$\widetilde{taf}_{Ln}$	Threshold level of taf corresponding to the onset of LEC proliferation	taf_{sat}	0.2
$(\tilde{\mathcal{E}}_E^*)_{ij}$	Eigenstrain for the ECM component	$\bar{\bar{\mathcal{E}}}$	1.0
$(\tilde{\mathcal{E}}_C^*)_{ij}$	Eigenstrain for the cell components	$\bar{\bar{\mathcal{E}}}$	0.0
$\tilde{L}_1^E$	Lamé constants for ECM component	L_2^E	1.0
$\tilde{L}_1^C$	Lamé constants for cell components	L_2^E	1.0
$\tilde{L}_2^C$	Lamé constants for cell components	L_2^E	1.0

Dimensionless Constant	Biological Representation	Scaling Factors*	Value Assigned
$(\tilde{\phi}_E)_{min,Bn}$	Concentration of ECM macromolecules corresponding to the minimum EC haptotaxis strength	$\tilde{\phi}_\alpha$	0.2
$(\tilde{\phi}_E)_{max,Bn}$	Concentration of ECM macromolecules corresponding to the maximum EC haptotaxis strength	$\tilde{\phi}_\alpha$	0.8
$(\tilde{\phi}_E)_{min,Ln}$	Concentration of ECM macromolecules corresponding to the minimum LEC haptotaxis strength	$\tilde{\phi}_\alpha$	0.2
$(\tilde{\phi}_E)_{max,Ln}$	Concentration of ECM macromolecules corresponding to the maximum LEC haptotaxis strength	$\tilde{\phi}_\alpha$	0.8
$\tilde{p}_{t,B}$	Threshold pressure corresponding to the onset of blood vessel loss	$\mathcal{P}$	0.6
$\tilde{p}_{t,L}$	Threshold pressure corresponding to the onset of lymphatic vessel loss	$\mathcal{P}$	0.6
$\tilde{p}_{c,Bn}$	Threshold pressure corresponding to the maximum rate of neo-blood vessel loss	$\mathcal{P}$	0.8
$\tilde{p}_{c,Ln}$	Threshold pressure corresponding to the maximum rate of neo-lymphatic vessel loss	$\mathcal{P}$	0.8

* For example, $\tilde{n}_h = n_h/n_\infty$.

Supplementary Table 5 Scaling Factors (from (4)).

Dimensional Scaling Factor	Biological Representation	Expression
$\mathcal{L}$	Characteristic length	$\sqrt{\frac{D_{T,n}}{\lambda_{U,V,n}}}$
$\mathcal{T}$	Characteristic time	$\frac{1}{\lambda_{M,V}}$
$\mathcal{P}$	Characteristic cell pressure	$\frac{\mathcal{L}^2}{\bar{\bar{k}}_\alpha\ \mathcal{T}}$
$\mathcal{Q}$	Characteristic fluid pressure	$\frac{\mathcal{L}^2}{\bar{\bar{k}}_\beta \mathcal{T}}$
$\mathcal{M}$	Characteristic mobility	$\frac{\mathcal{L}^2}{\mathcal{T}\ E_\alpha^*}$
$\bar{\bar{\epsilon}}$	Characteristic interaction strength	$\mathcal{L}\sqrt{\frac{E_\alpha^*}{\tilde{\phi}_\alpha}}$
$\bar{\bar{\mathcal{E}}}$	Characteristic Strain	$\sqrt{\frac{E_\alpha^*\tilde{\phi}_\alpha}{E_e^*\bar{\bar{\epsilon}}L_2^E}}$
$\bar{\bar{D}}_F$	Characteristic Myofibroblastic diffusivity	$\frac{\mathcal{L}^2}{\mathcal{T}\ tgf_{sat}}$

Supplementary Table 6 Source Terms, Rates, and Adjustment Factors (adapted from (4)).

From Equations (2.1):

$$\tilde{S}_V = \tilde{\lambda}_{M,V}\mathcal{A}_{M,V}\tilde{\phi}_V - \tilde{\lambda}_{N,V}\mathcal{A}_{N,V}\tilde{\phi}_V$$

$$\mathcal{A}_{M,V} = \tilde{n}\left(1 + \widetilde{tgf}\right)\mathcal{H}(\tilde{n} - \tilde{n}_h)$$

$$\mathcal{A}_{N,V} = 1 - \mathcal{H}\left(\tilde{n} - \tilde{n}_{v,V}\right)\mathcal{H}\left(\tilde{g} - \tilde{g}_{v,V}\right)$$

From Equation (2.2):

$$\tilde{S}_D = \tilde{\lambda}_{N,V}\mathcal{A}_{N,V}\tilde{\phi}_V - \tilde{\lambda}_{L,D}\mathcal{A}_{L,D}\tilde{\phi}_D$$

$$\mathcal{A}_{L,D} = 1$$

From Equation (2.3):

$$\tilde{S}_E = \tilde{\lambda}_{F,E}\mathcal{A}_{F,E}\tilde{F} - \tilde{\lambda}_{de,E}\mathcal{A}_{de,E}\tilde{\phi}_E$$

$$\mathcal{A}_{F,E} = \left(1 - \tilde{\phi}_T - \tilde{\phi}_E\right)\left(1 + \widetilde{tgf}\right)\left[1 + \mathcal{F}_{n,E}^{F}\frac{\tilde{n}_h - \tilde{n}}{\tilde{n}_h - \tilde{n}_{v,F}}\mathcal{H}(\tilde{n}_h - \tilde{n})\right]\mathcal{H}\left(\tilde{n} - \tilde{n}_{v,F}\right)\mathcal{H}\left(\widetilde{tgf} - \widetilde{tgf}_{F,E}\right)\mathcal{H}\left(1 - \tilde{\phi}_T - \tilde{\phi}_E\right)$$

$$\mathcal{A}_{de,E} = \tilde{m}$$

From Equation (2.22):

$$\tilde{k}_{n1} = \tilde{\lambda}_{B,n}\mathcal{A}_{B,n}$$

$$\tilde{k}_{n2} = \tilde{\lambda}_{U,V,n}\mathcal{A}_{U,V,n} + \tilde{\lambda}_{U,H,n}\mathcal{A}_{U,H,n}$$

$$\mathcal{A}_{B,n} = \mathcal{A}_{B,g} = \mathcal{A}_{B,w} = \mathcal{A}_{B,\ell} = \left(\tilde{B}\right)Q_3\left(1 - \frac{\tilde{p}}{\tilde{p}_{t,B}}\right)\mathcal{H}\left(\tilde{p}_{t,B} - \tilde{p}\right)$$

$$\mathcal{A}_{U,V,n} = \tilde{\phi}_V$$

$$\mathcal{A}_{U,H,n} = \tilde{\phi}_H$$

From Equation (2.23):

$$\tilde{k}_{g1} = \tilde{\lambda}_{B,g}\mathcal{A}_{B,g}$$

$$\tilde{k}_{g2} = \tilde{\lambda}_{U,V,g}\mathcal{A}_{U,V,g} + \tilde{\lambda}_{U,H,g}\mathcal{A}_{U,H,g}$$

$$\mathcal{A}_{B,g} = \left(\tilde{B}\right)Q_3\left(1 - \frac{\tilde{p}}{\tilde{p}_{t,B}}\right)\mathcal{H}\left(\tilde{p}_{t,B} - \tilde{p}\right)$$

$$\mathcal{A}_{U,V,g} = \tilde{\phi}_V$$

$$\mathcal{A}_{U,H,g} = \tilde{\phi}_H$$

From Equation (2.24):

$$\tilde{k}_w = \tilde{\lambda}_{B,w}\mathcal{A}_{B,w}$$

$$\mathcal{A}_{B,w} = (\tilde{B})Q_3\left(1-\frac{\tilde{p}}{\tilde{p}_{t,B}}\right)\mathcal{H}(\tilde{p}_{t,B}-\tilde{p})$$

From Equation (2.25):

$$\tilde{k}_\ell = \tilde{\lambda}_{B,\ell}\tilde{\mathcal{A}}_{B,\ell}$$

$$\mathcal{A}_{B,\ell} = (\tilde{B})Q_3\left(1-\frac{\tilde{p}}{\tilde{p}_{t,B}}\right)\mathcal{H}(\tilde{p}_{t,B}-\tilde{p})$$

From Equation (2.30):

$$\tilde{\lambda}_{tgf} = \tilde{\lambda}_{V,tgf}\mathcal{A}_{V,tgf}$$

$$\tilde{\lambda}_{U,tgf} = \tilde{\lambda}_{U,V,tgf}\mathcal{A}_{U,V,tgf}$$

$$\mathcal{A}_{V,tgf} = \tilde{\phi}_V$$

$$\mathcal{A}_{U,V,tgf} = \tilde{\phi}_V$$

From Equation (2.31):

$$\tilde{\lambda}_{taf} = \tilde{\lambda}_{V,taf}\mathcal{A}_{V,taf}$$

$$\tilde{\lambda}_{U,taf} = \tilde{\lambda}_{U,B,taf}\mathcal{A}_{U,B,taf} + \tilde{\lambda}_{U,L,taf}\mathcal{A}_{U,L,taf}$$

$$\mathcal{A}_{V,taf} = \tilde{\phi}_V\left[1+\mathcal{F}^V_{n,taf}\frac{\tilde{n}_h-\tilde{n}}{\tilde{n}_h-\tilde{n}_{v,V}}\mathcal{H}(\tilde{n}_h-\tilde{n})\right]\mathcal{H}(\tilde{n}-\tilde{n}_{v,V}), \qquad where\ \mathcal{F}^V_{n,taf} = 1$$

$$\mathcal{A}_{U,B,taf} = \tilde{B}_n$$

$$\mathcal{A}_{U,L,taf} = \tilde{L}_n$$

From Equation (2.32):

$$\tilde{S}_m = \tilde{\lambda}_{V,m}\mathcal{A}_{V,m}(1-\tilde{m}) - \tilde{\lambda}_{de,m}\tilde{m} - R_{taf,m}R_\lambda(\tilde{r}_{U,B,taf}+\tilde{r}_{U,L,taf})$$

$$\mathcal{A}_{V,m} = \tilde{\phi}_V$$

$$R_\lambda = 8.64 \times 10^3$$

$$R_{taf,m} = taf_{sat}/m_{sat} = 1$$

From Equation (2.33):

$$\tilde{S}_{FE} = \tilde{\lambda}_{M,FE}\mathcal{A}_{M,FE}\tilde{F}_E - \tilde{\lambda}_{A,FE}\mathcal{A}_{A,FE}\tilde{F}_E - \tilde{\lambda}_{N,FE}\mathcal{A}_{N,FE}\tilde{F}_E$$

$$\mathcal{A}_{M,FE} = (1 - F_E)Q_3\left(\frac{\tilde{n} - \tilde{n}_h}{1 - \tilde{n}_h}\right)\left[1 + \mathcal{F}^{M}_{tgf,F}\ \widetilde{tgf}\ \mathcal{H}\left(\widetilde{tgf} - \widetilde{tgf}_{FE}\right)\right]\mathcal{H}(\tilde{n} - \tilde{n}_h),$$
$$where\ \mathcal{F}^{M}_{tgf,F} = 2$$

$$\mathcal{A}_{N,FE} = 1 - \mathcal{H}\left(\tilde{n} - \tilde{n}_{v,F}\right)\mathcal{H}\left(\tilde{g} - \tilde{g}_{v,F}\right)$$

$$\mathcal{A}_{A,FE} = 1$$

From Equation (2.36):

$\mathcal{A}_{che,BnE} = \mathcal{F}_{Bn}$ where the effect of TAF is not considered.

$$\mathcal{A}_{hap,BnE} = \begin{cases} \tilde{\varpi}_{Bn}\ \mathcal{F}_{Bn} & \tilde{\phi}_E < \left(\tilde{\phi}_E\right)_{min,Bn}\ and\ \tilde{\phi}_E > \left(\tilde{\phi}_E\right)_{max} \\ \left[(1 - \tilde{\varpi}_{Bn})Q_4\left(\frac{\left(\tilde{\phi}_E\right)_{max,Bn} - \tilde{\phi}_E}{\left(\tilde{\phi}_E\right)_{max,Bn} - \left(\tilde{\phi}_E\right)_{min,Bn}}\right) + \tilde{\varpi}_{Bn}\right]\mathcal{F}_{Bn} & \left(\tilde{\phi}_E\right)_{min,Bn} \le \tilde{\phi}_E \le \left(\tilde{\phi}_E\right)_{max,Bn} \end{cases}$$

where $\tilde{\varpi}_{Bn} = \frac{\tilde{\chi}^{min}_{hap,Bn}}{\tilde{\chi}^{max}_{hap,Bn}}$

From Equation (2.37):

$\mathcal{A}_{che,LnE} = \mathcal{F}_{Ln}$ where the effect of TAF is not considered.

$$\mathcal{A}_{hap,LnE} = \begin{cases} \tilde{\varpi}_{Ln}\ \mathcal{F}_{Ln} & \tilde{\phi}_E < \left(\tilde{\phi}_E\right)_{min,Ln}\ and\ \tilde{\phi}_E > \left(\tilde{\phi}_E\right)_{max,L} \\ \left[(1 - \tilde{\varpi}_{Ln})Q_4\left(\frac{\left(\tilde{\phi}_E\right)_{max,Ln} - \tilde{\phi}_E}{\left(\tilde{\phi}_E\right)_{max,Ln} - \left(\tilde{\phi}_E\right)_{min,Ln}}\right) + \tilde{\varpi}_{Ln}\right]\mathcal{F}_{Ln} & \left(\tilde{\phi}_E\right)_{min,Ln} \le \tilde{\phi}_E \le \left(\tilde{\phi}_E\right)_{max,Ln} \end{cases}$$

where $\tilde{\varpi}_{Ln} = \frac{\tilde{\chi}^{min}_{hap,Ln}}{\tilde{\chi}^{max}_{hap,Ln}}$

From Equation (2.34):

$$\tilde{S}_{BnE} = \tilde{\lambda}_{m,BnE}\ \mathcal{A}_{m,BnE}\ \tilde{B}^E_n - \tilde{\lambda}_{re,BnE}\ \mathcal{A}_{re,BnE}\ \tilde{m}\ \tilde{B}^E_n - \tilde{\lambda}_{crush,BnE}\ \mathcal{A}_{crush,BnE}\ \tilde{B}^E_n$$

$$\mathcal{A}_{m,BnE} = \mathcal{F}_{Bn}Q_3\left(\frac{\widetilde{taf} - \widetilde{taf}_{Bn}}{1 - \widetilde{taf}_{Bn}}\right)\mathcal{H}\left(\widetilde{taf} - \widetilde{taf}_{Bn}\right)$$

$$\mathcal{A}_{re,BnE} = \mathcal{F}_{Bn} Q_3 \left(\frac{\widetilde{taf} - \widetilde{taf}_{Bn}}{1 - \widetilde{taf}_{Bn}} \right) \mathcal{H}\left(\widetilde{taf} - \widetilde{taf}_{Bn}\right)$$

$$\mathcal{A}_{crush,BnE} = \mathcal{H}\left(\tilde{p} - \tilde{p}_{c,Bn}\right)$$

From Equation (2.35):

$$\tilde{S}_{LnE} = \tilde{\lambda}_{m,LnE}\, \mathcal{A}_{m,LnE}\, \tilde{L}_n^E - \tilde{\lambda}_{re,LnE}\, \mathcal{A}_{re,LnE}\, \widetilde{m}\, \tilde{L}_n^E - \tilde{\lambda}_{crush,LnE}\, \mathcal{A}_{crush,LnE}\, \tilde{L}_n^E$$

$$\mathcal{A}_{m,LnE} = \mathcal{F}_{Ln} Q_3 \left(\frac{\widetilde{taf} - \widetilde{taf}_{Ln}}{1 - \widetilde{taf}_{Ln}} \right) \mathcal{H}\left(\widetilde{taf} - \widetilde{taf}_{Ln}\right)$$

$$\mathcal{A}_{re,LnE} = \mathcal{F}_{Ln} Q_3 \left(\frac{\widetilde{taf} - \widetilde{taf}_{Ln}}{1 - \widetilde{taf}_{Ln}} \right) \mathcal{H}\left(\widetilde{taf} - \widetilde{taf}_{Ln}\right)$$

$$\mathcal{A}_{crush,LnE} = \mathcal{H}\left(\tilde{p} - \tilde{p}_{c,Ln}\right)$$

3 References

1. Liu XD, Osher S, Chan T. Weighted essentially nonoscillatory schemes. Journal of Computational Physics. 1994;115(1):200-12.
2. Jiang G-S, Shu C-W. Efficient implementation of weighted ENO schemes. Journal of Computational Physics. 1996;126(1):202-28.
3. Wise SM, Lowengrub JS, Cristini V. An adaptive multigrid algorithm for simulating solid tumor growth using mixture models. Math Comput Model. 2011;53(1-2):1-20.
4. Ng CF, Frieboes HB. Model of vascular desmoplastic multispecies tumor growth. Journal of Theoretical Biology. 2017;430:245-82.
